# Common and specific large-scale brain changes in major depressive disorder, anxiety disorders, and chronic pain: a transdiagnostic multimodal meta-analysis of structural and functional MRI studies

**DOI:** 10.1038/s41386-022-01271-y

**Published:** 2022-01-20

**Authors:** Felix Brandl, Benedikt Weise, Satja Mulej Bratec, Nazia Jassim, Daniel Hoffmann Ayala, Teresa Bertram, Markus Ploner, Christian Sorg

**Affiliations:** 1 Technical University of Munich, School of Medicine, Department of Psychiatry, 81675 Munich, Germany; 2 Technical University of Munich, School of Medicine, Department of Neuroradiology, 81675 Munich, Germany; 3 Technical University of Munich, School of Medicine, TUM-NIC Neuroimaging Center, 81675 Munich, Germany; 4 University of Maribor, Faculty of Arts, Department of Psychology, Koroska cesta 160, 2000 Maribor, Slovenia; 5 Technical University of Munich, School of Medicine, Department of Neurology, 81675 Munich, Germany

**Keywords:** Psychiatric disorders, Diseases of the nervous system

## Abstract

Major depressive disorder (MDD), anxiety disorders (ANX), and chronic pain (CP) are closely-related disorders with both high degrees of comorbidity among them and shared risk factors. Considering this multi-level overlap, but also the distinct phenotypes of the disorders, we hypothesized both common and disorder-specific changes of large-scale brain systems, which mediate neural mechanisms and impaired behavioral traits, in MDD, ANX, and CP. To identify such common and disorder-specific brain changes, we conducted a transdiagnostic, multimodal meta-analysis of structural and functional MRI-studies investigating changes of gray matter volume (GMV) and intrinsic functional connectivity (iFC) of large-scale intrinsic brain networks across MDD, ANX, and CP. The study was preregistered at PROSPERO (CRD42019119709). 320 studies comprising 10,931 patients and 11,135 healthy controls were included. Across disorders, *common* changes focused on GMV-decrease in insular and medial-prefrontal cortices, located mainly within the so-called default-mode and salience networks. *Disorder-specific* changes comprised hyperconnectivity between default-mode and frontoparietal networks and hypoconnectivity between limbic and salience networks in MDD; limbic network hyperconnectivity and GMV-decrease in insular and medial-temporal cortices in ANX; and hypoconnectivity between salience and default-mode networks and GMV-increase in medial temporal lobes in CP. *Common* changes suggested a neural correlate for comorbidity and possibly shared neuro-behavioral chronification mechanisms. *Disorder-specific* changes might underlie distinct phenotypes and possibly additional disorder-specific mechanisms.

## Introduction

Major depressive disorder (MDD), anxiety disorders (ANX), and chronic pain (CP) are frequent disorders of brain and behavior [1]. They are closely related due to overlap of changes at different levels—from genetic to brain and symptom level -, high degrees of comorbidity among them, and shared risk factors [2–5]. High comorbidity and overlapping risk factors, such as chronic and/or acute life stress [6], suggest overlapping neural correlates, i.e., similar changes in large-scale brain systems mediating between microscopic alterations and behavioral dysfunctions [7]. Indeed, shared structural [8–10] and functional [11–14] changes in prefrontal-insular circuits, for example, have been reported in all three disorders. In addition, disorder-specific brain-changes have also been reported—for instance, concerning dorsolateral prefrontal cortex (dlPFC) in MDD [15] and distinct amygdala nuclei in ANX [16, 17]. These disorder-specific alterations might reflect specific phenotypes of these three related disorders. However, despite these hints at both common and specific brain changes across disorders, there is still no clear evidence whether and how changes in large-scale brain systems overlap and/or differ consistently (i.e., across multiple studies) in MDD, ANX, and CP. It is important to know such commonalities and differences, as such findings could point to convergent and divergent underlying neuro-behavioral mechanisms, improving the understanding of common and/or disorder-specific treatments (e.g., is there a neurobiological basis for the efficacy of antidepressants in also treating anxiety or pain?) and informing the development of future therapies. The current study addresses exactly this problem via transdiagnostic coordinate-based meta-analysis of brain magnetic resonance imaging (MRI)-studies that compare healthy controls to patients with MDD, ANX, and CP, respectively. We selected a meta-analytic approach since especially for transdiagnostic comparisons, individual studies are often underpowered; meta-analyses can overcome this problem by synthesizing results across studies. Particularly, we investigated properties of intrinsic brain networks, namely intrinsic functional connectivity (iFC), measured by correlated infra-slow blood oxygenation fluctuations of resting-state functional MRI-data [18], and regional gray matter volume (GMV), measured by voxel-based morphometry (VBM) of structural MRI-data [19].

Given this brief outline of our approach, we want to clarify two of its critical points, namely why we restricted our approach to MDD, ANX, and CP, and why we focused on intrinsic brain networks, characterized by GMV and iFC, as neural correlates. Concerning disorder selection, first, comorbidity levels among MDD, ANX, and CP are much higher than those with other disorders: for example, comorbidity between CP and MDD or ANX, respectively, is about 50–60% [2–4], and comorbidity of MDD and ANX is also 50–60% [5]. Comorbidity rates with, for example, other psychiatric disorders are considerably lower, such as substance use disorder 10–30% [2, 3, 5] or schizophrenia and bipolar disorder ca. 30% [5, 20, 21]. Second, MDD, ANX, and CP share major risk factors: for example, chronic and/or acute life stress overlap strongly between MDD, ANX, and CP [6, 13]. Third, high comorbidity without a clear chronological sequence of disorders (MDD can precede CP, but also the other way round) as well as shared risk factors suggest one or several shared neuro-behavioral chronification mechanisms across MDD, ANX, and CP; such shared mechanisms might be complemented by additional distinct neuro-behavioral mechanisms reflecting distinct phenotypes of each disorder. Currently, one of the most promising candidates for a shared mechanism is maladaptive chronified ‘threat behavior’ [13, 22–26] (also called ‘defensive behavior’ [22], “avoidant behavior” [13], or ‘negative-valence behavior’ [27]): in brief, low mood, anxiety, and pain all lead to avoidance and (social) withdrawal and therefore protection from (anticipated) threatening situations [13, 14, 16, 28–30]—if these processes become detached from threats, i.e., if they chronify, they can result in maladaptive threat behavior, such as in MDD, ANX, and CP. Other promising candidates for separate [17, 31, 32] or common basic neuro-behavioral mechanisms in MDD, ANX, and CP comprise, among others, aberrant reward [33] or salience [34] processing. Since these suggested mechanisms usually focus on explaining behavioral traits of depression, anxiety, and pain, most of these models—independent of the exact nature of the underpinning neural mechanism—can be applied best to MDD, ANX, and CP, where these symptoms are more prominent and critical than in other neurological/psychiatric disorders. In this study, we did not directly probe specific behavioral aspects of such supposed neuro-behavioral mechanisms; instead, we focused on common and disorder-specific large-scale neural correlates, which allow to speculate about possible common and disorder-specific neuro-behavioral mechanisms supported by these neural correlates, thereby preparing future studies.

Concerning neural correlates, we focused on intrinsic brain networks, i.e., highly consistent whole-brain patterns of coherent ongoing brain activity, such as the so-called default-mode network [35, 36]. Intrinsic brain networks can be characterized by iFC among constituting regions and by GMV of these regions. We focused on intrinsic networks characterized by iFC and GMV since these neural correlates are large-scale and comparatively stable, both reflecting a “history” of acquired/learned (impairments of) behavior and shaping future behavior [37]; therefore, they might mediate between microscopic mechanistic changes and behavioral functions and are predictive for longer-term behavioral traits such as low mood or anxiety [7]. For this reason, task-based activation data, which reflect rather short-term reactions to external stimuli, were not investigated. Furthermore, other measures characterizing intrinsic networks, like structural connectivity, were not considered to keep pairwise multi-modal overlap analyses simple. Regarding iFC, we restricted our approach to whole-brain seed-to-voxel iFC-data as a kind of “simple” iFC, since more complex iFC-approaches, like graph analysis or dynamic iFC, cannot be integrated easily into meta-analyses of network-iFC.

We tested (i) which changes of regional GMV and network-iFC are *common* across MDD, ANX, and CP; (ii) which changes are *specific* (i.e., significantly more pronounced) to one of the three disorders; and (iii) whether and where specific GMV- and iFC-changes overlap [38]. This approach is ‘transdiagnostic’ since it respects current diagnostic categories of MDD, ANX, and CP (while acknowledging their limitations), but also looks for commonalities across these categories [39].

## Materials and methods

To test our hypotheses, we employed well-established Multilevel Kernel Density Analysis (MKDA) for coordinate-based meta-analysis of brain imaging-studies (open-source code available at https://github.com/canlab/Canlab_MKDA_MetaAnalysis) [17, 40]. We followed the analytic approach of grouping seed-based iFC-effects according to “seed networks”, which was first introduced by [11] and since then applied by several studies [12, 38, 41]. Since we [38] and others have already described this approach in detail, we only give a brief description of our current approach (details in Supplementary Methods).

### Literature search and study selection

**Fig. 1:**
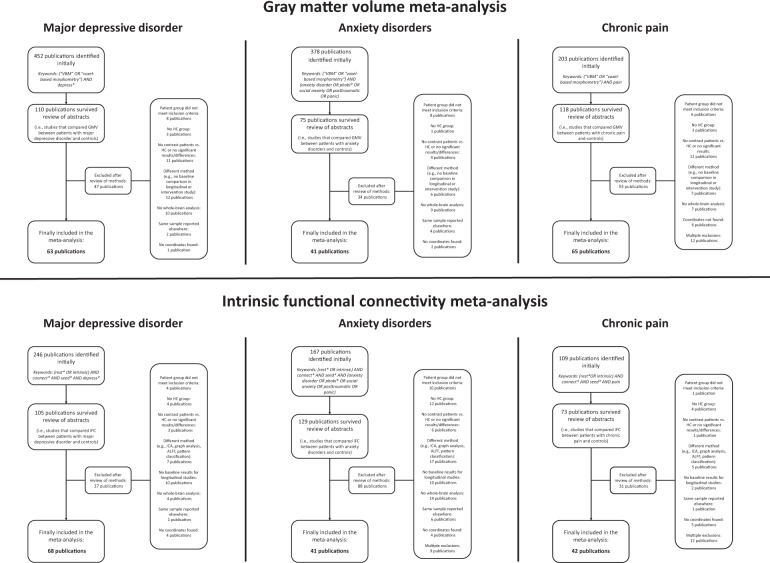
Flow diagram of literature search. ALFF amplitude of low-frequency fluctuations, GMV gray matter volume, HC healthy controls, ICA independent component analysis, iFC intrinsic functional connectivity, VBM voxel-based morphometry.

The meta-analysis was registered at PROSPERO (registration number: CRD42019119709; https://www.crd.york.ac.uk/PROSPERO/display_record.php?RecordID=119709). Studies were searched until January 01, 2020 in PubMed, Web of Science, EMBASE, and reference lists of reviews and eligible articles, using the keywords (rest* OR intrinsic) AND connect* AND seed* AND [disorder name] for the iFC meta-analysis and *(“VBM” OR “voxel-based morphometry”) AND [disorder name]* for the GMV meta-analysis (disorder-specific details in Table S1). All English-language publications of *whole-brain VBM* and *whole-brain seed-to-voxel iFC* comparing patients with MDD, ANX, or CP (with explicit diagnosis of the respective disorder, e.g., using DSM-5) to healthy subjects were selected following MOOSE guidelines for meta-analyses of observational studies (Fig. 1, Table S2) [42]; furthermore, a PRISMA checklist is attached. Briefly, exclusion criteria were (i) methods other than VBM or seed-based iFC; (ii) no whole-brain analysis; (iii) neurological (other than CP) or severe medical comorbidity (psychiatric comorbidity was no exclusion criterion); (iv) no peak-coordinates reported in standard space. In longitudinal or intervention/challenge studies, only baseline results were considered. We made no restrictions concerning age, illness duration, symptom severity, or medication status to ensure maximal study coverage, but we conducted several control analyses later on. For further details about inclusion/exclusion criteria, study quality control, and control variables, see Supplementary Methods.

### Data extraction

For the GMV-meta-analysis, peak-coordinates of between-group effects were extracted from included GMV-studies (Tables S3–S5). For the iFC-meta-analysis, we took the following previously-used approach to meta-analytically investigate network-iFC based on whole-brain seed-to-voxel-iFC input studies (Tables S6–S8) [11, 38]: first, we extracted the center-coordinates of seed regions-of-interest from included iFC-studies. Second, we assigned each seed—based on its anatomical location—to one out of seven networks from a widely-used parcellation based on iFC-data from 1000 healthy subjects, comprising visual (VIS), primary-sensorimotor (PSM), dorsal attention (DAN), salience (SAL), limbic (LIM), frontoparietal (FPN), and default-mode (DMN) networks (Fig. S1) [36, 43, 44]. Since no similar network-parcellation was available for thalamus, studies using only thalamus or hypothalamus seeds were excluded (3 studies in total). Third, peak-coordinates of iFC between-group effects were extracted from included studies and grouped according to the network-assignment of their respective seed, yielding one list of between-group iFC-effects per network.

### Meta-analysis

MKDA meta-analysis of both GMV and network-iFC was conducted [11, 40] following our previous work on transdiagnostic multimodal meta-analysis of structural and resting-state fMRI-studies across psychiatric disorders [38]. For iFC, meta-analyses were only conducted for networks with at least three studies in each disorder to ensure sufficient power. For details on MKDA-meta-analysis, please see Supplementary Methods. MKDA yields result-maps which show a “density statistic” as effect size, reflecting the number of studies that found a group-difference at a particular brain location. Clusters with significantly aberrant GMV or seed-network-related iFC at *p* < 0.05 (FWER-corrected for false-positive results from multiple testing) are identified using Monte-Carlo simulations (15,000 iterations) [40]; clusters significant both based on density statistic (height-based threshold, p < 0.05 FWER) and based on cluster size (extent-based threshold, *p* < 0.05 FWER) are reported, for these thresholds convey complementary information [11, 38]. We tested for *common* (i.e., observed in all three disorders) and *specific* (i.e., more pronounced in one disorder than in the other two disorders) changes in MDD, ANX, and CP.

*GMV-changes and seed-network-related iFC-changes common to MDD, ANX, and CP* were identified via a two-step procedure: (i) Identification of consistent GMV-increase/decrease and iFC-hyper/hypoconnectivity, respectively, separately for MDD, ANX, and CP compared to healthy controls, using MKDA-meta-analysis (p < 0.05 FWER-corrected). (ii) Conjunction analysis to detect common GMV-increase/decrease and iFC-hyper/hypoconnectivity across MDD, ANX, and CP (*p* < 0.0015; details in Supplementary Methods—Details of conjunction analyses) [38, 45, 46].

*GMV-changes and seed-network-related iFC-changes specific to MDD, ANX, and CP* were also identified via a two-step procedure: (i) Pairwise direct contrasts between single-disorder effects (e.g., more pronounced iFC-hyperconnectivity in MDD compared to ANX, i.e., MDD > ANX, or more correctly “(MDD > HC) > (ANX > HC)”), using MKDA-meta-analysis and subsequent conjunction (*p* < 0.005; Supplementary Methods) [45]. (ii) Contrasts between one disorder and both other disorders (e.g., specific iFC-hyperconnectivity in MDD compared to *both* ANX and CP), using conjunction across pairwise contrasts (i.e., conjunction between MDD > ANX and MDD > CP) (p < 0.00005; Supplementary Methods) [38, 45]. Thus, disorder-specific effects reflected, for example, regions in which MDD > HC hyperconnectivity was significantly more frequent across input studies than both ANX > HC and CP > HC hyperconnectivity.

Finally, *regional overlap between specific GMV-changes and specific seed-network-related iFC-changes* was detected via conjunction of specific iFC-changes and specific GMV-changes (*p* < 3 × 10^−9^; Supplementary Methods) [38, 45].

### Control analyses

We controlled for disproportionate influences of single studies and partial non-independence across studies (i.e., jackknife analyses), demographic (e.g., age, gender) and methodological factors (e.g., medication, global-signal-regression for iFC-analysis, modulation during VBM) via χ²-tests (details in Supplementary Methods) [11, 17, 38]. Furthermore, we controlled for comorbidity across MDD, ANX, and CP by testing whether meta-analytic result-clusters showed significant differences in density-statistic when comparing studies *with* comorbidity to studies *without* comorbidity via χ²-tests (Supplementary Methods). Comorbidity information was reported by about 85% of studies (Tables S3–S8); comorbidity across included disorders was present in about one third of studies (Table S9). Finally, as MKDA meta-analysis cannot deal with studies reporting no significant results (Table S10 lists these studies), we used “Seed-based d-Mapping with Permutation of Subject Images (SDM-PSI)” to test whether excluding these studies biased results (Supplementary Methods) [47].

## Results

### Included studies

Concerning GMV, 63 MDD-studies (2934 patients/3284 controls), 41 ANX-studies (1021/1130), and 65 CP-studies (2185/2358) were included (Tables S3–S5). Concerning iFC, 68 MDD-studies (2314/2141), 41 ANX-studies (1248/1108), and 42 CP-studies (1229/1114) were included (Tables S6–S8).

While *common* and *specific* GMV- and iFC-changes across MDD, ANX, and CP are reported next, *pairwise* contrasts between single disorders and healthy controls as well as across disorder pairs are described in Supplementary Results, Figs. S2–S5, and Tables S12, S14, S16, S18, which show p-values and density statistics representing effect sizes.

### GMV-changes

#### Common GMV-changes

**Fig. 2:**
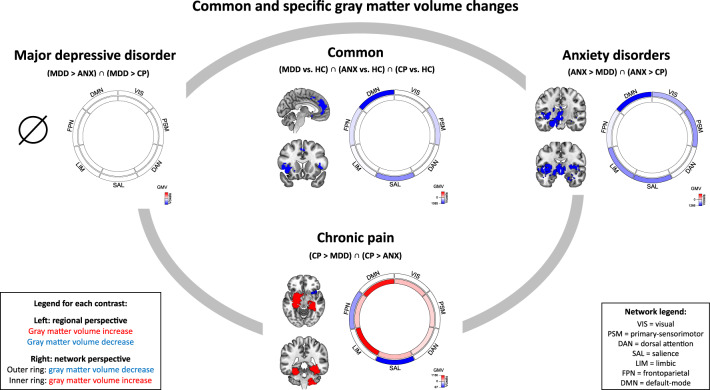
Common and specific gray matter volume changes. *Specific* gray matter volume changes are depicted along the gray oval line; they were calculated by pairwise MKDA meta-analytic contrasts (e.g., MDD > ANX and MDD > CP) and subsequent conjunction across these pairwise result maps (*p* < 0.00005). *Common* gray matter volume changes across MDD, ANX, and CP are depicted in the center of the gray oval; they were calculated by separate MKDA meta-analytic contrasts of each disorder vs. healthy controls (e.g., MDD > HC) and subsequent conjunction across single-disorder results (*p* < 0.0015). For each contrast, meta-analytic regional result clusters are shown on the left. Their overlap with intrinsic brain networks [36, 43, 44] is displayed on the right: GMV-decrease is shown in the outer ring, GMV-increase in the inner ring of each diagram; color intensity reflects the size of spatial overlap (the more voxels, the stronger the color—a colorscale is added to each plot). ANX anxiety disorder, CP chronic pain, HC healthy controls, MDD major depressive disorder.

Common GMV-decreases in MDD, ANX, and CP were found in bilateral insula (left > right), dorsomedial PFC (dmPFC), bilateral anterior cingulate cortex (ACC), supplementary motor area, superior temporal gyrus (STG), and lateral PFC. GMV-decreases were mostly located in DMN (57%), SAL (26%), and PSM (10%) (networks with less than 10% overlap are not reported) (Fig. 2; Table S13 shows p-values). There were no significant clusters of common GMV-increase. As control, we also calculated conjunctions across pairs of disorders to compute pairwise overlaps of GMV-changes (Supplementary Results, Figs. S6–S9).

#### Specific GMV-changes

***MDD****.* No specific GMV-changes were identified (Fig. 2, Table S15).

***ANX****.* We found specific GMV-decreases in bilateral hypothalamus, thalamus (mediodorsal, ventral lateral, and ventral anterior nuclei [48]), striatum (putamen, ncl. accumbens), and insula (left: whole, right: posterior), right amygdala (mainly centromedial/superficial nuclei [49]), and left hippocampus (anterior and posterior), parahippocampal gyrus, temporal pole, and ventrolateral PFC (vlPFC). GMV-decreases were mainly located in DMN (37%), SAL (19%), LIM (16%), PSM (15%), and VIS (10%) (Fig. 2, Table S15).

***CP****.* Specific GMV-increases were detected for bilateral hippocampus (left: whole, right: posterior), parahippocampal gyrus, and ncl. accumbens, left amygdala (basolateral and centromedial [49]) and putamen, as well as right cerebellar hemisphere. Results were mainly located in LIM (33%) and DMN (31%). Specific GMV-decreases were found in right anterior insula, mainly located in SAL (66%) and FPN (27%) (Fig. 2, Table S15).

### iFC-changes

IFC-meta-analyses were conducted for PSM, SAL, LIM, FPN, and DMN networks, because common GMV-decrease overlapped with these networks (Fig. 2) and at least three studies per disorder were available for them (Table S11) [11, 38].

#### Common iFC-changes

**Fig. 3:**
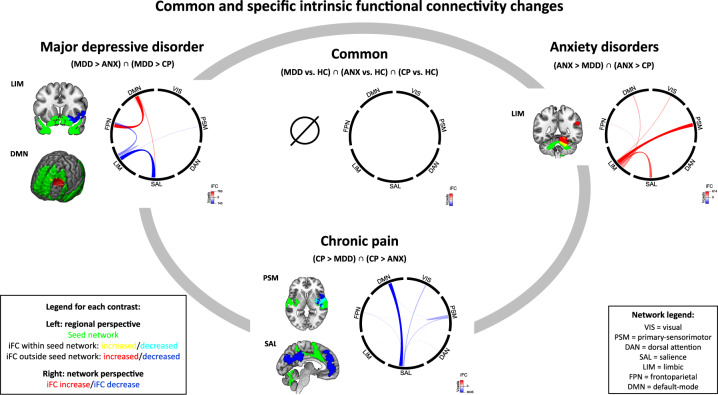
Common and specific intrinsic functional connectivity changes. *Specific* intrinsic functional connectivity changes are depicted along the gray oval line; they were calculated by pairwise MKDA meta-analytic contrasts (e.g., MDD > ANX and MDD > CP) and subsequent conjunction across these pairwise result maps (*p* < 0.00005). *Common* intrinsic functional connectivity changes across MDD, ANX, and CP are depicted in the center of the gray oval; they were calculated by separate MKDA meta-analytic contrasts of each disorder vs. healthy controls (e.g., MDD > HC) and subsequent conjunction across single-disorder results (*p* < 0.0015). For each contrast, meta-analytic regional result clusters are shown on the left. Their overlap with intrinsic brain networks [36, 43, 44] is displayed on the right in a “chord diagram” [67]: between-network connectivity is shown as links, within-network connectivity as “hills”; both color intensity and link thickness reflect the size of spatial overlap (the more voxels, the stronger the color and the thicker the link—a colorscale is added to each plot). ANX  anxiety disorder, CP  chronic pain, HC healthy controls, iFC intrinsic functional connectivity, MDD major depressive disorder.

We detected no significant clusters of common hyper- or hypoconnectivity across disorders (Fig. 3, Table S17). As control, pairwise conjunctions showed, exclusively, overlapping LIM-hypoconnectivity in right insula for MDD and ANX and in left medial PFC for ANX and CP (Figs. S10–S11).

#### Specific iFC-changes

***MDD****.* We identified specific hyperconnectivity between DMN and left dlPFC. DMN-hyperconnectivity was located in FPN (70%), SAL (18%), and DMN (12%), indicating DMN-FPN, DMN-SAL, and within-DMN-hyperconnectivity (Fig. 3, Table S19). Specific hypoconnectivity was observed between LIM and right ventral putamen, amygdala (mostly basolateral, also parts of centromedial [49]), and anterior insula. LIM-hypoconnectivity was mainly located in SAL (53%), FPN (25%), and LIM (15%), indicating LIM-SAL, LIM-FPN and within-LIM-hypoconnectivity.

***ANX****.* Specific hyperconnectivity was detected between LIM and right cerebellum, angular/supramarginal gyrus, and occipital cortex. LIM-hyperconnectivity was mainly located in PSM (39%), SAL (26%), LIM (14%), and DMN (10%), indicating LIM-PSM, LIM-SAL, within-LIM, and LIM-DMN-hyperconnectivity (Fig. 3, Table S19).

***CP****.* We found specific hypoconnectivity between SAL and bilateral dmPFC, ACC, posterior cingulate cortex, precuneus, and cuneus. SAL-hypoconnectivity was located mainly in DMN (72%) and VIS (16%), indicating SAL-DMN and SAL-VIS-hypoconnectivity. Further specific hypoconnectivity was identified between PSM and right posterior insula, putamen, STG, and vlPFC. PSM-hypoconnectivity was located mainly in PSM (54%), SAL (28%), and DMN (11%), indicating within-PSM, PSM-SAL, and PSM-DMN-hypoconnectivity (Fig. 3, Table S19).

### Regional overlap between specific GMV-changes and specific iFC-changes

Only for CP, we identified one small overlapping cluster (18 voxels): specific GMV-decrease converged with specific PSM-FPN-hypoconnectivity on right vlPFC (Fig. S12, Table S20).

### Control analyses

Jackknife analyses showed no disproportionate influence of any single study on results (χ²-tests: *p* ≥ 0.85 in the GMV-meta-analysis, *p* ≥ 0.51 in the iFC-meta-analysis), meaning that the density-statistic of each significant result cluster did not change significantly after iteratively leaving out one study. Results were not significantly influenced by comorbidity across included disorders (GMV: *p* ≥ 0.60, iFC: *p* ≥ 0.28), age (GMV: *p* ≥ 0.16, iFC: *p* ≥ 0.30; influence of non-adult studies: GMV: *p* ≥ 0.85, iFC: *p* ≥ 0.84), gender (GMV: *p* ≥ 0.52, iFC: *p* ≥ 0.53), or medication (GMV: *p* ≥ 0.48, iFC: *p* ≥ 0.37), nor by methodological factors like modulation during VBM (*p* ≥ 0.64) or global-signal-regression during iFC-analysis (*p* ≥ 0.63). Moreover, including studies without significant results did not change meta-analysis results (Fig. S13).

### Multimodal synopsis: common and specific large-scale brain changes

**Fig. 4:**
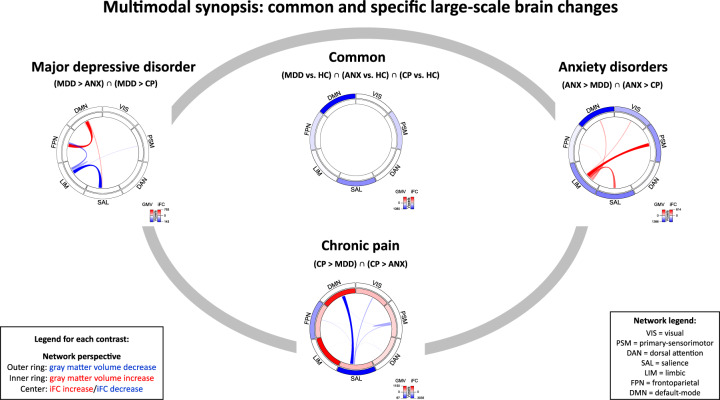
Multimodal synopsis: common and specific patterns of large-scale brain changes. Overlap of *common* and *specific* GMV-changes (from Fig. 2) and iFC-changes (from Fig. 3) with intrinsic brain networks [36, 43, 44] is depicted in one “chord diagram” [67] per contrast. The rings of each diagram reflect GMV-changes: GMV-decrease is shown in the outer ring, GMV-increase in the inner ring; color intensity reflects the size of the spatial overlap (the more voxels, the stronger the color—a colorscale is added to each plot). In the center of each diagram, iFC-changes are displayed: between-network connectivity is shown as links, within-network connectivity as “hills”; both color intensity and link thickness reflect the size of the spatial overlap (the more voxels, the stronger the color and the thicker the link—a colorscale is added to each plot). ANX anxiety disorder, CP chronic pain, HC healthy controls, iFC intrinsic functional connectivity, MDD major depressive disorder.

Results from a network perspective are summarized in Fig. 4, representing a synopsis of results from Figs. 2 and 3, thus providing an overview of which networks were multimodally affected.

## Discussion

Using coordinate-based meta-analysis, we provide first-time evidence for common and specific large-scale brain changes in major depression, anxiety disorders, and chronic pain. Common changes concerned gray matter volume loss in insular and prefrontal cortices of default-mode and salience networks, suggesting a neural correlate for comorbidity and possibly shared chronification mechanisms. Specific gray matter volume and intrinsic functional connectivity changes of each disorder concerned default-mode, salience, limbic and sensorimotor networks; these changes might underlie distinct phenotypes and suggest additional disorder-specific mechanisms.

### Common GMV-decrease across major depression, anxiety disorders, and chronic pain

We found common GMV-decreases in insula and dorsomedial prefrontal/anterior cingulate cortices, mostly located within DMN and SAL networks (Fig. 2). Leave-one-out jackknife and post-hoc control analyses showed that results were not significantly influenced by any single study, comorbidity across included disorders, demographic/clinical variables (age, gender, medication), or methodological issues (modulation during VBM, global-signal-regression, non-significant studies).

Our result of common GMV-decrease facilitates new mechanistic and clinical insights. First, our study extends previous single-disorder meta-analyses of GMV-changes in MDD [8], ANX [9], and CP [10]: via conjunction analysis, we showed directly, for the first time to our knowledge, an overlap between disorder-individual GMV-decreases in medial PFC/ACC and insula, which had already been shown individually for the disorders; on the other hand, there was no overlap of GMV-increase.

High comorbidity levels across MDD, ANX, and CP (50–60% [2–5]) without a clear chronological sequence of disorders (MDD can precede CP, but also the other way round) as well as shared risk factors suggest shared neuro-behavioral chronification mechanisms and underlying neural correlates. Common GMV-decrease might represent just such a neural correlate of high comorbidity, possibly reflecting a ‘common core’ of large-scale brain changes across the three disorders. This ‘common core’ might predispose/increase the probability for further disorder-specific changes (possibly underlying distinct disorder phenotypes) in the future, leading to further disorder(s) as comorbidity.

Second, common GMV-decrease in medial PFC/ACC and insula suggests shared neurobehavioral disease-mechanisms across MDD, ANX, and CP. One (but not the exclusive) candidate for such a common mechanism is maladaptive chronified threat behavior [13, 22–26], since medial PFC and insula have been implicated in both the *learning/persistence* and the *control* of human threat behavior [46, 50]. Therefore, impairments of medial PFC and insula circuits might play an important role in the chronification and maintenance of negative moods, anxiety, and pain – presumably via impaired control/downregulation of threat behavior –, leading to MDD, ANX, and CP, respectively. Other possible candidates for common mechanisms comprise aberrant reward processing [33] or aberrant salience processing [34]. For example, a recent review posited that deficits in reward processing and learning, as shared vulnerability factors for MDD, ANX, and CP, underlie negative moods, anxiety, and reduced pain mitigation by external rewards, and are associated with a brain network comprising medial PFC and cingulo-insular cortices [33]. Since we did not directly test for neuro-behavioral mechanisms in our study, our results should mainly be seen as a starting point for future studies specifically testing these hypotheses in MDD, ANX, and CP.

Third, our study extends and contrasts with recent transdiagnostic meta-analyses of a wider range of psychiatric disorders (e.g., comparing MDD and ANX with schizophrenia or bipolar disorder instead of CP) [38, 41, 51]: (i) we also included chronic pain studies—although psychiatric disorders and chronic pain are intimately related, they are often addressed by distinct research communities, which our work tries to integrate; (ii) we restricted disorder selection to MDD, ANX, and CP (excluding, e.g., schizophrenia or bipolar disorder) based on higher comorbidity among themselves than with other disorders, shared risk factors, and possible mechanistic overlaps; (iii) we combined GMV- and iFC-analyses in one framework. We found common GMV-changes across MDD, ANX, and CP in insular, medial-prefrontal, and cingulate cortices. These changes are also present across several other psychiatric disorders, e.g., also in schizophrenia [41, 51], possibly hinting at brain region-pleiotropy, i.e., many-to-many-mappings between brain structure/systems/circuits and behavioral/cognitive functions [52]. For example, functions associated with insula and cingulate cortex are allostasis, interoception, and detection of salient stimuli [34, 53], which are important for threat behavior, but also, for example, for reward processing. Hence, these regions are implicated in various disorders.

### No common iFC-changes

In contrast with common GMV-decrease, we identified no common iFC-changes (Fig. 3). Single-disorder meta-analytic iFC-changes (the basis for transdiagnostic tests) were largely well consistent with previous meta-analyses investigating iFC-changes in MDD, ANX, and CP separately [11, 12], confirming the reliability of our approach (Supplementary Discussion). So why were there no common iFC-changes across all three disorders? From a general methodological perspective, firstly, GMV and iFC reflect distinct brain features or clusters of features (i.e., single-region brain structure vs. bi-/multi-regional correlated blood oxygenation), which are not necessarily correlated, particularly since some disorders might be impaired in only one feature but not in the other. So, if there is no multi-modal overlap of changes, one can only conclude that distinct regions are aberrant regarding different features. Secondly, underlying factors of blood oxygenation-based iFC are heterogeneous: beyond neuronal processes, a couple of hemodynamic, vascular, and mediating control processes (e.g., astrocytes) underpin general neuro-vascular coupling between neuronal activity and blood oxygenation, which underlies BOLD iFC [54]. These factors might distinctively contribute to aberrant iFC for different disorders, networks, or regions, inducing larger heterogeneity for iFC-findings than for GMV-findings, which in turn might prevent common iFC-changes across all three disorders.

Explaining the absence of common iFC-changes concretely with our data, we firstly see that the spatial outline of iFC-changes is distinct across disorders, i.e., across disorders, mostly different networks are affected. This distinct spatial outline is independent of network size or statistical power, since at least for default-mode network (≥14 studies for each disorder) and salience network (≥10 studies per disorder), power was well sufficient. Methodological heterogeneity is also a possible, but unlikely explanation, since methods of iFC-calculation/preprocessing did not substantially differ across disorders or were controlled for in our control analyses. A possible functional implication of distinct iFC-changes could be that iFC might reflect disorder-specific functional impairments/phenotypes that are associated with given networks. This – albeit speculative - interpretation is supported by the observation of disorder-specific iFC-changes. Secondly, the strength and/or direction of iFC-changes differed across disorders, for example, for the auditory-sensorimotor network, we found hyperconnectivity in MDD, but hypoconnectivity in CP. This is a difficult topic as we do not have a clear interpretation of the quantitative nature of iFC-changes; therefore, we have to abstain from a functional interpretation.

Taken together, the absence of common iFC-changes might derive from a combination of factors, which should be clarified by future studies. Also, the relation between iFC and threat behavior should be tested directly in the future.

### Specific GMV- and iFC-changes

In MDD, specific iFC-changes focused on DMN-FPN-hyperconnectivity (regionally located in dlPFC) and LIM-SAL-hypoconnectivity (in insula and ventral striatum) (Figs. 2–4). In ANX, specific changes focused on GMV-decrease in amygdala, hypothalamus, thalamus, striatum, hippocampus, and insula (overlapping with DMN, SAL, LIM and PSM), and LIM-hyperconnectivity (predominantly with PSM and SAL, results located in parietal and cerebellar cortices). In CP, we found specific GMV-increase in hippocampus, amygdala, cerebellum, and ventral striatum (overlapping mainly with LIM and DMN) as well as GMV-decrease in insula (overlapping mostly with SAL and to a lesser degree with FPN), while specific iFC-changes centered on SAL-DMN-hypoconnectivity (in prefrontal-cingulate cortices) and to a lesser degree also on within-PSM-hypoconnectivity (in posterior insula and putamen).

Although disorder-specific results were largely consistent with previous single-disorder meta-analyses that separately compared MDD, ANX, and CP to healthy controls regarding GMV [8–10] and iFC [11, 12] (for details, see Supplementary Discussion), the direct comparisons between disorders are, to our knowledge, novel. Three observations require further discussion: first, we observed no specific GMV-changes in MDD, although GMV-changes in MDD versus healthy subjects (Figure S2, Supplementary Results and Discussion) were well compatible with previous meta-analyses [8, 55, 56]. The lack of MDD-specific findings appears to derive from the fact that GMV-changes in MDD overlapped strongly with changes in ANX and CP (all vs. healthy controls, respectively), hence no GMV-changes *specific* to MDD. From a more general perspective, the lack of specificity might derive from heterogeneity of MDD symptoms and their strong overlap with other disorders. Indeed, some meta-analyses detected no or only small converging effects across studies in MDD concerning GMV and also other measures like brain activity related to cognitive and emotional processing [57, 58].

Second, specific GMV-decrease in ANX overlapped with common GMV-decrease across disorders: within our concept of specificity, this means that GMV-decrease in ANX vs. HC overlapped with the other disorders’ changes vs. HC, but was significantly more pronounced. Future studies might further clarify the specificity of this finding, whether it is consistent across the course of ANX, and whether it is related to phenotypic specificity.

Third, we found specific GMV-*increase* in CP only, and particularly in hippocampus and amygdala, where ANX showed specific GMV-*decrease*. The functional significance of this difference remains to be investigated; however, recent data and meta-analyses suggest a prominent role of medial-temporal areas, particularly the hippocampus, in CP pathophysiology. For example, medial-temporal volume and pain-related activity are associated with CP intensity [59]. Interestingly, such a medial-temporal GMV-increase might have some potential for being a contrasting marker between CP and other psychiatric disorders; future comparison studies are needed to test its distinctive potential.

Regarding the functional relevance of disorder-specific findings, we can only speculate, since we did not test the functional implications of these findings directly: disorder-specific changes might reflect distinct phenotypes of the disorders, which might be subserved by differential neuro-behavioral mechanisms complementing shared mechanisms. When considering maladaptive threat behavior as a possible shared mechanism, one could think of differentially pronounced impairments of parts of threat behavior [22]: e.g., Pavlovian fear conditioning, associated with, e.g., insular and cingulate cortices [60], which are more affected in ANX, or the cognitive regulation of aversive emotional states, often associated with prefrontal cortices [22, 46], which are more affected in MDD and CP. Future transdiagnostic functional studies are necessary to test these assumptions. Until then, several candidate interpretations and mechanisms remain possible, for example also negative valence [27] or a so-called ‘pain network’ [61, 62], which overlaps with CP-specific changes.

### Limitations

First, disorder-specific effects were calculated by directly contrasting single-disorder effects, which were based on comparisons vs. healthy controls. However, the identification of disorder-specific abnormalities is optimized in meta-analyses that draw upon empirical studies that, themselves, directly compared between diagnostic groups. Therefore, future meta-analyses focusing on transdiagnostic studies are needed, although it will currently be difficult to find enough input studies for this question.

Second, the identification of disorder-specific effects might be confounded by high levels of comorbidity across disorders—here, in about one third of the included studies (Table S9), reflecting the high comorbidity levels in MDD, ANX, and CP patients. In control analyses, we found no significant influence of comorbidity on our results. However, confounding effects cannot be ruled out completely, since about 15% of studies did not report comorbidity information (Table S9) and, for example, MDD and ANX may be present in CP patients in only subtle or ‘forme fruste’ fashion and therefore not reported by studies.

Third, in DSM-5 as opposed to previous DSM versions, PTSD is no longer included under ANX due to (among others) etiological considerations [63]. We included PTSD within ANX because of the large phenomenological/symptomatic overlap (for example hypersensitivity to perceived threats), which might rely on shared neuro-behavioral mechanisms [63]. Nevertheless, we conducted a control analysis by excluding all PTSD-studies (Supplementary Methods). Results did not significantly differ from original results (χ²-tests; GMV: *p* ≥ 0.85, iFC: *p* ≥ 0.82), suggesting no significant bias from PTSD-studies.

Fourth, there were specific tendencies of seed distribution in each disorder: for MDD, most seeds were located in DMN, for ANX in LIM, and for CP in SAL (Table S11). Since results were first synthesized within each disorder and only subsequently contrasted across disorders, we controlled for differences in number of studies per network. However, false disorder-specificity due to heterogeneous network-coverage cannot be fully excluded.

Fifth, due to our large-scale approach, our result of common GMV-decreases might miss subtle differences, e.g., regarding insula or ACC subregions [64].

Sixth, we controlled for medication in control analyses. However, as patients might have taken different groups of drugs (e.g., antidepressants or anxiolytics), which is not consistently reported by studies, some uncontrollable influences might remain.

Seventh, we restricted our approach to seed-based iFC, because more ‘complex’/data-driven iFC-measures, like ICA-components or graph-analysis measures, cannot be synthesized easily by coordinate-based meta-analysis. This means that all included iFC-studies had conducted hypothesis-driven analyses; seed selection of input studies could in theory bias our results, since some brain regions might be overrepresented as seeds. Yet, we think that our approach investigates whole-brain network-iFC, because we pooled together all seeds within a given intrinsic network, yielding a representative map of iFC group differences of a particular network, and not just of individual seeds. Furthermore, input studies employed seeds covering wide areas of cortex, striatum, and cerebellum, and performed *whole-brain* seed-to-voxel iFC-analyses – therefore, no brain regions were selected or excluded a priori as iFC-targets [11, 38]. Future studies should additionally consider other measures like dynamic iFC or structural connectivity [65].

Eighth, we grouped seed-regions based on network-assignment rather than based on anatomical location. This means that possibly quite distinct regions (concerning anatomy or function) were grouped together under the assumption of similar iFC-profiles (e.g., medial PFC and precuneus, although anatomically and functionally distinct, were both grouped in the DMN).

Ninth, the included disorders are quite heterogeneous, e.g., MDD manifests itself in diverse symptoms, and CP encompasses different conditions like chronic back pain or fibromyalgia (for details on included conditions, see Supplementary Methods). This issue, which is inherent in most psychiatric diagnostic categories, might reduce power and increase the risk of false-negative results.

Tenth, study quality has generally increased over the last two decades (e.g., regarding normalization accuracy or noise correction), so older studies might confound results. We included only very few studies from before 2010. Possible disproportionate influences from these studies were ruled out by jackknife analyses, which tested for the effects of individual studies on the results. The paucity of pre-2010 studies made other post-hoc analyses impossible. Even after 2010, potential changes in usage of noise correction methods might confound results; however, we observed no clear trends in usage of specific methods (Table S21).

Eleventh, life stress as shared risk factor, as well as other unmeasured confounds, might constitute a confounding factor contributing to common brain changes. As it is hard to quantify, it is typically unmeasured; therefore, a control analysis was not possible.

Twelfth, meta-analyses for iFC-networks were conducted if at least three studies per disorder were present, based on previous work [11, 38]. A low number of studies might potentially compromise statistical power. However, for networks with significant disorder-specific results, mostly more than 20 studies were present per disorder, ensuring sufficient power [66].

Thirteenth, a wide range of phenotypically distinct anxiety disorders were included within the ANX group. We conducted pairwise post-hoc control analyses (for methodology, see other post-hoc analyses above) comparing results in generalized anxiety disorder, panic disorder, and social anxiety disorder (for power reasons, no other sub-groups could be formed; also, analyses had to be restricted to GMV). No significant difference between sub-groups was found (*p* ≥ 0.16).

### Conclusion

We found *common* reductions of gray matter volume in insula and medial PFC across MDD, ANX, and CP, suggesting a shared neural correlate for comorbidity and possibly neuro-behavioral chronification mechanisms. *Disorder-specific* changes across distinct brain networks might underlie distinct phenotypes and possibly additional disorder-specific mechanisms.

## Supplementary information

Supplementary Material
