# Supplementary material for: Common and specific large-scale brain changes in major depressive disorder, anxiety disorders, and chronic pain: a transdiagnostic multimodal meta-analysis of structural and functional MRI studies
Source: Neuropsychopharmacology. 2022 Jan 20;47(5):1071–80. doi: 10.1038/s41386-022-01271-y (PMC8938548; doi:10.1038/s41386-022-01271-y)
Supplement: Supplementary file 1 — Supplementary Material [file 41386_2022_1271_MOESM1_ESM.pdf]

Supplementary Material for Brandl, Weise et al.:

Common and specific large-scale brain changes in major depressive disorder, anxiety disorders, and chronic pain

A transdiagnostic multimodal meta-analysis of structural and functional MRI studies

Contents:

Supplementary Methods **4**
1. Details of literature search 4
2. Details of data extraction 5
3. Details of meta-analysis 6
4. Details of conjunction analyses 8
5. Details of control analyses 10
6. Control analysis for studies without significant results 11

Supplementary Results **13**
1. GMV-changes 13
1.1 GMV-changes in MDD, ANX, and CP compared to healthy controls *13*
1.2 Pairwise contrasts of GMV-changes *13*
2. iFC-changes 14
2.1 iFC-changes in MDD, ANX, and CP compared to healthy controls *14*
2.2 Pairwise contrasts of iFC-changes *15*
3. Control analysis: conjunctions of GMV-changes across pairs of disorders 17

Supplementary Discussion **18**
1. Major depressive disorder 18
1.1 GMV-changes compared to healthy controls *18*
1.2 iFC-changes compared to healthy controls *18*
2. Anxiety disorders 18
2.1 GMV-changes compared to healthy controls *18*
2.2 iFC-changes compared to healthy controls *18*
3. Chronic pain 19
3.1 GMV-changes compared to healthy controls *19*

3.2 iFC-changes compared to healthy controls 19

Supplementary Figures 20

Figure S1: Intrinsic brain networks used in the analyses. 20

Figure S2: Gray matter volume changes compared to healthy controls. 21

Figure S3: Pairwise contrasts of gray matter volume changes. 22

Figure S4: Intrinsic functional connectivity changes compared to healthy controls. 23

Figure S5: Pairwise contrasts of intrinsic functional connectivity changes.......................... 24

Figure S6: Spatial overlap between GMV-increase “MDD vs. HC” and “ANX vs. HC”.......... 25

Figure S7: Spatial overlap between GMV-decrease “MDD vs. HC” and “ANX vs. HC”......... 26

Figure S8: Spatial overlap between GMV-decrease “MDD vs. HC” and “CP vs. HC”. 27

Figure S9: Spatial overlap between GMV-decrease “ANX vs. HC” and “CP vs. HC”............. 28

Figure S10: Spatial overlap between LIM-hypoconnectivity “MDD vs. HC” and “ANX vs. HC”. 29

Figure S11: Spatial overlap between LIM-hypoconnectivity “ANX vs. HC” and “CP vs. HC”. 30

Figure S12: Regional overlap between specific GMV-changes and specific iFC-changes.... 31

Figure S13: Control analysis for studies with no significant results. 32

Supplementary Tables 33

Table S1: Search criteria and included studies. 33

Table S2: MOOSE checklist for meta-analyses of observational studies. 34

Table S3: Demographic and clinical characteristics of major depressive disorder studies included in the GMV meta-analysis. 36

Table S4: Demographic and clinical characteristics of anxiety disorder studies included in the GMV meta-analysis. 40

Table S5: Demographic and clinical characteristics of chronic pain studies included in the GMV meta-analysis. 42

Table S6: Demographic and clinical characteristics of major depressive disorder studies included in the iFC meta-analysis. 47

Table S7: Demographic and clinical characteristics of anxiety disorder studies included in the iFC meta-analysis. 51

Table S8: Demographic and clinical characteristics of chronic pain studies included in the iFC meta-analysis. 54

Table S9: Comorbidity across investigated disorders in the included studies. 57

Table S10: Studies with no significant results. 58

Table S11: Number of studies, number of patients/controls, and mean age of patients/controls per seed network and disorder. 59

Table S12: Gray matter volume changes compared to healthy controls............................ 60

Table S13: Common gray matter volume changes.. 62
Table S14: Pairwise contrasts of gray matter volume changes.. 63
Table S15: Specific gray matter volume changes. 65
Table S16: Intrinsic functional connectivity changes compared to healthy controls. 66
Table S17: Common intrinsic functional connectivity changes.. 68
Table S18: Pairwise contrasts of intrinsic functional connectivity changes........................ 69
Table S19: Specific intrinsic functional connectivity changes. 71
Table S20: Regional overlap between specific GMV-changes and specific iFC-changes. 72
Table S21: Noise correction methods (additional to global signal regression) implemented in iFC studies. 73
Supplementary References 78

Supplementary Methods

1. Details of literature search

The meta-analysis was registered at PROSPERO (registration number: CRD42019119709; https://www.crd.york.ac.uk/PROSPERO/display_record.php?RecordID=119709). Studies were searched until January 01, 2020 in PubMed, Web of Science, EMBASE, and reference lists of reviews and eligible articles, using the keywords *(rest* OR intrinsic) AND connect* AND seed* AND [disorder name]* for the intrinsic functional connectivity (iFC) meta-analysis and *("VBM" OR "voxel-based morphometry") AND [disorder name]* for the gray matter volume (GMV) meta-analysis. Detailed search terms (i.e., disorder name) for each disorder are given in **Table S1**. The following diseases/conditions were eligible for inclusion:

- *Major depressive disorder (MDD)*: diagnosis of major depressive disorder/major depression (including late-life depression and geriatric depression).
- *Anxiety disorders (ANX)*: generalized anxiety disorder, generalized social anxiety disorder, panic disorder, phobias (e.g., social phobia), post-traumatic stress disorder, social anxiety disorder.
- *Chronic pain (CP):* chronic back pain, fibromyalgia, chronic widespread pain, temporomandibular pain/disorder, osteoarthritis, irritable bowel syndrome, chronic pelvic pain, dysmenorrhea, neuropathic pain, phantom pain, complex regional pain syndrome, rheumatoid arthritis, central pain, migraine, tension type headache, postherpetic neuralgia, post-thoracotomy pain syndrome, chronic pancreatitis, ankylosing spondylitis.

All English-language publications of whole-brain voxel-based morphometry (VBM) and seed-based whole-brain iFC comparing patients with MDD, ANX, or CP to healthy subjects were included. Studies were selected jointly by researchers F.B. and B.W. in a multi-step procedure

in line with MOOSE guidelines for meta-analyses of observational studies (**Figure 1**; MOOSE checklist is provided in **Table S2**) (1). We systematically checked study quality concerning design, demographic/clinical characteristics (e.g., overlap of study samples), dropout rates, and precision of seed and result coordinates. Only studies including patients with explicit diagnosis of the respective disorder (e.g., using DSM-5) were selected. Exclusion criteria were (i) methods other than VBM or seed-based iFC, e.g., graph analysis or independent component analysis, which yield different outcome measures; (ii) no whole-brain analysis; (iii) neurological (other than CP) or severe medical comorbidity (psychiatric comorbidity was no exclusion criterion); (iv) no peak-coordinates reported in standard space. In longitudinal or intervention/challenge studies, only baseline results were considered. Task-fMRI data were not considered since they reflect reactions to specific tasks instead of stable/long-term changes. We made no restrictions concerning age, illness duration, symptom severity, or medication status to ensure maximal study coverage, but we conducted several control analyses later on.

2. Details of data extraction

Peak-coordinates of between-group effects were extracted from included GMV- and iFC-studies (**Tables S3-S8**) and, if necessary, converted to Montreal Neurological Institute (MNI) standard space. IFC peak-coordinates were assigned to 'seed-networks' according to the location of the center-coordinate of their respective seed (which had been used in the included study to calculate seed-based iFC) within a parcellation of brain-networks (2,3). We selected a widely-used parcellation based on iFC-data from 1000 healthy subjects, covering cortex, striatum, and cerebellum. It contained 7 seed-networks: visual (VIS), primary-sensorimotor (PSM), dorsal attention (DAN), salience (SAL), limbic (LIM), frontoparietal

(FPN), default-mode (DMN) (**Figure S1**) (4-6). SAL refers to Yeo's 'ventral-attention network', PSM to Yeo's 'somatomotor network' – they were renamed to better fit the existing literature and reflect their constituent regions. As a general limitation, these neurocognitively motivated network names do not claim to describe an exclusive function of the network, but reflect research conventions and enable links to other papers. No similar network-parcellation was available for thalamus, so studies using only thalamus seeds were excluded (2).

3. Details of meta-analysis

MKDA meta-analysis of both GMV and network-iFC maps was conducted (2,3,7,8). For iFC, meta-analyses were only conducted for networks with at least three studies in each disorder to ensure sufficient power. MKDA-analysis followed our previous work on transdiagnostic multimodal meta-analysis of structural and resting-state fMRI-studies across neuropsychiatric disorders (2) and comprised the following steps: smoothing of peak-coordinates with spherical kernels (radius=15mm), weighting of input studies by sample size and finally averaging over the smoothed, weighted study maps (8). The resulting 'density maps' (one for each meta-analytic contrast; see below for contrast details) indicated for each voxel the weighted proportion of input studies that reported a group-difference peak within 15mm of this voxel (=density statistic). Clusters with significantly aberrant GMV or iFC at $p<0.05$ (FWER-corrected for false-positive results from multiple testing) were then identified using Monte-Carlo simulations (15,000 iterations). Clusters significant both based on density statistic (height-based threshold) and based on cluster size (extent-based threshold) were reported, for these thresholds convey complementary information (2,3). Thus, significant results reflect regional brain clusters in which patient-versus-control

differences regarding GMV and network-iFC were reported by a higher proportion of studies than expected by chance (8). The density statistic of each cluster reflects an effect size, i.e., the higher the density statistic, the more studies reported a peak at this location.

In particular, we tested for *common* (i.e., observed in all three disorders) and *specific* (i.e., more pronounced in one than in the other two disorders) changes in MDD, ANX, and CP.

1. *GMV- and iFC-changes common to MDD, ANX, and CP*, consisting of two sub-analyses:
    a. Consistent GMV-increase/decrease and iFC-hyper/hypoconnectivity of one seed-network (within-network-dysconnectivity if the result-cluster overlapped with the seed-network, between-network-dysconnectivity if it overlapped with another seed-network) in MDD, ANX, and CP, respectively, compared to healthy controls (using MKDA-meta-analysis, $p<0.05$ FWER-corrected).
    b. GMV-increase/decrease and iFC-hyper/hypoconnectivity common across MDD, ANX, and CP was then identified via conjunction analysis (for details, see **Supplementary Methods - Details of conjunction analyses**): briefly, we computed the intersection between meta-analytic result-maps and adjusted the significance threshold to account for potential noise in meta-analytic p-value estimations. This well-established procedure takes the already FWE-corrected p-values from meta-analyses as input and leads to an even stricter significance threshold than in the original meta-analysis, depending on the input p-values and the number of input contrasts. In this case, the significance threshold of this three-way-conjunction was $p<0.0015$; details in **Supplementary Methods - Details of conjunction analyses**) (2,9,10).
2. *GMV- and iFC-changes specific to MDD, ANX, and CP*, consisting of two sub-analyses:

a. Pairwise contrasts, e.g., more pronounced iFC-hyperconnectivity in MDD compared to ANX (i.e., MDD>ANX), calculated via MKDA-meta-analysis and subsequent conjunction (p<0.005; details in **Supplementary Methods - Details of conjunction analyses**) (9).
b. Contrasts between one disorder and the other two disorders, e.g., specific iFC-hyperconnectivity in MDD compared to *both* ANX and CP, calculated via conjunction across pairwise contrasts (p<0.00005; details in **Supplementary Methods - Details of conjunction analyses**) (2,9).

3. *Regional overlap between specific GMV-changes and specific iFC-changes*: to investigate whether specific dysconnectivity was associated with specific changes in underlying brain structure, we followed previous work (2) and conjuncted result-maps of specific iFC-changes with result-maps of specific GMV-changes ($p<3x10^{-9}$ adjusted for four-tailed testing; details in **Supplementary Methods - Details of conjunction analyses**) (2,9).

4. Details of conjunction analyses

We computed the intersection between meta-analysis results by multiplying the meta-analytic p-value maps (e.g., "MDD>HC", “ANX>HC”, and "CP>HC"; HC refers to healthy controls). Subsequently, we determined the voxel-wise significance threshold in a widely-used manner that accounts for potential noise in p-value estimation during individual meta-analyses (9).

In detail, the following conjunction analyses were conducted (for clarity, we only describe analyses for GMV-increase/iFC-hyperconnectivity; procedures for GMV-decrease/iFC-

hypoconnectivity were identical) – the numbering corresponds to the numbering of analyses in the preceding paragraph:

1.b. *Common GMV- and iFC-changes*. First, p-value maps of the meta-analytic contrasts "MDD>HC", "ANX>HC", and "CP>HC" were multiplied. Second, the union of meta-analytic p-values in each voxel was calculated with the following formula (9):

$$U = p_{Meta-analysis\ 1} + p_{Meta-analysis\ 2} - p_{Meta-analysis\ 1} \times p_{Meta-analysis\ 2}$$

As we combined three meta-analytic results, this had to be done in a two-step procedure (9):

$$U_{(MDD>HC)\cap(ANX>HC)} = p_{MDD>HC} + p_{ANX>HC} - p_{MDD>HC} \times p_{ANX>HC}$$

$$U = U_{(MDD>HC)\cap(ANX>HC)} + p_{CP>HC} - U_{(MDD>HC)\cap(ANX>HC)} \times p_{CP>HC}$$

Third, the threshold was adjusted since U statistics might be too conservative (9):

$$P = U + (1 - U) \times \ln(1 - U)$$

With meta-analytic significance thresholds of p=0.05 as inputs to these calculations, this yielded a conjunction significance threshold of P=0.0015.

2.a. *Pairwise contrasts of GMV- and iFC-changes*. When calculating greater hyperconnectivity in MDD than in ANX (i.e., "MDD>ANX"), for example, we first conducted an MKDA meta-analysis for the contrast "(MDD>HC) > (ANX>HC)". Subsequently, we conjuncted the meta-analytic result map with the result map for the contrast "MDD>HC" to restrict pairwise results to 'true' changes in MDD (compared with healthy controls (HC)). Since both result maps used as inputs for the conjunction employed significance thresholds of p=0.05, the procedure specified above resulted in a conjunction significance threshold of P=0.005.

2.b. *Specific GMV- and iFC-changes*. When calculating specific hyperconnectivity in MDD (i.e., "(MDD>ANX) ∩ (MDD>CP)"), for example, the hyperconnectivity map "MDD>ANX" was conjuncted with the hyperconnectivity map "MDD>CP". As the significance thresholds of

these maps were p=0.005 (see above), this gave a conjunction significance threshold of P=0.00005.

3. *Overlap between specific GMV- and iFC-changes.* To investigate whether specific GMV-changes and specific iFC-changes overlapped, we followed previous work (2) and conjuncted the result maps of specific GMV-changes with the result maps of specific iFC-changes (e.g., "(MDD>ANX) ∩ (MDD>CP)"). With input significance thresholds of p=0.00005 (see above), this yielded a conjunction significance level of $P=5x10^{-9}$. As this conjunction combined GMV-increase, GMV-decrease, hyperconnectivity, and hypoconnectivity, we had to account for four-tailed testing (9). Therefore, the conjunction significance threshold was further divided by 2, resulting in $P=3x10^{-9}$.

5. Details of control analyses

Jackknife analyses. We controlled for disproportionate influences of single studies via jackknife analyses: we iteratively left out one study, recalculated the density statistic of each significant cluster of the original analyses (contrasts "disorder vs. HC"), and compared it to the original density statistic (i.e., with all studies) via χ^2-test (2,11).

Post-hoc analyses for comorbidity and further demographic and clinical variables. Following an established approach using sub-group analyses (2,3,10), we grouped studies by comorbidity with one or both of the other two disorders, age (<18 years = teen, 18-35 years = young adult, 35-60 years = middle aged, 60 years = senior), gender (≥50% male subjects vs. <50% male subjects), and use of medication (yes vs. no) (**Table S3**-**Table S8**). For each demographic/clinical variable (e.g., age), the density statistic of each significant cluster was recalculated for each sub-group (e.g., young adult) and then compared to the other sub-groups via χ^2-tests (2,3,10).

Post-hoc analyses for methodological factors. GMV-studies were grouped by whether or not they had employed modulation during voxel-based morphometry (**Table S3**-**Table S5**), and iFC-studies were grouped by whether or not they had used global signal regression during fMRI-analysis (**Table S6**-**Table S8**). For each methodological factor (e.g., modulation), the density statistic of each significant cluster was recalculated separately for each sub-group (e.g., modulation used) and then compared to the other sub-group (e.g., modulation not used) via χ^2-test (2,3,10).

Post-hoc analyses for posttraumatic stress disorder (PTSD)-studies. We excluded all PTSD-studies, recalculated the density statistic of each cluster that had been significant in the original analysis of ANX vs. HC (both GMV and iFC), and compared it to the original density statistic (i.e., with all studies) via χ^2-test (11).

6. Control analysis for studies without significant results

Since such an analysis is not feasible within the MKDA framework, we utilized another widely-used toolbox for coordinate-based meta-analysis, namely "Seed-based d-Mapping with Permutation of Subject Images (SDM-PSI)" (12,13). In brief, this method estimates effect sizes of input peaks and performs a meta-analysis to identify effects which are consistent across studies. Significant clusters are then detected via permutation tests (providing family-wise error correction for multiple comparisons) and subsequent threshold-free cluster enhancement (TFCE) statistics. Crucially, the estimation of effect sizes is achieved by MetaNSUE algorithms which allow for the inclusion of studies that did not report any significant peaks.

This control analysis was conducted only for GMV – for iFC, there were maximally 2 non-significant studies per disorder, and apart from one study, all employed seeds that were

located in seed-networks without significant meta-analytic results. A detailed list of studies with no significant results can be found in **Table S10**. The results of this control analysis were visually compared to the original results (i.e., without non-significant studies) and are shown in **Figure S13**.

Supplementary Results

1. GMV-changes

1.1 GMV-changes in MDD, ANX, and CP compared to healthy controls

Major depressive disorder. Gray matter volume was increased in the right lateral occipital cortex, mostly located within the default-mode network (DMN) and to a lesser degree within visual (VIS) and dorsal attention network (DAN) (**Figure S2, Table S12**). Decreased GMV was found in the medial and lateral prefrontal cortex, insula, superior temporal gyrus, hippocampus, and striatum. GMV-decreases overlapped with several intrinsic brain networks, but mostly with DMN.

Anxiety disorders. Increased gray matter volume was found in the right occipital cortex, located mainly within DMN (**Figure S2, Table S12**). GMV was decreased in bilateral hypothalamus, thalamus, striatum (putamen, Ncl. accumbens), and insula (left: whole, right: posterior), right centromedial amygdala, and left hippocampus (anterior and posterior), parahippocampal gyrus, temporal pole, and ventrolateral prefrontal cortex. GMV-decreases lay mostly within the DMN (and to a lesser degree within other networks).

Chronic pain. Increased GMV was found for bilateral hippocampus (left: whole, right: posterior), parahippocampal gyrus, and Ncl. accumbens, left amygdala (basolateral and centromedial) and putamen, and right cerebellar hemisphere (**Figure S2, Table S12**). GMV-increase was located mostly in DMN and limbic network (LIM). GMV-decrease was observed for right anterior insula, prefrontal cortex, paracingulate gyrus, and frontal pole. GMV-decrease was located mostly in DMN and primary-sensorimotor network (PSM).

1.2 Pairwise contrasts of GMV-changes

Major depressive disorder. We identified significantly more pronounced GMV-decrease in MDD compared with CP for right parahippocampal gyrus and left superior temporal gyrus (**Figure S3, Table S14**). These clusters were located mostly within DAN.

Compared with ANX, distinct GMV-decrease in MDD was found for left premotor cortex, overlapping mainly with LIM, DMN, and frontoparietal network (FPN).

Anxiety disorders. Significantly more pronounced GMV-decrease in ANX than in CP was found for bilateral temporal pole and right parahippocampal gyrus, overlapping predominantly with DMN (**Figure S3, Table S14**).

Pairwise GMV-decrease in ANX compared with MDD was observed for left inferior frontal gyrus, left thalamus, right parahippocampal gyrus, right temporal pole and right amygdala. GMV-decrease was located mainly in DMN, PSM, and SAL.

Chronic pain. Distinct GMV-decrease in CP compared to MDD was identified for frontal pole, prefrontal cortex, right amygdala and paracingulate gyrus (overlapping mainly with DMN), and distinct GMV-increase for left insula, right putamen, right thalamus, right parahippocampal gyrus and cerebellar hemisphere (located mostly in LIM and DMN) (**Figure S3, Table S14**).

Compared to ANX, pairwise GMV-decrease in CP was shown for right insula (overlapping mostly with the salience network (SAL)), as well as GMV-increase in bilateral amygdala, right thalamus and right parahippocampal gyrus (predominantly located in LIM and DMN).

2. iFC-changes

2.1 iFC-changes in MDD, ANX, and CP compared to healthy controls

Major depressive disorder. Regarding PSM, iFC with right lateral occipital cortex was enhanced (**Figure S4, Table S16**). Concerning LIM, connectivity with right ventral putamen, amygdala, and anterior insula was decreased. FPN showed decreased iFC with right angular

gyrus. For DMN, we found increased connectivity with left precuneus and left dorsolateral prefrontal cortex. In summary, we found mainly hypoconnectivity within FPN, between FPN and DAN and between LIM and SAL, as well as hyperconnectivity between DMN and FPN.

Anxiety disorders. For LIM, iFC with right angular/supramarginal gyrus, right occipital cortex and right cerebellar hemisphere was enhanced, whereas with right posterior cingulate cortex, connectivity was reduced (**Figure S4, Table S16**). In summary, we found both hyper- and hypoconnectivity of LIM (within itself and with other networks).

Chronic pain. Concerning PSM, we found decreased iFC with right posterior insula, putamen, superior temporal gyrus, ventroateral prefrontal cortex, and fusiform gyrus (**Figure S4, Table S16**). SAL showed hypoconnectivity with bilateral dorsomedial prefrontal cortex, anterior and posterior cingulate cortices, precuneus, and occipital cortex, as well as hyperconnectivity with left pre- and postcentral gyrus and precuneus. LIM showed hypoconnectivity with left medial prefrontal cortex. For FPN, connectivity with left superior temporal gyrus was reduced. In summary, we found mainly hypoconnectivity between SAL and DMN and hyperconnectivity between SAL and PSM.

2.2 Pairwise contrasts of iFC-changes

These analyses were conducted for PSM, SAL, LIM, FPN, and DMN, because common GMV-decrease overlapped with these networks and at least three studies per disorder were available for them (**Table S11**).

Major depressive disorder. For MDD compared to CP, distinct (i.e., more pronounced) hyperconnectivity was shown for PSM in right lateral occipital cortex (**Figure S5, Table S18**). For LIM, hypoconnectivity was observed in right ventral putamen, amygdala and right insula, and for FPN in right angular gyrus. DMN showed hyperconnectivity in left dorsolateral

prefrontal cortex. In summary, we found mainly DMN-FPN-hyperconnectivity, within-FPN-hypoconnectivity, LIM-SAL-hypoconnectivity, and FPN-DAN-hypoconnectivity.

Compared to ANX, distinct hypoconnectivity in MDD was shown for LIM in right ventral putamen, amygdala, and anterior insula, while for DMN, hyperconnectivity was identified for left dorsolateral prefrontal cortex and left precuneus. In summary, we found mainly DMN-FPN-hyperconnectivity and LIM-SAL-hypoconnectivity.

Anxiety disorders. We identified significantly more pronounced hyperconnectivity in ANX compared with CP for LIM in right cerebellar hemisphere and right angular/supramarginal gyrus. In summary, we found hyperconnectivity of LIM with several other networks, predominantly with PSM (**Figure S5, Table S18**).

Compared with MDD, distinct hyperconnectivity in ANX was found for LIM in right cerebellar hemisphere and right angular/supramarginal gyrus, and LIM-hypoconnectivity in right posterior cingulate cortex. In summary, we found hyper- and hypoconnectivity of LIM with several other networks, mainly with PSM and DMN.

Chronic pain. Significantly more pronounced hypoconnectivity in CP compared with MDD was found for PSM in right posterior insula, putamen, superior temporal gyrus, and ventrolateral prefrontal cortex (**Figure S5, Table S18**). SAL showed hyperconnectivity in left pre- and postcentral gyrus and hypoconnectivity in bilateral dorsomedial prefrontal cortex, anterior and posterior cingulate cortices, and precuneus. For LIM, hypoconnectivity was observed in left medial prefrontal cortex and for FPN in left superior temporal gyrus. In summary, we found mainly SAL-DMN-hypoconnectivity and SAL-PSM-hyperconnectivity.

Compared to ANX, pairwise hypoconnectivity was observed for PSM in right posterior insula, putamen, superior temporal gyrus, ventrolateral prefrontal cortex, and fusiform gyrus, and

for SAL in bilateral dorsomedial prefrontal cortex, anterior and posterior cingulate cortices, and precuneus. In summary, we observed mainly SAL-DMN-hypoconnectivity.

3. Control analysis: conjunctions of GMV-changes across pairs of disorders

Concerning GMV-increase, we found spatial overlap only between MDD and ANX, which focused on the right parieto-occipital transition (**Figure S6**). For the pairs MDD and CP as well as ANX and CP, there were no overlaps.

Concerning GMV-decrease, pairwise results were similar, although larger, to the three-disorder conjunction results reported in the main text. All pairwise results showed rather similar GMV-decrease in left insula and bilateral medial PFC and ACC. Interestingly, in the pair "MDD and ANX", GMV-decreases in bilateral hippocampus and amygdala were differentially more pronounced (**Figure S7**), in the pair "MDD and CP" in the left ventral striatum (**Figure S8**), and in the pair "ANX and CP" in the right insula (**Figure S9**).

Supplementary Discussion

1. Major depressive disorder

1.1 GMV-changes compared to healthy controls

Via MKDA meta-analysis, we detected consistent GMV-decrease in medial and lateral prefrontal cortex, insula, superior temporal gyrus, hippocampus, and striatum, as well as GMV-increase in lateral occipital cortex (**Figure S2**). These findings are well in line with previous meta-analyses of GMV-changes in MDD compared to healthy subjects (14-16).

1.2 iFC-changes compared to healthy controls

We found mainly within-FPN-hypoconnectivity, LIM-SAL-hypoconnectivity, and DMN-FPN-hyperconnectivity (**Figure S4**). These findings are well compatible with a previous meta-analysis of aberrant network-iFC in MDD (3).

2. Anxiety disorders

2.1 GMV-changes compared to healthy controls

We identified rather localized GMV-increase in occipital cortex and more widespread GMV-decrease in prefrontal, cingulate, insular cortices, and subcortical regions such as striatum, amygdala, hypothalamus, thalamus, and hippocampus (**Figure S2**). These findings are compatible with results from previous meta-analyses of GMV in anxiety disorders (17-19).

2.2 iFC-changes compared to healthy controls

We detected both hyper- and hypoconnectivity focused on LIM (within itself and with other networks) (**Figure S4**). LIM-DMN- and LIM-FPN-hypoconnectivity, for example, are in line with a recent meta-analysis of network-iFC in ANX (7).

3. Chronic pain

3.1 GMV-changes compared to healthy controls

GMV-increase was detected in hippocampus, amygdala, striatum, and cerebellum, whereas GMV-decrease focused on prefrontal, cingulate, and insular cortices (**Figure S2**). These results are in line with findings of previous meta-analyses of GMV comparing patients with CP to healthy subjects (20-22).

3.2 iFC-changes compared to healthy controls

We found mainly hypoconnectivity between SAL and DMN and hyperconnectivity between SAL and PSM (**Figure S4**). To our knowledge, this is the first meta-analysis of network-iFC in CP, so there are no previous results to compare our findings to. However, the identified networks and regions are in line with previous literature reviews of CP, which highlighted aberrant iFC of DMN, SAL, and PSM (23-25).

Supplementary Figures

Figure S1: Intrinsic brain networks used in the analyses.

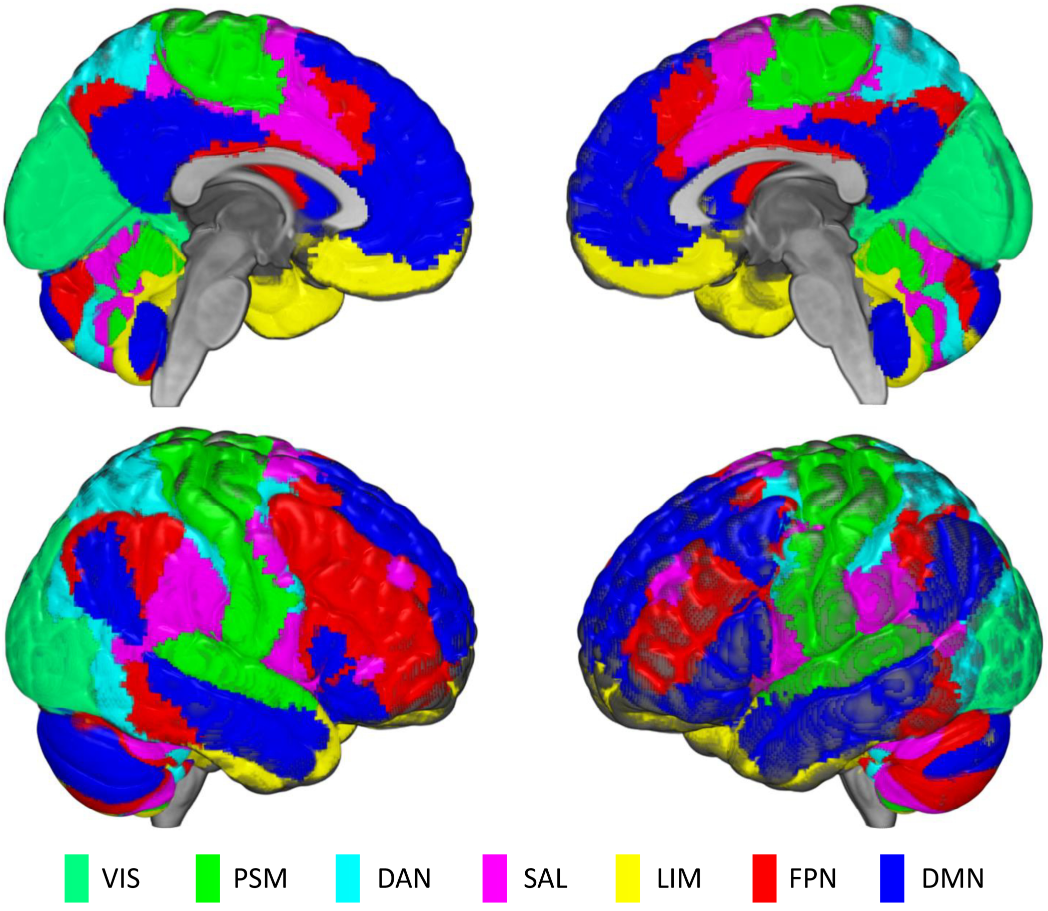

Figure S1: Intrinsic brain networks derived from a clustering approach on iFC-data of 1000 healthy subjects, covering cortex, striatum, and cerebellum (4-6). DAN = dorsal attention network, DMN = default-mode network, FPN = frontoparietal network, LIM = limbic network, PSM = primary-sensorimotor network, SAL = salience network, VIS = visual network.

Figure S2: Gray matter volume changes compared to healthy controls.

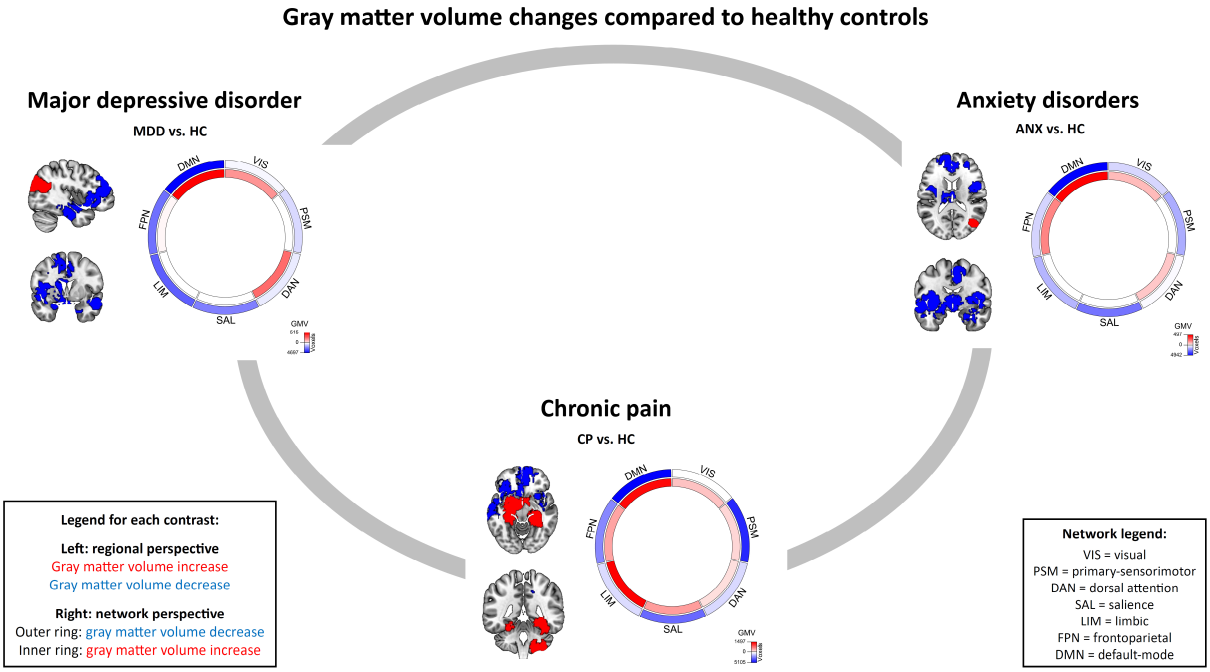

Figure S2: Gray matter volume changes were calculated by MKDA meta-analysis ($p<0.05$ FWER-corrected, height-based and extent-based threshold combined). For each contrast, meta-analytic regional result clusters are shown on the left. Their overlap with intrinsic brain networks (4-6) is displayed on the right: GMV-decrease is shown in the outer ring, GMV-increase in the inner ring of each diagram; color intensity reflects the size of spatial overlap (the more voxels, the stronger the color – a colorscale is added to each plot). ANX = anxiety disorder, CP = chronic pain, HC = healthy controls, MDD = major depressive disorder.

Figure S3: Pairwise contrasts of gray matter volume changes.

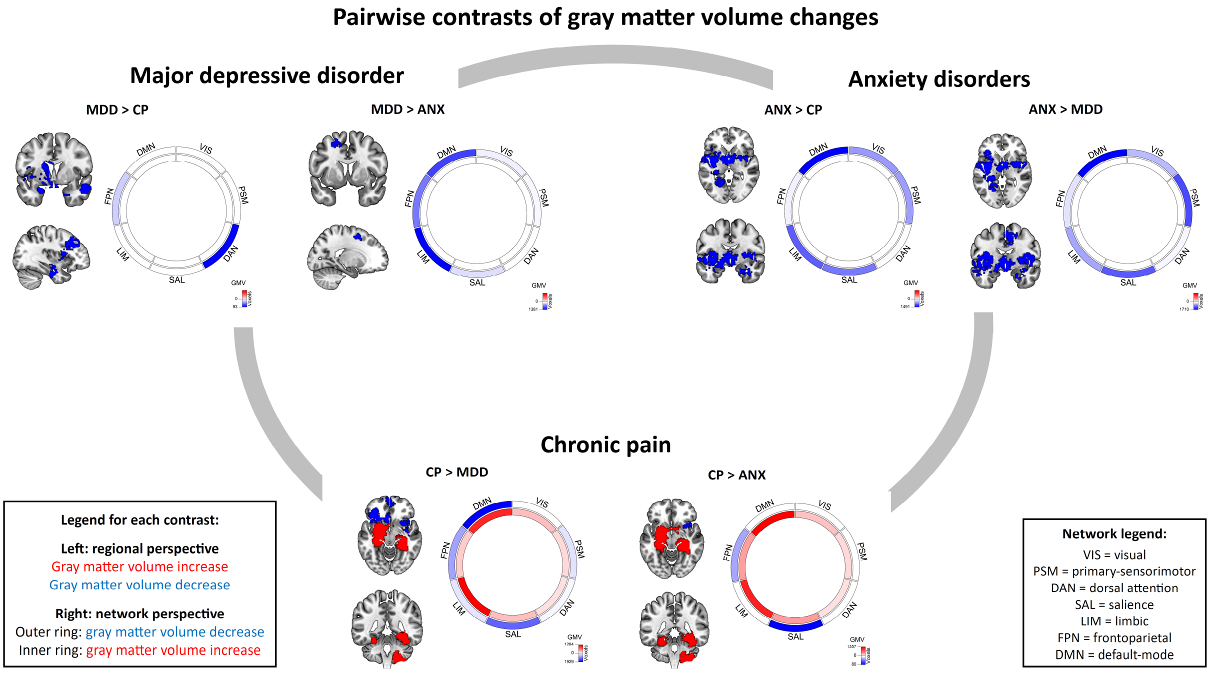

Figure S3: Pairwise contrasts of gray matter volume changes were calculated via MKDA meta-analytic contrasts between disorders (e.g., “MDD>ANX”) and subsequent conjunction with the contrast vs. healthy controls (e.g., “MDD>HC”) to restrict pairwise results to ‘true’ changes in each disorder compared with healthy subjects (p<0.005). For each contrast, meta-analytic regional result clusters are shown on the left. Their overlap with intrinsic brain networks (4-6) is displayed on the right: GMV-decrease is shown in the outer ring, GMV-increase in the inner ring of each diagram; color intensity reflects the size of spatial overlap (the more voxels, the stronger the color – a colorscale is added to each plot). ANX = anxiety disorder, CP = chronic pain, HC = healthy controls, MDD = major depressive disorder.

Figure S4: Intrinsic functional connectivity changes compared to healthy controls.

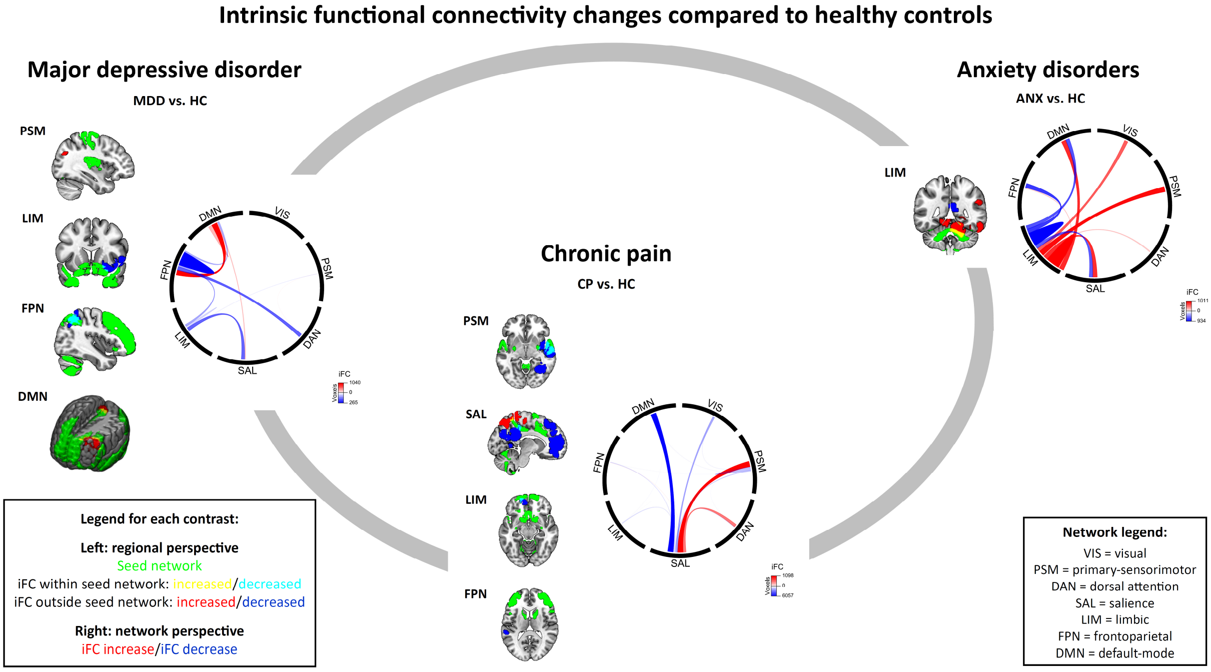

Figure S4: Intrinsic functional connectivity changes were calculated by MKDA meta-analysis (p<0.05 FWER-corrected, height-based and extent-based threshold combined). For each contrast, meta-analytic regional result clusters are shown on the left. Their overlap with intrinsic brain networks (4-6) is displayed on the right in a “chord diagram” (26): between-network connectivity is shown as links, within-network connectivity as “hills”; both color intensity and link thickness reflect the size of spatial overlap (the more voxels, the stronger the color and the thicker the link – a colorscale is added to each plot). ANX = anxiety disorder, CP = chronic pain, HC = healthy controls, iFC = intrinsic functional connectivity, MDD = major depressive disorder.

Figure S5: Pairwise contrasts of intrinsic functional connectivity changes.

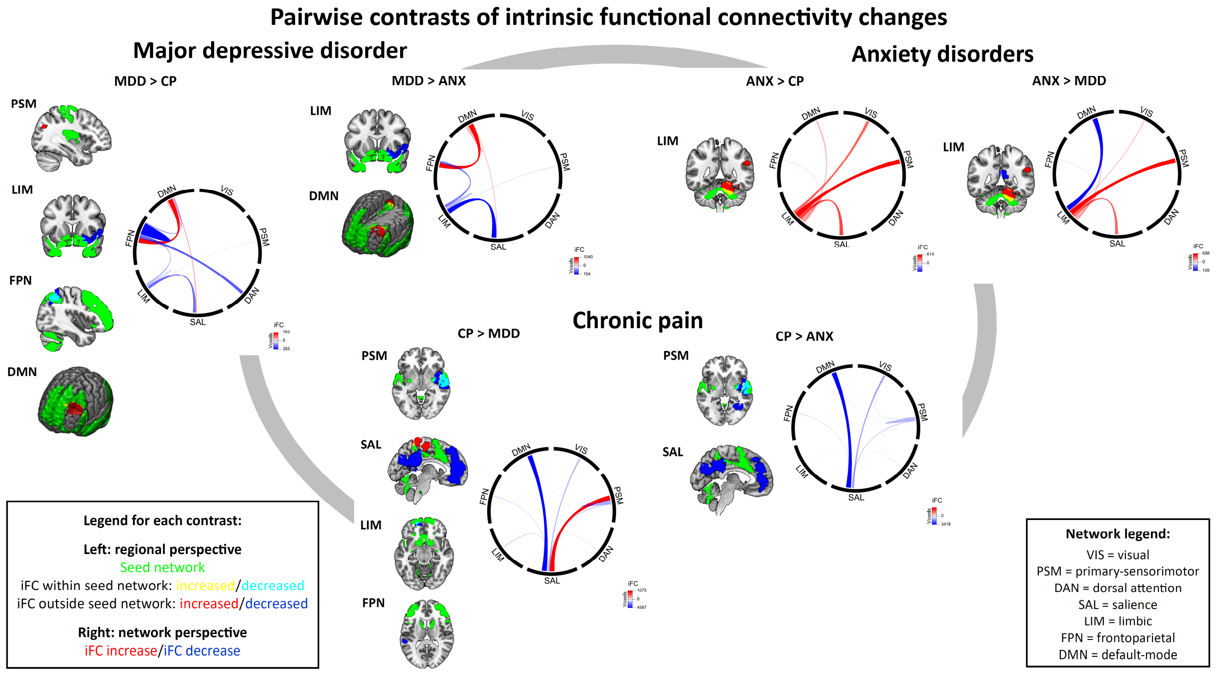

Figure S5: Pairwise contrasts of intrinsic functional connectivity changes were calculated via MKDA meta-analytic contrasts between disorders (e.g., “MDD>ANX”) and subsequent conjunction with the contrast vs. healthy controls (e.g., “MDD>HC”) to restrict pairwise results to ‘true’ changes in each disorder compared with healthy subjects ($p<0.005$). For each contrast, meta-analytic regional result clusters are shown on the left. Their overlap with intrinsic brain networks (4-6) is displayed on the right in a “chord diagram” (26): between-network connectivity is shown as links, within-network connectivity as “hills”; both color intensity and link thickness reflect the size of spatial overlap (the more voxels, the stronger the color and the thicker the link – a colorscale is added to each plot). ANX = anxiety disorder, CP = chronic pain, HC = healthy controls, iFC = intrinsic functional connectivity, MDD = major depressive disorder.

Figure S6: Spatial overlap between GMV-increase "MDD vs. HC" and "ANX vs. HC".

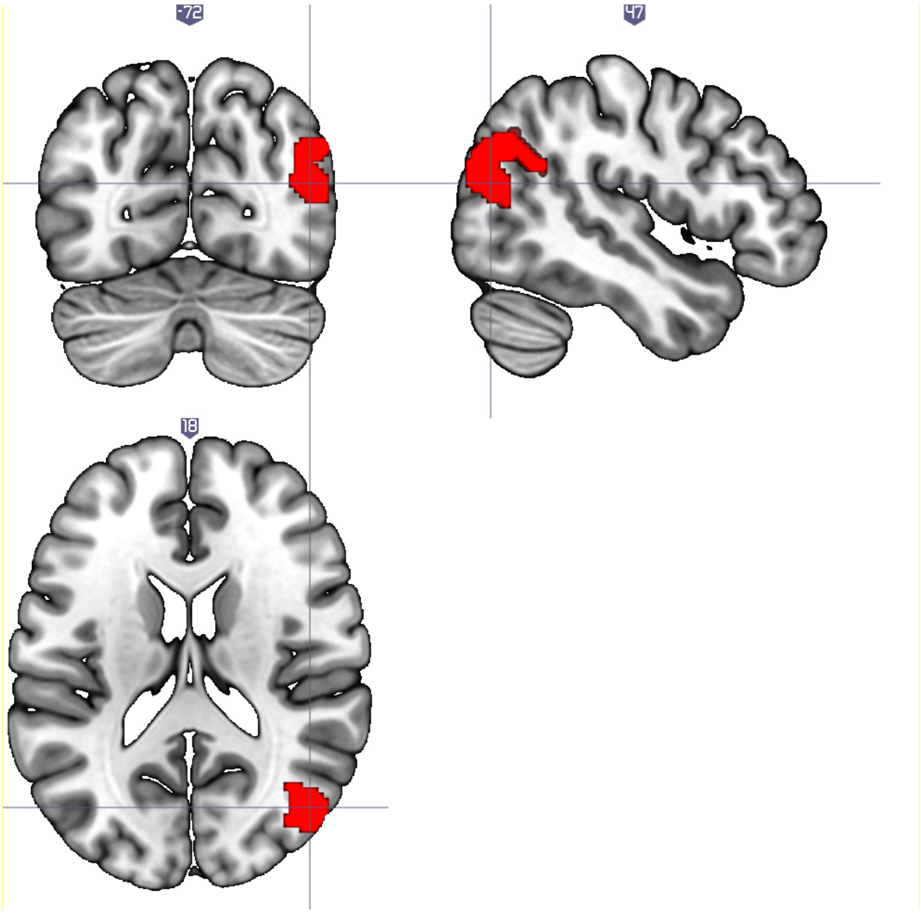

Figure S7: Spatial overlap between GMV-decrease "MDD vs. HC" and "ANX vs. HC".

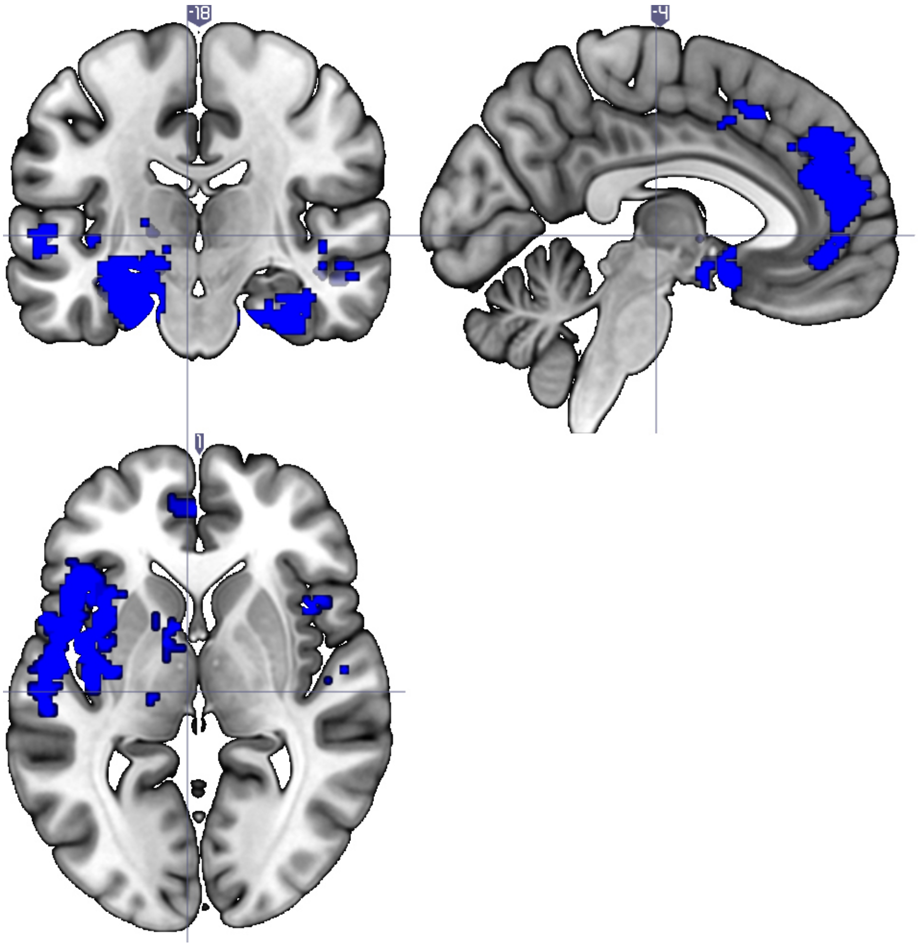

Figure S8: Spatial overlap between GMV-decrease "MDD vs. HC" and "CP vs. HC".

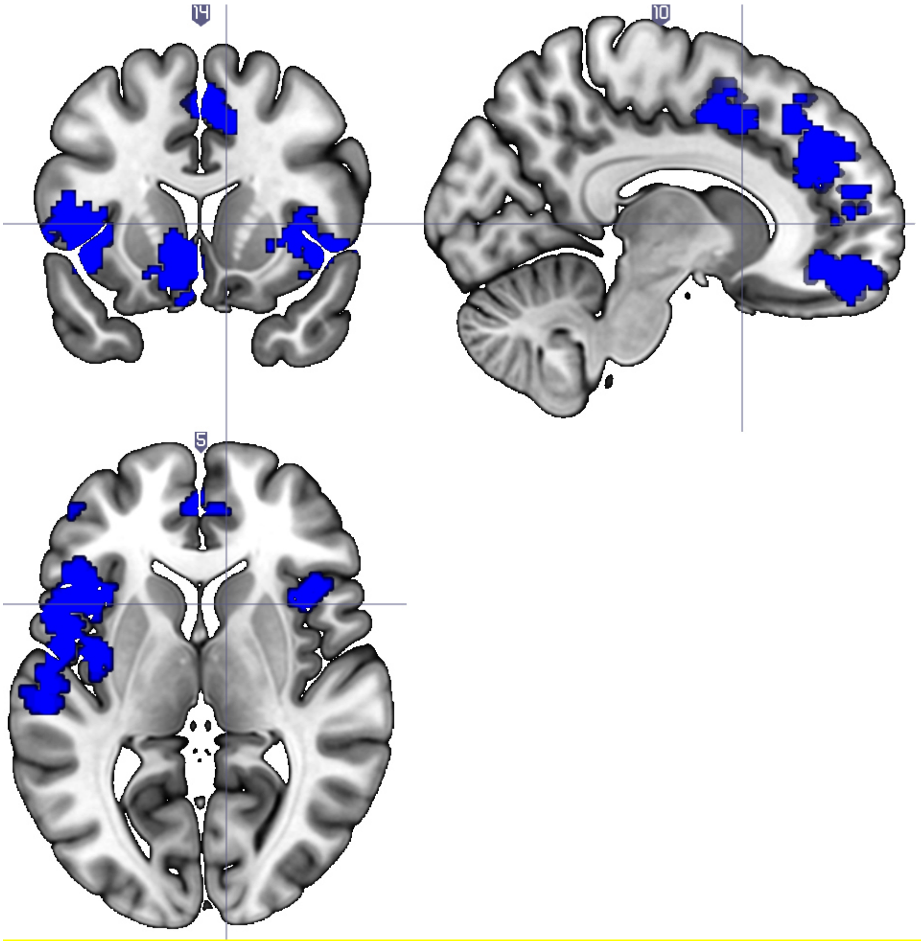

Figure S9: Spatial overlap between GMV-decrease "ANX vs. HC" and "CP vs. HC".

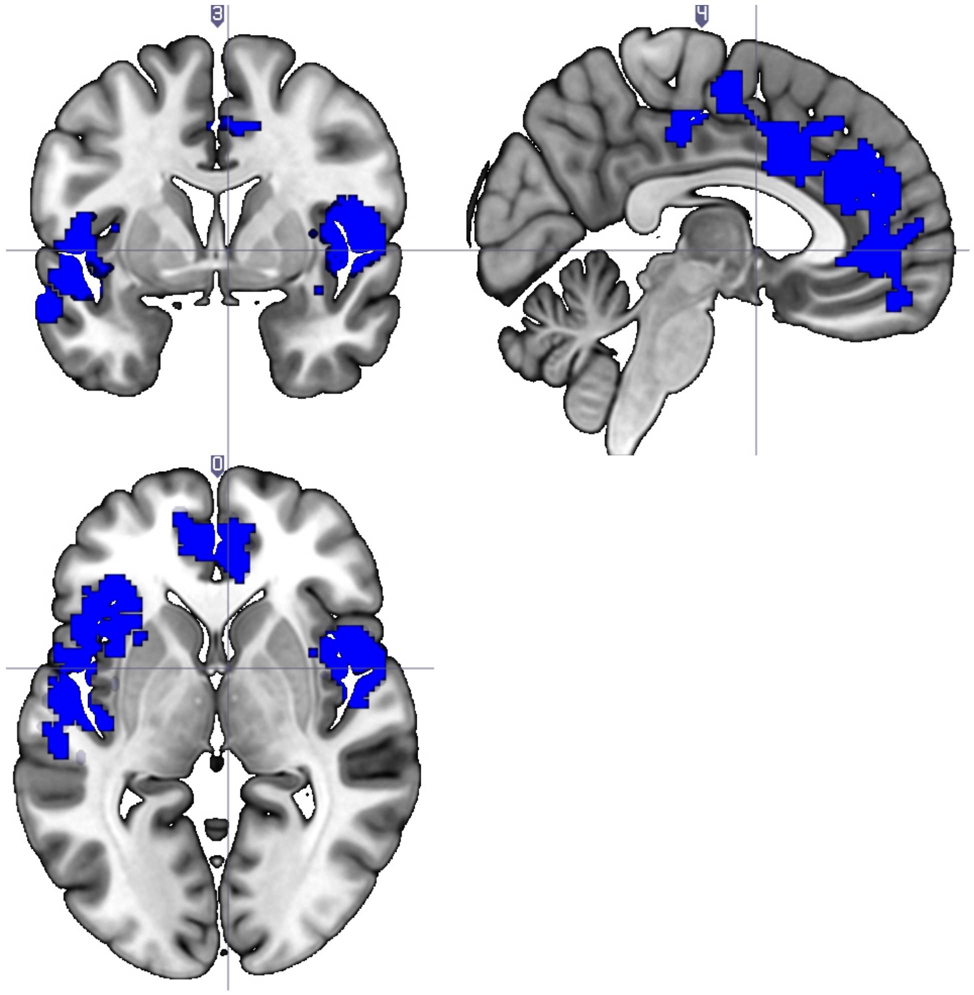

Figure S10: Spatial overlap between LIM-hypoconnectivity "MDD vs. HC" and "ANX vs. HC".

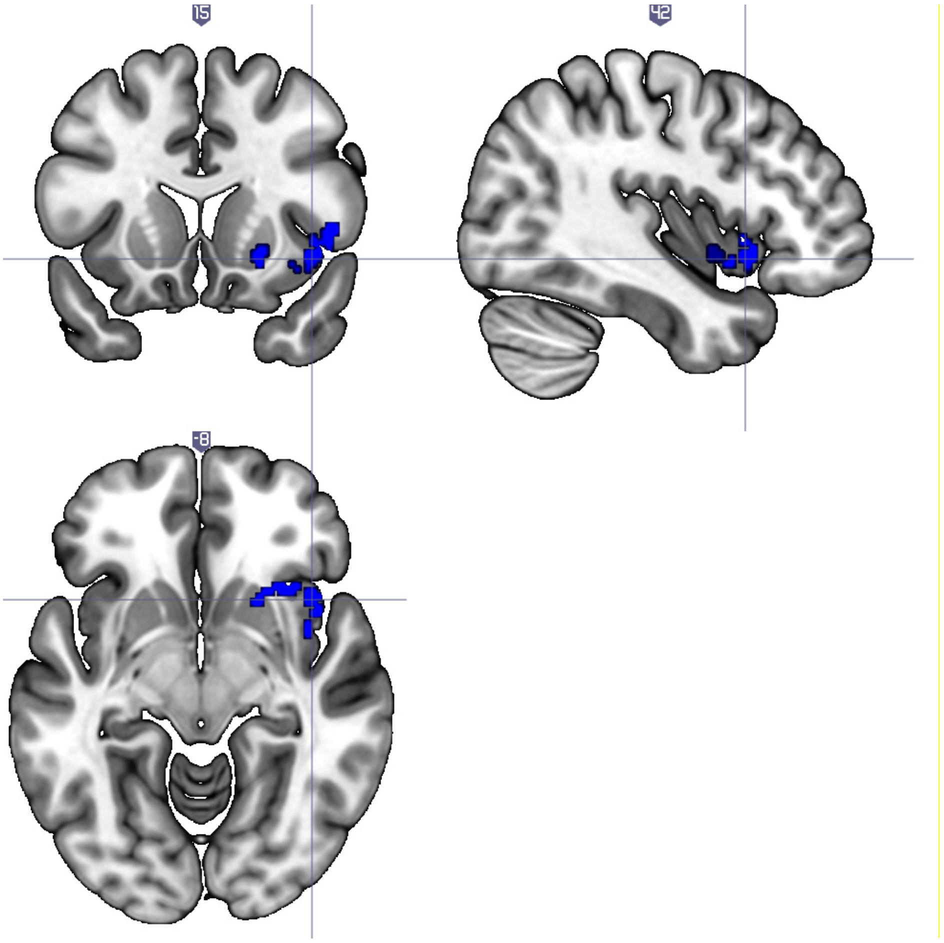

Figure S11: Spatial overlap between LIM-hypoconnectivity "ANX vs. HC" and "CP vs. HC".

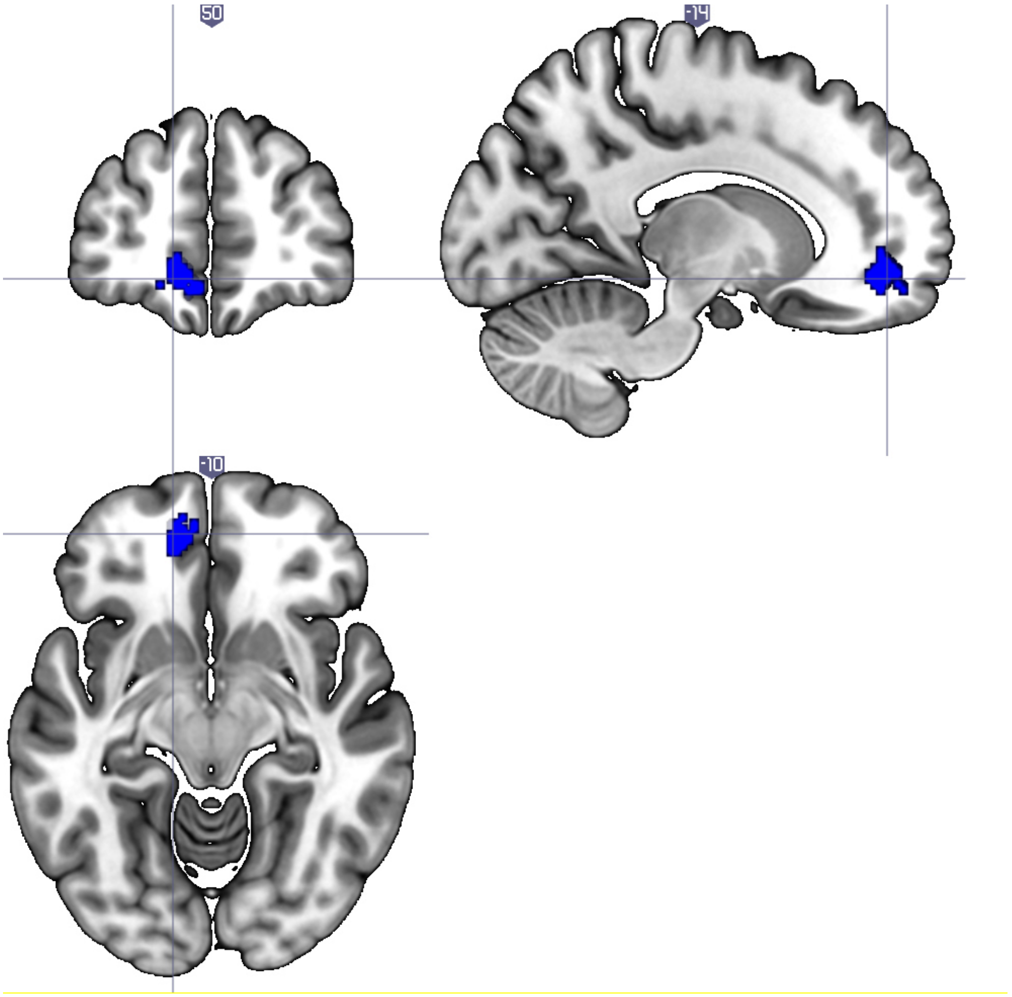

Figure S12: Regional overlap between specific GMV-changes and specific iFC-changes.

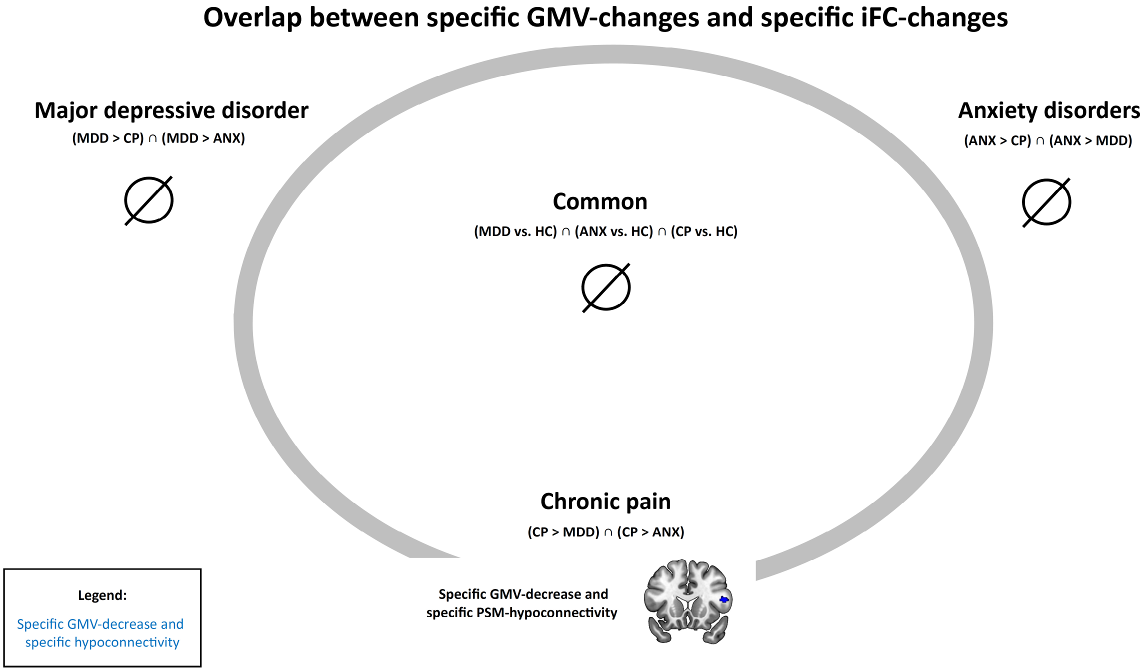

Figure S12: Overlap between result maps for specific GMV-changes (see **Figure 2** in the main text) and specific iFC-changes (see **Figure 3** in the main text) calculated by inter-modality conjunction analysis ($p<3x10^{-9}$).

Figure S13: Control analysis for studies with no significant results.

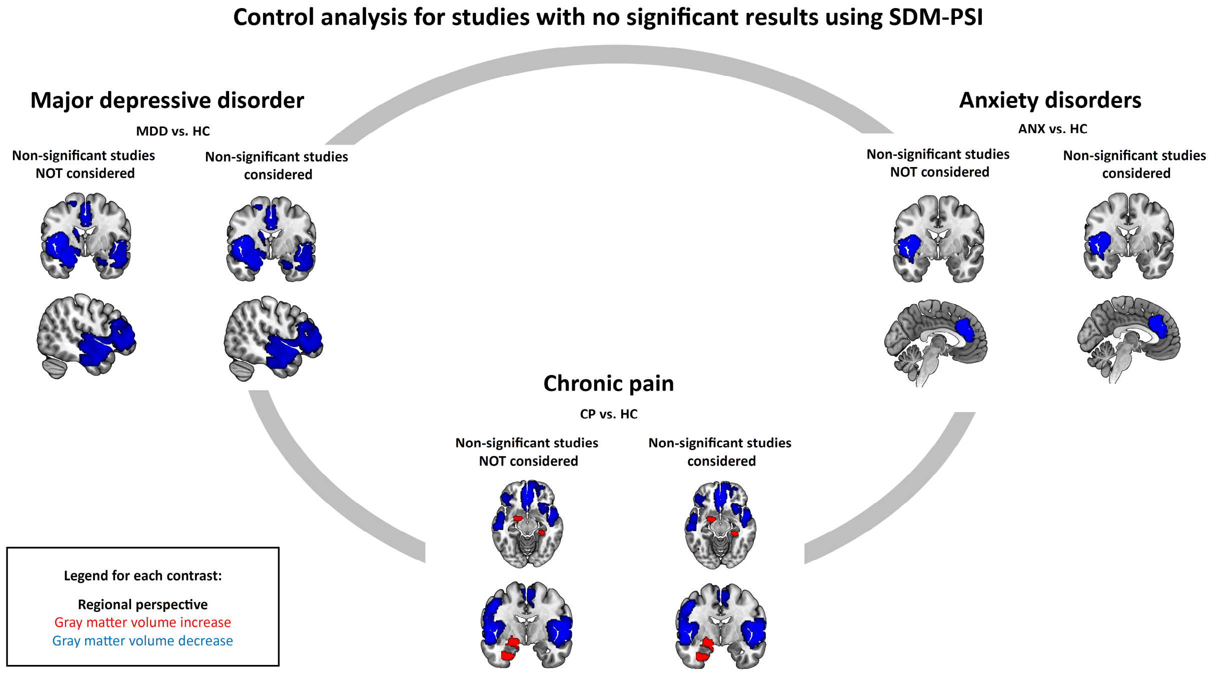

Figure S13: Results were calculated via "Seed-based d-Mapping with Permutation of Subject Images (SDM-PSI)" (12,13) and are significant at $p<0.05$ FWER-corrected (TFCE-based). On the left of each panel, we present results for the same study sample as in the main analysis to show the good comparability between MKDA and SDM-PSI toolboxes. On the right of each panel, we show the results additionally including all studies with no significant peak effects (**Table S10**), which are virtually identical to the results without these studies.

Supplementary Tables

Table S1: Search criteria and included studies.

Disorder	Search terms	published until	Included studies (n)	Controls (n)	Patients (n)
**Gray matter volume meta-analysis**					
**Major depressive disorder**	("VBM" OR "voxel-based morphometry") AND depress*	July 01, 2019	63	3284	2934
**Anxiety disorders**	("VBM" OR "voxel-based morphometry") AND (anxiety disorder OR phobi* OR social anxiety OR posttraumatic OR panic)	July 01, 2019	41	1130	1021
**Chronic pain**	("VBM" OR "voxel-based morphometry") AND pain	July 01, 2019	65	2358	2185
Total			*169*	*6772*	*6140*
**Intrinsic functional connectivity meta-analysis**					
**Major depressive disorder**	(rest* OR intrinsic) AND connect* AND seed* AND depress*	July 01, 2019	68	2141	2314
**Anxiety disorders**	(rest* OR intrinsic) AND connect* AND seed* AND (anxiety disorder OR phobi* OR social anxiety OR posttraumatic OR panic)	July 01, 2019	41	1108	1248
**Chronic pain**	(rest* OR intrinsic) AND connect* AND seed* AND pain	January 01, 2020	42	1114	1229
Total			*151*	*4363*	*4791*

Table S2: MOOSE checklist for meta-analyses of observational studies.

Reporting criteria	Reported on page No.
Reporting of background should include	
Problem definition	7
Hypothesis statement	8
Description of study outcome(s)	8
Type of exposure or intervention used	8
Type of study designs used	7
Study population	7
Reporting of search strategy should include	
Qualifications of searchers (eg, librarians and investigators)	Supplement
Search strategy, including time period included in the synthesis and key words	9
Effort to include all available studies, including contact with authors	Supplement
Databases and registries searched	9
Search software used, name and version, including special features used (eg, explosion)	9
Use of hand searching (eg, reference lists of obtained articles)	Supplement
List of citations located and those excluded, including justification	Supplement
Method of addressing articles published in languages other than English	9
Method of handling abstracts and unpublished studies	Supplement
Description of any contact with authors	Supplement
Reporting of methods should include	
Description of relevance or appropriateness of studies assembled for assessing the hypothesis to be tested	Supplement
Rationale for the selection and coding of data (eg, sound clinical principles or convenience)	Supplement
Documentation of how data were classified and coded (eg, multiple raters, blinding and interrater reliability)	Supplement
Assessment of confounding (eg, comparability of cases and controls in studies where appropriate)	Supplement
Assessment of study quality, including blinding of quality assessors, stratification or regression on possible predictors of study results	Supplement
Assessment of heterogeneity	Supplement
Description of statistical methods (eg, complete description of fixed or random effects models, justification of whether the chosen models account for predictors of study results, dose-response models, or cumulative meta-analysis) in sufficient detail to be replicated	9-11, Supplement
Provision of appropriate tables and graphics	Figure 1, Table S1
Reporting of results should include	
Graphic summarizing individual study estimates and overall estimate	Figures 2-4
Table giving descriptive information for each study included	Table S3-Table S8
Results of sensitivity testing (eg, subgroup analysis)	16
Indication of statistical uncertainty of findings	Table S12-Table S20
Reporting of discussion should include	
Quantitative assessment of bias (eg, publication bias)	17, 22-24
Justification for exclusion (eg, exclusion of non-English language citations)	22-24
Assessment of quality of included studies	22-24
Reporting of conclusions should include	

Consideration of alternative explanations for observed results	18-19
Generalization of the conclusions (ie, appropriate for the data presented and within the domain of the literature review)	18-19
Guidelines for future research	18-19
Disclosure of funding source	25

Table S2. Based on Stroup et al., 2000 (1).

Table S3: Demographic and clinical characteristics of major depressive disorder studies included in the GMV meta-analysis.

Author, year	HC (n)	Male (%)	Mean age HC [years]	MDD (n)	Male (%)	Mean age MDD [years]	Illness duration	Medication	Modulation during VBM	Comorbidity
Ahn et al., 2016 (27)	26	27	31.4±7.6	34	15	32.5±7.95	-	-	no	no
Amico et al., 2011 (28)<br>without a family history of depression	64	56	30.4±8.3	33	58	32.0±8.0	3.4±5.0 years	yes	yes	no
with a family history of depression	30	57	30.7±8.0	33	58	32.0±8.0	3.4±5.0 years	yes	yes	no
Arnone et al., 2013 (29)	66	30	32.1±9.3	39	27	36.3±8.8	-	no	yes	no
Cai et al., 2015 (30)	23	57	28.2±3.8	23	57	30.0±7.3	4,35±2.38 years	no	yes	no
Chen et al., 2016 (31)	28	50	33±11.7	27	48	33±10.8	79±86 months	no	no	no
Cheng et al., 2010 (32)	68	31	28.94±7.82	68	31	29.91±7.92	10.98±8.2 months	no	yes	no
Depping et al., 2015 (33)	22	0	31.4±11.2	22	0	33.5±8.9	5.5±4.7 years	yes	yes	no
Egger et al., 2008 (34)	20	35	72.3±7.77	14	29	71.4±7.49	-	yes	yes	no
Fang et al., 2015 (35)	18	56	59.1±7.5	20	60	59.2±3.7	3.6±1.1 years	-	no	not mentioned
Frodl et al., 2008 (36)	77	7	40.5±11.6	77	55	42.6±12.4	5.4±8.2 years	yes	yes	no
Grieve et al., 2013 (37)	34	53	31.5±12.4	102	47	33.8±13.1	11.3±11.8 years	-	yes	not mentioned
Guo et al., 2014 (38)	44	45	29.39±6.6	44	50	27.52±8.57	19.61±36.50 months	no	no	no
Harada et al., 2016 (39)	61	28	62.9±7.6	45	42	60.2±8.2	10.6±11 years	yes	no	SAD (n=1), PD (n=1)
Hwang et al., 2010 (40)	26	100	79.5±4.3	70	100	79.4±5.3	-	-	yes	not mentioned
Igata et al., 2017 (41)	44	73	41.2±11.6	27	56	45.8±12.7	-	no	yes	no
Kim et al., 2008 (42)	25	0	35.3±11.25	22	0	38.5±9.7	17.4±10.01 years	yes	no	not mentioned
Klauser et al., 2015 (43)	33	36	34.71±9.93	27	33	35.02±9.72	9.04±6.52 years	yes	yes	no
Kong et al., 2014 (44)	28	50	32.07±9.27	28	39	34.42±8.24	2.11±0.9 months	no	yes	no
Lai et al., 2010 (45)	15	27	34.3±9.87	16	31	37.91±8.76	17.5±8.98 weeks	no	yes	no
Lai et al., 2014 (46)	27	44	38.29±11.80	38	47	36.57±5.46	4.68±1.50 months	no	yes	no
Lai et al., 2015 (47)	54	46	40.38±10.51	53	47	40.07±8.99	5.03±1.62 months	no	yes	no
Lan et al., 2016 (48)	27	44	39.2±16.7	27	33	41.5±16.0	-	yes	yes	not mentioned
Lee et al., 2011 (49)	51	10	45.7±8.04	47	11	46.0±9.1	46.7±76 months	yes	no	not mentioned
Leung et al., 2009 (50)	17	0	45.8±9.8	17	0	45.5±8.5	84±49.1 months	yes	yes	not mentioned
Machino et al., 2014 (51)	29	55	38.66±8.36	29	55	39.57±8.29	52.55±57.81 months	yes	yes	no

Matsubara et al., 2016 (52)	27	37	48.3±13.0	34	35	48.65±12.95	49.5±65.1 months	yes	no	no
Matsuo et al., 2017 (53)	909	35	40.5±14.05	639	49	41.75±13.9	7.37±7.47 years	yes	no	PD (n=28), GAD (n=39), specific phobia (n=21), other ANX (n=35)
Modinos et al., 2014 (54)	46	69	25.3±4.3	23	13	44.6±5.5	-	-	no	not mentioned
Nakano et al., 2014 (55)	54	50	45.4±16.1	36	39	49.0±11.4	66.7±80.9 months	yes	no	no
Opel et al., 2016 (56)	20	50	36.3±12.1	20	50	37.9±10.9	139.4±100 months	yes	yes	not mentioned
Ozalay et al., 2016 (57)	24	0	47.3±5.6	24	0	46.2±3.9	13.3±8 months	yes	yes	no
Peng et al., 2011 (58)	30	37	45.9±9.0	22	36	46.7±8.9	8.6±6.5 months	yes	no	no
Peng et al., 2014 (59) without suicide history	28	54	28.61±5.45	18	33	31.06±7.39	-	yes	yes	no
with suicide history	28	54	28.61±5.45	20	35	27.75±7.21	-	yes	yes	no
Qi et al., 2014 (60) without anxiety disorder	28	54	28.61±5.45	18	39	31.06±7.39	-	no	yes	no
with anxiety disorder	28	54	28.61±5.45	20	55	28.65±8.18	-	no	yes	ANX (n=20)
Qiu et al., 2016 (61)	15	33	33.7±9.9	12	33	34.4±10.1	-	no	yes	no
Redlich et al., 2014 (62)	58	36	37.7±9.7	58	38	37.6±10.8	131.7±107.3 months	yes	yes	PD (n=12), social phobia (n=10), GAD (n=15), specific phobia (n=3), PTSD (n=8)
Ries et al., 2009 (63)	32	44	68.4±7.4	15	33	66.3±5.3	-	no	no	no
Salvadore et al., 2011 (64) chronic MDD	107	44	36.2±10.3	58	36	38.8±11.1	18.4±10.5 years	no	yes	no
currently-remitted MDD	107	44	36.2±10.3	27	22	40.2±12.2	15.1±12.2 years	no	yes	no
Scheuerecker et al., 2010 (65)	15	67	35.5±10.9	13	77	37.9±10.1	52.3±71.5 months	no	no	no
Serra-Blasco et al., 2013 (66)	32	28	46±8.3	22	18	49±8	-	yes	yes	no
Shad et al., 2012 (67)	22	55	16.0±2.1	22	50	15.0±2.1	-	no	yes	ANX (n=2)
Shah et al., 1998 (68)	20	65	49.3±11.8	20	65	48.9±9.8	263±133 weeks	yes	no	no

Shen et al., 2016 (69)	130	38	30.09±7.03	147	34	30.58±7.41	early onset 9.02±7.30 months / late onset 7.98±7.66 months	no	yes	no
Soriano-Mas et al., 2011 (70)	40	43	59.23±7.09	70	41	61.56±9.68	10.45±10.08 years	yes	yes	no
Sprengelmeyer et al., 2011 (71)	21	43	42.0±12.9	17	47	45.6±12.3	-	yes	yes	not mentioned
Stratmann et al., 2014 (72)	132	44	37.82±11.42	132	42	37.86±11.87	first episode: 14.66±15.73 months / recurrent episodes: 121.75±109.64 months	yes	yes	ANX (n=41)
Ueda et al., 2016 (73)	48	73	41.2±11.4	30	57	44.3±13.0	-	no	yes	no
van Tol et al., 2010 (74)	65	37	40.54±9.71	68	35	37.16±10.24	-	yes	yes	ANX (n=21)
Vasic et al., 2008 (75)	14	57	31.4±9.6	15	60	37.4±8.5	43.4±37.3 months	yes	yes	no
Vasic et al., 2015 (76)	29	38	34.5±10.7	43	40	37.1±10.9	7.2±7.1 years	yes	yes	no
Wagner et al., 2008 (77)	16	17	38.8±9.1	15	17	41.4±9.2	high risk pateints: 8.9±9.4 years/ no-high risk: 3±3.2 years	-	no	no
Wagner et al., 2011 (78)	30	0	37.5±11.5	30	0	35.1±11.4	7.5±7.6 years	no	no	no
Wang et al., 2012 (79)	18	50	35±12	18	50	34±13	5±4 months	no	yes	no
Wang et al., 2014 (80)	10	0	29.8±7.5	13	0	30.9±9.1	-	no	yes	no
Wang et al., 2016 (81)	35	54	33.28±8.83	25	56	32.11±11.25	0.7±0.2 years	-	yes	no
Watanabe et al., 2015 (82)	45	73	41.05±11.15	29	55	45.5±12.7	-	no	yes	no
Wehry et al., 2015 (83)	41	34	13±2	14	21	14±3	-	-	yes	no
Yang et al., 2015 (84)	51	39	31.14±9.3	51	39	30.98±9.5	9.71±12.5 months	no	yes	no
Yang et al., 2017 (85)	23	0	39.09±14.35	35	0	44.54±11.15	32.17±45.02 months	no	yes	no
Yuan et al., 2008 (86)	16	50	67.7±3.8	19	47	67.1±7.2	3.7±2.4 years	yes	yes	no
Zhang et al., 2012 (87)	32	53	21.03±1.47	33	52	20.52±1.72	-	no	yes	no
Zhao et al., 2017 (88)	41	63	27.1±7.2	37	68	26.7±7.1	2.0±0.5 years	no	no	no
Zou et al., 2010 (89)	23	43	36.6±12.9	23	43	31.1±10.4	7.6±4.4 months	no	yes	no

Table S3. ANX = anxiety disorder, GAD = generalized anxiety disorder, GMV = gray matter volume, HC = healthy controls, MDD = major depressive disorder, PD = panic disorder, PTSD = posttraumatic stress disorder, SAD = social anxiety disorder, VBM = voxel-based morphometry.

Table S4: Demographic and clinical characteristics of anxiety disorder studies included in the GMV meta-analysis.

Author, year	HC (n)	Male (%)	Mean age HC [years]	ANX (n)	Male (%)	Mean age ANX [years]	Type of ANX	Illness duration	Medication	Modulation during VBM	Comorbidity
Ahmed et al., 2012 (90)	32	47	14.49±2.23	21	52	16.17±1.68	PTSD	-	no	yes	MDD (n=8)
Asami et al., 2009 (91)	24	38	36.25±8	24	38	36.3±9.8	PD	3.9±3.4 years	yes	yes	MDD (n=3)
Bossini et al., 2017 (92)	18	79	41±6	19	53	40±9	PTSD	-	no	yes	no
Carrion et al. 2009 (93)	24	58	11±2.73	24	58	11±2.24	PTSD	-	no	yes	MDD (n=4)
Chen et al., 2006 (94)	12	33	33.25±5.27	12	33	34.56±4.91	PTSD	-	no	no	no
Chen et al., 2012 (95)	20	-	37.6±7.0	10	-	40.8±6.8	Recent onset PTSD	-	no	yes	no
Cheng et al., 2015 (96)	30	70	26.2±6.6	30	70	26.3±8.1	PTSD	12.5±2.7 months	no	no	no
Corbo et al., 2005 (97)	14	43	33.29±12.31	14	43	33.36±12.06	PTSD	-	no	no	MDD (n=7)
Frick et al., 2014 (98)	29	45	23.7±2.0	48	50	33.8±9.3	SAD	-	no	yes	MDD (n=3)
Gold et al., 2016 (99)	53	45	13,8±2,5	39	51	12,7±3,1	ANX	-	no	yes	no
Hakamata et al., 2007 (100)	70	0	46.0±6.9	14	0	45.6±6.2	PTSD	-	no	yes	MDD (n=7)
Herringa et al., 2012 (101)	15	93	30.1±6.3	13	85	28.9±4.2	PTSD	-	no	yes	no
Hilbert et al., 2015 (102)	24	29	32.25±9.33	19	16	33.47±8.90	GAD	-	no	yes	MDD (n=12)
Irle et al., 2014 (103)	64	52	32±10	67	48	31±10	SAD	15±9 years	yes	yes	MDD (n=16)
Kasai et al., 2008 (104)	23	-	51.8±2.3	18	-	52.8±3.4	PTSD	-	no	no	MDD (n=2)
Lai et al., 2012 (105)	21	48	41.14±11.81	30	37	47.03±10.63	late-onset PD	-	no	yes	no
Lai et al., 2015 (47)	54	46	40.38±10.51	53	47	43.283±10.11	PD	5.35±2.37 months	no	yes	no
Liao et al., 2013 (106)	25	52	16.71±0.22	26	50	16.83±0.22	GAD	-	no	yes	no
Makovac et al., 2015 (107)	19	16	29.2±9.8	19	16	30±6.9	GAD	16.78±8.01 years	yes	yes	no
Meng et al., 2013 (108)	19	68	21.58±3.72	20	70	21.80±3.68	SAD	50.50±45.82 months	no	no	no
Moon et al., 2014 (109)	22	59	33.4±9.7	22	59	37.0±10.7	GAD	4.5±6.6 years	no	yes	not mentioned
Moon et al., 2015 (110)	17	65	35.6±6.1	17	65	37.4±11.7	GAD	4.7±7.3 years	yes	yes	MDD (n=17)
Moon et al., 2017 (111)	20	65	35.0±9.3	20	65	36.9±11.3	GAD	4.3±6.8 years	yes	yes	not mentioned
Na et al., 2013 (112)<br>with agoraphobia	22	50	40.18±12.38	12	42	43.08±9.63	PD with agoraphobia	-	-	yes	no
without agoraphobia	22	50	40.18±12.38	10	80	36.70±11.63	PD	-	-	yes	no
Nardo et al., 2010 (113)	22	73	40.8±8.9	21	71	41.7±9.4	PTSD	2.5±1.6 years	-	no	no
Nardo et al., 2013 (114)	17	65	41.59±9.04	15	80	43.33±8.35	PTSD	-	no	yes	no

O'Doherty et al., 2017 (115)	25	48	31.7±6.0	25	48	34.0±8.4	PTSD	4.61±2.57 years	yes	yes	no
Rocha-Rego et al., 2012 (116)	16	44	44.9±6.60	16	44	43.3±5.78	PTSD	3.0±4.8 years	-	yes	no
Schienle et al., 2011 (117)	15	0	23.7±3.7	16	0	22.9±4.1	GAD	3.1±4.7 years	no	yes	not mentioned
Strawn et al., 2013 (118)	28	39	13±2	15	47	13±2	GAD	-	no	yes	no
Strawn et al., 2015 (119)	27	44	14.8±3.9	38	26	14.4±3.0	GAD	-	no	yes	no
Sui et al., 2010 (120) healthy comparison	12	0	26.42±3.45	11	0	25.55±4.01	PTSD	-	no	no	no
victims of rape – non PTSD	8	0	27.50±4.00	11	0	25.55±4.01	PTSD	-	no	-	no
Talati et al., 2013 (121) SAD	37	55	31.4±9.1	33	19	31.5	SAD	-	yes	yes	MDD (n=11)
PD	20	55	31.4±7.8	16	19	34.1	PD	-	yes	-	MDD (n=3)
Tavanti et al., 2012 (122)	25	32	38.08±11.01	25	32	38.16±10.90	PTSD	7.36±8.62 years	no	yes	no
Thomaes et al., 2010 (123)	30	0	35.2±12.3	33	0	35.3±9.8	PTSD	-	yes	yes	MDD (n=21)
Tükel et al., 2015 (124)	27	44	27.70±5.83	27	44	27.70±6.67	SAD	13.76±6.99 years	no	yes	no
van Tol et al., 2010 (74)	65	37	40.54±9.71	68	26	35.96±9.45	ANX	-	yes	yes	MDD (n=37)
Yamasue et al., 2003 (125)	16	63	44.4±14	9	56	44.6±16	PTSD	-	yes	no	MDD (n=1)
Yoo et al., 2005 (126)	18	61	32.0±5.8	18	50	33.3±7.1	PD	3.6±2.2 years	no	yes	no
Zhang et al., 2011 (127)	10	100	34.40±5.37	10	100	40.8±6.83	PTSD	-	no	yes	no
Zhao et al., 2017 (88)	41	63	27.1±7.2	24	63	24.5±4.0	SAD	7.6±3.8 years	no	yes	no

Table S4. ANX = anxiety disorder, GAD = generalized anxiety disorder, GMV = gray matter volume, HC = healthy controls, MDD = major depressive disorder, PD = panic disorder, PTSD = posttraumatic stress disorder, SAD = social anxiety disorder, SP = specific phobia, VBM = voxel-based morphometry.

Table S5: Demographic and clinical characteristics of chronic pain studies included in the GMV meta-analysis.

Author, year	HC (n)	Male (%)	Mean age HC [years]	CP (n)	Male (%)	Mean age CP [years]	Type of chronic pain	Illness duration	Medi-cation	Modulation during VBM	Comorbidity
Absinta et al., 2012 (128)	19	63	42	15	87	44	Cluster headache	10 years	no	yes	no
Apkarian et al., 2004 (129)	26	-	43.6	26	-	43.7	Chronic back pain	8.6 years	yes	no	no
Arkink et al., 2016 (130) Chronic cluster headache	48	37	47±12	23	83	48±9	Cluster headache	14±9 years	yes	yes	not mentioned
Probable cluster headache	48	37	47±12	14	43	50±10	Cluster headache	19±10 years	yes	yes	not mentioned
Chronic paroxysmal hemicrania	48	37	47±12	9	33	48±9	Chronic paroxysmal hemicrania	15±7 years	yes	yes	not mentioned
Migraine with aura	48	37	47±12	14	7	47±9	Migraine with aura	28±10 years	yes	yes	not mentioned
Episodic cluster headache	48	37	47±12	24	83	45±9	Cluster headache	16±12 years	yes	yes	not mentioned
Migraine without aura	48	37	47±12	19	5	47±8	Migraine without aura	29±15 years	yes	yes	not mentioned
As-Sanie et al., 2012 (131) endometriosis-associated	26	0	25.9±1.6	17	0	26.1±1.5	Chronic pelvic pain	5.5 years	-	yes	no
without endometriosis	26	0	24.8±1.2	6	0	24.2±1.9	Chronic pelvic pain	3.75 years	-	yes	no
Baliki et al., 2011 (132) CBP	46	43	38.77±12.5	36	64	48.20±11.38	Chronic back pain	12.3 years	yes	no	no
CRPS	46	43	38.77±12.5	28	14	40.57±7.4	CRPS	3.16 years	yes	no	no
OA	46	43	38.77±12.5	20	80	53.50±7.4	Knee osteoarthritis	11.6 years	yes	no	no
Barad et al., 2014 (133)	15	0	44.1	15	0	44.0	CRPS	2-206 months	yes	no	no
Bishop et al., 2017 (134)	31	42	44.12±13.15	74	19	45.7±13.03	Musculoskeletal pain	11±9.15 years	no	yes	no

Burgmer et al., 2009 (135)	14	0	46.9±6.8	14	0	51.0±7.3	Fibromyalgia	10±6 years	yes	yes	no
Buckalew et al., 2008 (136)	8	63	69.9±3.9	8	50	74.5±4.2	Chronic low back pain	-	no	no	no
Ceko et al., 2013 (137) older subjects	13	0	55.4±3.7	14	0	55.0±2.9	Fibromyalgia	12.1±9.0 years	yes	yes	no
younger subjects	15	0	43.1±5.3	14	0	42.4±5.9	Fibromyalgia	8.8±7.1 years	yes	yes	no
Davis et al., 2008 (138)	11	36	24-50	9	33	30-58	Irritable bowel syndrome	-	no	yes	no
Diaz-Piedra et al., 2016(139)	23	0	39.7±5.4	23	0	41.6±4.4	Fibromyalgia	102.6±75.9 months	yes	no	no
Fallon et al., 2013 (140)	15	0	39.4±8.7	16	0	38.5±8.45	Fibromyalgia	9.1±6.8 years	yes	yes	no
Fritz et al., 2016 (141)	432	57	48.92±13.96	111	30	53.12±11.77	Chronic back pain	-	yes	yes	no
Geha et al., 2008 (142)	22	-	40.5±2.3	22	14	40.7±2.3	CRPS	3.57 years	yes	yes	MDD (n=1)
Gerstner et al., 2011 (143)	9	0	24.8±1.4	9	0	25.4±2.5	Myofascial-type TMD	2.5±2.1 years	yes	yes	no
Gustin et al., 2011 (144)	30	20	53.6±3.2	21	19	54.7±2.1	Trigeminal neuropathic pain	8.5±2.1 years	yes	yes	not mentioned
Gwilym et al., 2010 (145)	16	50	68	16	50	68	Primary hip osteoarthritis	-	no	yes	no
Holle et al., 2011 (146)	14	36	65.7	14	36	65.9	Hypnic headache	-	no	no	no
Ivo et al., 2013 (147)	14	43	54	14	43	54	Chronic low back pain	10 years	-	yes	MDD (n=7)
Keltner et al., 2017 (148)	169	81	43±8	64	78	46±8	Distal neuropathic pain	-	-	yes	MDD (n=21)
Kim et al., 2008 (149)	33	12	33.8±10.5	20	15	33.7±11.3	Episodic migraine	9.8±6.0 years	yes	yes	no
Kuchinad et al., 2007 (150)	10	0	45	10	0	52	Fibromyalgia	62.6±31.9 months (homozygotes), 72.9±37.6 months (met carriers)	no	no	no
Lewis et al., 2018 (151)	18	39	71±8	29	52	68±10	Knee osteoarthritis	-	no	yes	no

Li et al., 2018 (152)	16	100	31.3±11	16	75	41.6±13.6	Chronic low back pain	10.2±9.8 years	no	yes	no
Liao et al., 2018 (153)	30	13	55.2±5.7	30	13	56.5±6.8	Knee osteoarthritis	7.3±5.1 years	yes	yes	no
Liu et al., 2015 (154)	111	0	21.3±0.9	135	0	21.7±2.1	Migraine without aura	62.6±31.9 months (homozygotes), 72.9±37.6 months (met carriers)	no	yes	no
Liu et al., 2017 (155)	50	-	22.6±0.2	80	-	22.8±0.3	Migraine without aura	59.8±4.7 months	no	yes	no
Luchtmann et al., 2014 (156)	12	-	43.9±12.9	12	-	43.9±12.9	Chronic low back pain	-	-	yes	not mentioned
Marciszewski et al., 2017 (157)	57	25	28.3±1.3	25	16	30.2±2.0	Migraine	14.6±2.2 years	yes	yes	no
Martikainen et al., 2013 (158)	16	50	36±11	16	50	38±11	Chronic nonspecific back pain	5.0±3.8 years	no	yes	no
Mehnert et al., 2017 (159)	54	17	32.6±11.5	54	17	34.3±11.9	Migraine	18.1±12.3 years	-	yes	not mentioned
Mole et al., 2014 (160)	18	-	-	30	-	52.775	Spinal cord injury with neuropathic pain	11.1 years	-	no	no
Naegel et al., 2014 (161)	78	72	42.78±11.44	91	78	45.52±10.61	Cluster headache	14.42±9.39 years	yes	yes	no
Neeb et al., 2017 (162) episodic migraine	21	29	49.40±7.79	21	29	49.36±7.62	Chronic migraine	24.43±8.3 years	yes	yes	no
chronic migraine	21	29	49.40±7.79	21	29	49.04±7.46	Episodic migraine	26.71±14.42 years	yes	-	no
Obermann et al., 2013 (163)	49	43	61.8±9	60	40	62±13.2	Trigeminal neuralgia	8.3±6.7 years	yes	yes	no
Obermann et al., 2014 (164)	17	18	42.17±9.26	17	18	42.71±10.05	Vestibular migraine	6.17±4.51 years	yes	yes	no
Pleger et al., 2014 (165)	20	45	41.6±9.6	20	45	41.8±9.8	CRPS	-	-	yes	not mentioned
Pomares et al., 2017 (166)	25	0	61±7.6	26	0	61±5.4	Fibromyalgia	16±9 years	no	yes	no

Riederer et al., 2012 (167)	29	24	41.7±12.7	29	24	41.4±12.7	Medication overuse headache	15.14±11.0 years	yes	yes	no
Rocca et al., 2006 (168)	15	13	38.6	16	6	42.7	Migraine	24.8 years	yes	yes	no
Rocca et al., 2014 (169)	15	47	13.3	12	50	14.2	Episodic migraine	3.7 years	yes	yes	no
Rodriguez-Raecke et al., 2009 (170)	32	41	63.9±8.8	32	41	66.8±9.0	Unilateral primary hip osteoarthritis	7.35 years	yes	yes	MDD (n=3)
Ruscheweyh et al., 2011 (171)	31	61	63±9	45	36	66±7	Ongoing chronic pain	264±193 months	yes	no	no
Russell et al., 2018 (172)	11	18	59±7.4	28	14	62±7.7	Hand osteoarthritis	-	yes	no	no
Schmidt-Wilcke et al., 2006 (173)	18	50	49.9±8.7	18	50	50.4±6.8	Chronic back pain	176±87.6 months	yes	no	no
Schmidt-Wilcke et al., 2007 (174)	22	9	50.7±7.3	20	5	53.6±7.7	Fibromyalgia	173±86.5 months	yes	yes	not mentioned
Schmidt-Wilcke et al., 2008 (175)	31	0	32.3±12.6	35	9	32.4±9.2	Migraine	-	yes	no	no
Schmidt-Wilcke et al., 2010 (176)	11	18	51.3±8.6	11	18	52.2±8.9	Persistent idiopathic facial pain	58.3 months	-	yes	not mentioned
Schmitz et al., 2008 (177)	28	0	42.50±9.31	28	0	43.5±8.21	Migraine	30.5±11.43 years	no	no	no
Seminowicz et al., 2010 (178)	48	0	31.1±12.3	55	0	32.2±12.3	Irritable bowel syndrome	11.1±7.7 years	no	no	MDD (n=3), ANX (n=6)
Shokouhi et al., 2017 (179)	16	31	44.4±11.6	12	17	51.1±12.7	CRPS	5.9±2.9 months	no	no	no
Tu et al., 2010 (180)	32	0	23.81±2.80	32	0	23.84±2.99	Primary dysmenorrhea	10.19±3.25 years	no	yes	no
Valet et al., 2009 (181)	25	0	51.7±7.2	14	0	51.1±11.1	Chronic pain	9.8±7.2 years	no	no	MDD (n=7)
Valfrè et al., 2008 (182)	27	26	34.9±8.6	27	22	34.9±8.4	Migraine	20.6±8.9 years	-	yes	not mentioned
Vartiainen et al., 2009 (183)	28	36	32	8	13	47	Chronic widespread unilateral pain	-	-	no	not mentioned
Wang et al., 2017 (184)	40	42	55.89±8.06	41	42	55.87±8.38	Trigeminal neuralgia	7.05±5.32 years	yes	yes	no

Wilcox et al., 2015 a (185)	40	18	48.3±2.1	22	18	46.5±2.6	TMD	9.7 years	yes	yes	no
Wilcox et al., 2015 b (186)	42	19	48.6±2.0	21	19	48.7±1.7	Trigeminal neuropathy	5.5 years	yes	yes	not mentioned
Wood et al., 2009 (187)	20	0	40.05±10.01	30	0	42.03±8.43	Fibromyalgia	-	no	no	no
Yang et al., 2013 (188)	49	78	35.2±9.7	49	78	35.7±9.2	Cluster headache	8.6±6.9 years	yes	yes	no
Yoon et al., 2013 (189)	10	60	39.5±8.6	10	70	39.5±8.6	Chronic neuropathic pain	1.5 years	-	yes	no
Younger et al., 2010 (190)	15	0	38±13.7	15	0	38±13.7	Myofascial-type TMD	4.4±2.9 years	yes	no	no
Zhang et al., 2017 (191)	32	25	38.8±10.02	32	25	38.3±10.16	Migraine without aura	9.5±6.23 years	no	yes	no
Zhang et al., 2018 (192)	34	38	43.32±10.07	29	34	48.14±11.89	Trigeminal neuralgia	6.02±4.35 years	yes	yes	no

Table S5. CP = chronic pain, CRPS = complex regional pain syndrome, GMV = gray matter volume, HC = healthy controls, MDD = major depressive disorder, OA = osteoarthritis, TMD = temporomandibular disorders, VBM = voxel-based morphometry.

Table S6: Demographic and clinical characteristics of major depressive disorder studies included in the iFC meta-analysis.

Author, year	HC (n)	Male (%)	Mean age HC [years]	MDD (n)	Male (%)	Mean age MDD [years]	Illness duration	Symptom severity	Medicated	Global signal regression	Comorbidity
Alalade et al., 2011 (193)	18	39	71.2	11	39	64.9	31,3 years	MADRS: 17.5	yes	yes	no
Alexopoulos et al., 2012 (194)	10	-	68.8	16	-	69	-	MADRS: 23.5	no	no	no
Andreescu et al., 2013 (195)	46	28	72.89	47	28	68.72	20,09 years	HAM-D: 19.06	no	no	no
Avery et al., 2014 (196)	20	40	33	20	35	36	61 months	HAM-D: 23.1	no	no	PTSD (n=3), social phobia (n=4), PD (n=1), simple phobia (n=1)
Bai et al., 2018 (197)	57	39	36.68	50	34	38.68		HAM-D: 22.78	yes	yes	no
Berman et al., 2011 (198)	15	33	23	15	33	25.7	-	BDI: 30	yes	no	not mentioned
Bessette et al., 2018 (199)	35	40	21.45	47	28	22.16	2,72 years	HAM-D: 1.64	no	no	Comorbid anxiety disorder
Cao et al., 2012 (200)	32	53	26.09	42	43	29.15	13,11 years	HAM-D: 23.60	no	no	no
Chen et al., 2018 (201)	47	21	29.70	36	56	30.70	32,1 months	HAM-D: 28.0	no	no	no
Connolly et al., 2013 (202)	45	38	16.1	30	37	16	-	BDI: 27.3	no	no	GAD (n=12), specific phobia (n=2), other anxiety disorder (n=1), PTSD (n=2)
Connolly et al., 2017 (203)	53	38	16.1	48	40	16.1	-	RADS: 64.7	no	no	GAD (n=12), specific phobia (n=4), other anxiety disorder (n=1), PTSD (n=5)
Crowther et al.,2015 (204)	20	30	31.1	23	22	33.09	32,96 months	BDI: 26.04	no	no	no
Cullen et al., 2014 (205)	29	24	16.0	41	22	15.7	10 months	BDI: 29	no	no	GAD (n=16), PTSD (n=2), SAD (n=3), PD (n=2), specific phobia (n=3), social phobia (n=4)
Davey et al., 2012 (206)	20	40	19.9	18	33	18.9	10,5 months	BDI: 35.4	yes	yes	ANX (n=6)
DelDonno et al., 2017 (207)	28	50	21	42	24	21	-	HAM-D: 4.17	no	yes	Comorbid anxiety disorder
Deng et al., 2016 (208)	29	52	26.76	29	31	28.69	-	SDS: 62.72	no	no	no

de Kwaasteniet et al., 2015 (209)	18	44	51.5	17	47	52.5	20,3 years	HAM-D: 21.8	yes	no	no
Ellard et al., 2018 (210)	39	51	25.9	35	63	42.77	-	HAM-D: 16.82	yes	yes	ANX (n=29)
Evans et al., 2018 (211)	25	40	33	33	39	36	20 years	-	no	no	not mentioned
Furman et al., 2011 (212)	19	0	33.2	21	0	39.2	-	BDI: 31.3	yes	yes	PD (n=2), PTSD (n=2), SAD (n=5)
Gabbay et al., 2013 (213)	21	43	16.3	21	43	17.1	13,6 months	BDI: 25.3	no	yes	Anxiety disorder (n=10), GAD (n=8)
Gandelman et al., 2018 (214)	21	57	68.3	79	66	66.3	-	MADRS: 27.3	yes	no	no
Gong et al., 2017 (215)	42	55	41.31	75	44	40.41	-	HAM-D: 1.1	no	yes	no
Guo et al., 2013 a, b (216) treatment-resistant depression	19	53	24.37	23	48	27.35	27,43 months	HAM-D: 24.52	no	yes	no
treatment-sensitive depression	19	53	24.37	22	55	28.09	2,95 months	HAM-D: 25.89	no	yes	no
Guo et al., 2013 c, d (217)	24	58	24.04	24	54	25.58	4,96 months	HAM-D: 25.75	no	no	no
Guo et al., 2015 a, b (218)	44	45	29.39	44	50	27.52	19,61 months	HAM-D: 25.18	no	no	no
Jacobs et al., 2016 (219)	26	46	21.15	51	29	21.49	5,23 years	HAM-D: 7.78	no	no	ANX (n=27)
Kenny et al., 2010 (220)	17	65	75.7	16	50	76.4	29 years	MADRS: 7.1	yes	no	not mentioned
Kerestes et al., 2015 (221)	21	48	19.2	21	48	19.3	-	MADRS: 33.7	no	no	no
Kim et al., 2016 (222)	20	70	14.5	22	64	13.9	-	CDI: 40.0	no	no	no
Li et al., 2015 (223)	26	42	71.19	22	18	68.14	35,19 years	GDS: 16.36	yes	no	no
Liu et al., 2018 (224)	40	45	64.68	27	37	67.33	-	HAM-D: 31.78	-	no	no
Lois et al., 2016 (225)	25	32	42.52	25	28	42.82	12,4 years	HAM-D: 0.88	yes	no	no
Lui et al., 2011 (226) nonrefractory depression	48	65	35	32	66	32	22 months	HAM-D: 23.0	no	no	no
refractory depression	48	65	35	28	64	33	193 months	HAM-D: 23.2	no	no	no
Ma et al., 2012 (227) treatment-resistant depression	17	59	24.24	18	61	27.39	35,5 months	HAM-D: 23.89	yes	yes	no
treatment-sensitive depression	17	59	24.24	17	59	26.71	2,59 months	HAM-D: 25.58	no	yes	no
Pannekoek et al., 2014 (228)	26	12	14.7	26	12	15.4	-	CDI: 18.6	no	yes	ANX (n=18)
Peng et al., 2015 (229)	16	44	33.75	16	44	34.43	8,01 weeks	HAM-D: 30.88	no	yes	no

Peng et al., 2018 (230)	19	47	33.89	19	47	33.58	-	HAM-D: 24.89	no	yes	no
Penner et al., 2016 (231)	24	50	23.8	24	33	21.2	13,5 months	MADRS: 22.7	yes	no	no
Peters et al., 2016 (232)	10	30	15.8	23	43	15.61	-	RADS: 60.48	yes	no	ANX (n=8)
Philippi et al., 2018 (233)	30	0	27.1	34	0	27.9	24,2 months	BDI: 19.5	no	no	ANX (n=24)
Poeppl et al. 2016 (234)	76	47	37.34	72	47	38.43	12,31 years	BDI: 22.75	yes	no	Anxiety disorder (n=10), PTSD (n=1)
Rao et al., 2016 (235)	23	48	21.1	34	26	21.1	5,3 years	HAM-D: 2.4	no	no	not mentioned
Renner et al., 2017 (236)	18	28	42.67	18	28	41.17	-	BDI: 30.5	yes	yes	no
Sawaya et al., 2015 (237)	21	19	38.33	21	19	37.29	<24 months	HAM-D: 23.29	no	yes	no
Scheinost et al., 2018 (238)	20	40	32.6	17	29	34.3	10,2 years	MADRS: 24.1	no	no	Comorbid anxiety disorder
Schilbach et al., 2016 (239)	106	48	36.99	102	48	37.75	10,63 years	-	yes	no	not mentioned
Sheline et al., 2010 (240)	17	29	30.9	18	61	35.9	-	HAM-D: 20.2	no	no	no
Shu et al., 2014 (241)	29	45	71.41	31	35	68.13	<5 years	-	no	yes	no
Späti et al., 2015 (242)	35	43	32.7	21	52	36.6	-	BDI: 26.4	no	no	ANX (n=8)
Straub et al., 2015 (243)	19	21	16.35	19	21	16.76	-	CDRS-R: >36	-	yes	Social phobia (n=4), specific phobia (n=1)
Tadayonnejad et al., 2016 (244)	20	35	31.8	19	37	27.4	-	HAM-D: 19.1	no	no	not mentioned
Tahmasian et al., 2013 (245)	20	45	49.6	21	48	51.0	14,7 years	HAM-D: 23.8	yes	yes	GAD (n=6)
van Tol et al., 2014 (246)	20	37	33.75	20	35	38.25	57,2 months	HAM-D: 15.5	yes	yes	no
Wang et al., 2018 (247)	139	47	30.32	93	43	29.74	33.4 months	HAM-D24: 25.13	no	yes	ANX (n=35)
Workman et al., 2016 (248)	38	34	36.2	63	35	36.4	13,9 years	BDI: 3.6	no	no	PD with agoraphobia (n=1), PTSD (n=1)
Workman et al., 2017 (249) recurring episode	38	34	36.2	17	35	35.9	16,4 years	BDI: 5.2	no	no	PD with agoraphobia (n=1)
Resilient	38	34	36.2	30	40	37.6	17,4 years	BDI: 2.6	no	no	no
Wu H et al., 2016 (250)	34	44	29.71	34	40	29.88	53,41 months	HAM-D: 32.91	yes	no	no
Wu X et al., 2016 (251)	19	47	33.47	19	41	33.47	-	HAM-D: 24.89	no	yes	no
Yang et al., 2016 a (85)	23	0	39.09	35	0	44.54	32.17 months	HAM-D: 28.29	no	no	no
Yang et al., 2016 b (252)	19	42	33.8	19	42	33.3	-	HAM-D: 15.8	-	yes	no
Yang et al., 2017 (253)	36	47	29.08	40	53	28.75	6.16 months	BDI: 32.1	no	yes	no
Ye et al., 2012 (254)	30	36	45.9	22	36	46.7	8,6 months	HAM-D: 18.5	yes	no	no

Ye et al., 2017 (255) young-adult	35	49	24.8	34	50	24.15	-	HAM-D: 23.5	no	no	no
old-adult	46	22	37.22	35	26	37.14	-	HAM-D: 23.69	no	no	no
Yin et al., 2015 a (256)	39	49	65.40	32	34	67.17	-	HAM-D: 30.83	no	yes	no
Yin et al., 2015 b (257)	33	42	65.21	26	31	67.39	-	HAM-D: 30.18	no	yes	no
Yuan et al., 2014 (258)	27	19	36	27	19	36.56	-	HAM-D: 22.11	no	no	no
Zhang et al., 2015 (259) deficiency syndrome	20	15	47.75	24	4	48.42	-	HAM-D: 26.71	no	no	no
excess syndrome	20	15	47.75	21	29	41.9	-	HAM-D: 27.86	no	no	no

Table S6. ANX = anxiety disorder, BDI = Beck Depression Inventory, CDI = Children's Depression Inventory, CRDS-R = Children's Depression Rating Scale Revised, GAD = generalized anxiety disorder, GDS = Geriatric Depression Scale, HAM-D = Hamilton Depression Rating Scale, HC = healthy controls, iFC = intrinsic functional connectivity, MADRS = Montgomery-Asberg Depression Rating Scale, MDD = Major depressive disorder, PD = panic disorder, PTSD = posttraumatic stress disorder, RADS = Reynolds Adolescent Depression Scale, SDS = Self-rating depression scale.

Table S7: Demographic and clinical characteristics of anxiety disorder studies included in the iFC meta-analysis.

Reference	HC (n)	Male (%)	Mean age HC [years]	ANX (n)	Male (%)	Mean age ANX [years]	Type of ANX	Illness duration	Symptom severity	Medicated	Global signal regression	Comorbidity
Aghajani et al., 2016 (260)	23	9	15.52	19	11	16.16	PTSD	5.23 years	TSCC: 50.93	yes	no	MDD (n=7)
Andreescu et al., 2014 (261)	31	42	52.18	24	21	49.54	GAD	9.5 years	HARS: 20.9	no	no	MDD (n=5)
Arnold Anteraper et al., 2014 (262)	17	47	25	17	47	24.7	SAD	-	LSAS: 77.9	no	no	MDD (n=4)
Birn et al., 2014 (263)	14	29	10.2	14	29	9.9	ANX	-	-	yes	no	MDD (n=3)
Bluhm et al., 2009 (264)	15	0	38	17	0	39	PTSD	-	CAPS: 76.9	yes	no	MDD (n=6), dysthymic disorder (n=1), depression disorder not otherwise specified (n=1)
Brown et al., 2014 (265)	22	73	44.0	20	80	44.1	PTSD	-	CAPS: 66.4	yes	no	MDD (n=9)
Chen et al., 2018 (266) trauma-exposed control	33	21	48.5	27	26	48.4	PTSD	-	CAPS: 78.2	no	no	no
healthy control	30	23	49.9	27	26	48.4	PTSD	-	CAPS: 78.2	no	no	no
Cui et al., 2017 (267)	20	70	21.65	21	74	22.05	SAD	43.81 months	LSAS: 53.9	no	no	no
Dodhia et al., 2014 (268)	18	100	29.89	18	100	29.39	GSAD	-	LSAS: 81.67	no	no	no
Dorfman et al., 2016 (269)	36	42	13	35	40	13.2	ANX	-	-	no	no	no
Geiger et al., 2016 (270)	15	47	28.47	18	39	29.56	SAD	20.5 years	LSAS: 88.6	no	no	no
Hahn et al., 2011 (271)	27	41	27.7	10	90	28.6	SAD	-	STAI: 42.1	no	no	no
Hamm et al., 2014 (272)	23	43	14.6	33	33	13.9	GAD + SP	-	PARS: 22	no	yes	no
Harricharan et al., 2016 (273) non-dissociative subtype	40	35	35.0	60	42	37.8	PTSD	-	CAPS: 67.9	no	no	MDD (n=11)
dissociative subtype	40	35	35.0	37	22	40.4	PTSD	-	CAPS: 81.6	no	no	MDD (n=23)
Harricharan et al., 2017 (274) non-dissociative subtype	40	35	35.0	60	42	37.8	PTSD	-	CAPS: 67.9	yes	no	MDD (n=11)
dissociative subtype	40	35	35.0	41	20	41.1	PTSD	-	CAPS: 81.6	yes	no	MDD (n=23)
Holmes et al., 2018 (275)	18	67	35.2	21	43	35.8	PTSD	17.8 years	MADRS: 19.0	no	yes	MDD (n=16)

Jung et al., 2018 (276)	42	45	24.7	36	47	25.4	SAD	9.3 years	LSAS: 78.3	yes	no	MDD (n=4)
Li et al., 2016 (277)	21	64	38.05	22	62	39.9	GAD	-	HAMA: 18.6	no	no	no
Liao et al., 2011 (278)	18	72	21.89	18	67	22.67	SAD	49.22 months	LSAS: 54.39	no	no	no
Liu et al., 2015 (279)	20	45	15.55	26	38	15.54	GAD	11.69 months	SCARED: 37.12	no	no	no
Makovac et al., 2015 (280)	21	14	28.67	19	11	29.58	GAD	16.78 years	SRRS: 1378.95	yes	yes	no
Manning et al., 2015 (281)	33	58	29.4	53	68	29.9	SAD	16.8 years	LSAS: 81.8	no	yes	MDD (n=18)
Misaki et al., 2017 (282)	28	100	29.0	35	100	31.9	PTSD	-	CAPS: 55.2	no	no	MDD (n=1)
Nicholson et al., 2015 (283) non-dissociative subtype	40	28	32.3	36	28	37	PTSD	-	CAPS: 69.0	no	no	MDD (n=1)
dissociative subtype	40	28	32.3	13	15	37	PTSD	-	CAPS: 79.5	no	no	MDD (n=1)
Pace-Schott et al., 2017 (284)	13	15	35.0	12	17	30.2	GAD	-	STAI: 41.7	no	yes	no
Pannekoek et al., 2013 a (285)	12	42	34.0	12	42	43.8	SAD	-	FQ: 20.8	no	no	not mentioned
Pannekoek et al., 2013 b (286)	11	9	35.0	11	9	34.5	PD	-	BAI: 14.5	yes	no	not mentioned
Patriat et al., 2016 (287)	30	40	14.0	29	38	14.6	PTSD	46 months	CAPS: 70	no	no	MDD (n=17), depressive disorder NOS (n=3)
Prater et al., 2013 (288)	17	41	25.71	20	45	25.95	GSAD	-	LSAS: 79.35	yes	yes	no
Rabellino et al., 2018 (289) non-dissociative subtype	47	32	33.81	65	38	37.58	PTSD	-	CAPS-IV: 67.39	yes	no	MDD (n=12)
dissociative subtype	47	32	33.81	37	22	40.38	PTSD	-	CAPS-IV: 81.6	yes	no	MDD (n=23)
Rabinak et al., 2011 (290)	17	100	33.71	15	100	30.12	PTSD	-	CAPS: 67.35	no	no	MDD (n=2)
Roy et al., 2013 (291)	20	35	14.8	15	33	14.9	GAD	-	SCARED: 30.3	no	no	MDD (n=3)
Sripada et al., 2012 a, b (292,293)	15	100	26.6	15	100	27.3	PTSD	-	CAPS: 75.9	yes	no	MDD (n=7)
Thome et al., 2017 (294)	41	37	37.11	57	68	33.98	PTSD	-	CAPS: 69.16	no	no	MDD (n=18)
Toazza et al., 2016 (295)	19	53	16.7	18	56	17.9	ANX	-	-	no	no	MDD (n=5)
Wang et al., 2016 (296)	28	50	33.21	28	50	32.93	GAD	29.75 weeks	SAS: 64.87	no	no	no
Yuan et al., 2016 (297)	19	68	26.26	15	67	27.07	GSAD	-	LSAS: 78.87	no	no	not mentioned
Yuan et al., 2017 a (298)	43	60	30.14	43	63	29.00	SAD	-	LSAS: 69.23	yes	no	MDD (n=10)

Yuan et al., 2017 b (299)	64	55	23.78	46	61	24.8	SAD	7.63 years	LSAS: 64.79	yes	no	MDD (n=2)
Zhang et al., 2016 (300)	33	48	48.85	33	36	52.06	PTSD	-	PCL: 43.25	-	no	not mentioned
Zhu et al., 2017 (301)	34	32	35.1	27	44	36.0	PTSD	-	CAPS: 75.7	no	no	no

Table S7. ANX = anxiety disorder, BAI = Beck Anxiety Inventory, CAPS = Clinician-Administered PTSD Scale, FQ = Fear Questionnaire , GAD = Generalized anxiety disorder, LSAS = Liebowitz Social Anxiety Scale, GSAD = Generalized social anxiety disorder, HAMA = Hamilton Anxiety Rating Scale, HARS = Hamilton Anxiety Rating Scale, HC = healthy controls, iFC = intrinsic functional connectivity, MADRS = Montgomery-Asberg Depression Scale, MDD = major depressive disorder, PARS = Pediatric Anxiety Rating Scale, PCL = PTSD Checklist for DSM-5, PD = Panic disorder, PTSD = Posttraumatic stress disorder, SAD = Social anxiety disorder, SAS = Social anxiety scale, SCARED = Screen for Child Anxiety Related Disorders, SP = Social phobia, SRRS = Stress-Reactive Rumination Scale, STAI = Spielberger State and Trait Anxiety Inventory, TSCC = Trauma Symptom Checklist for Children.

Table S8: Demographic and clinical characteristics of chronic pain studies included in the iFC meta-analysis.

Reference	HC (n)	Male (%)	Mean age HC [years]	CP (n)	Male (%)	Mean age CP [years]	Type of CP	Illness duration	Symptom severity	Medicated	Global signal regression	Comorbidity
Ayoub et al., 2019 (302)	79	56	45.4	77	43	46.2	CBP	11.5 years	VAS: 4.8	-	no	not mentioned
Baliki et al., 2011 (303)	15	67	51.67	15	67	51.87	CBP	6,73 years	VAS: 69.6	yes	no	no
Bhatt et al., 2019 (304)	26	0	10.72	32	0	11.4	IBS	-		no	no	not mentioned
Bolwerk et al., 2013 (305)	12	42	60.92	12	42	61.08	CRPS	15,5 weeks	MPQ (PRI): 11.67	yes	no	no
Case et al., 2017 (306)	15	47	28.8	15	53	24.5	SCD	-	Subjective (0-10): 0.73	yes	no	not mentioned
Cauda et al., 2009 (307)	8	50	49	8	50	61	DNP	>2 years	VAS: 5.625	no	no	not mentioned
Ceko et al., 2013 (137) older subjects	13	0	55.4	14	0	55	FM	12.1 years	VAS: 2.41	yes	no	no
younger subjects	15	0	43.1	14	0	42.4	FM	8.8 years	VAS: 2.8	yes		no
Ceko et al., 2015 (308)	16	-	-	14	-	-	CLBP	4.8 years	SF-MPQ: 17.66	yes	no	not mentioned
Chen et al., 2016 (309) episodic mirgaine	32	25	41.3	18	22	33.4	Migraine	12.4 years	VAS: 8.3	yes	no	no
chronic migraine		25		16	25	42.4	Migraine	11.3 years	VAS: 7.9	yes		no
Cottam et al., 2018 (310)	19	32	65	25	48	65	Knee OA	1-38 years	VAS: 27.8	yes	no	no
Fallon et al., 2016 (311)	15	0	39.4	16	0	38.45	FM	9.13 years	FIQ: 62.37	yes	no	not mentioned
Flodin et al., 2014 (312)	24	0	45.7	16	0	48.3	FM	7.6 years	FIQ: 61.2	yes	no	no
Flodin et al., 2016 (313)	19	16	50.4	24	17	53.8	RA	-	VAS Global: 33.7	yes	no	no
Han et al., 2013 (314)	64	17	81.6	64	27	81.3	CMP	-	-	yes	no	not mentioned
He et al., 2018 (315)	20	42	23.1	30	37	22.1	TMD	17.3 months	Pain intensity score: 41.4	no	no	no
Hong et al., 2014 (316) Male	24	100	34.33	24	100	34.71	IBS	13 years	Gracely pain scale: 5.67	no	no	no
Female	24	0	30.67	24	0	33.58	IBS	13.14 years	Gracely pain scale: 4.7	no		no
Ichesco et al., 2012 (317)	8	0	24.8	8	0	25.4	TMD	2.5 years	MPQ: 6.1	no	no	no
Ichesco et al., 2014 (318)	18	0	30.67	18	0	35.8	FM	3.9 years	SF-MPQ: 9.7	no	no	no
Jiang et al., 2016 (319)	18	61	59.2	18	61	59.67	PHN	116.78 days	VAS: 6.611	no	yes	not mentioned
Khan et al., 2014 (320)	9	0	56	9	0	54	BMS	4 years	Rating scale: 2.8	no	no	not mentioned

Kilpatrik et al., 2014 (321)	85	0	35.3	82	0	38.8	IC/PBS	10.4 years	GUPI (total): 26.2	no	no	not mentioned
Kim et al., 2017 (322)	25	56	31.7	25	48	36.1	CRPS	2,8 years	VAS: 5.0	yes	no	MDD (n=11), other mood disorders (n=7), ANX (n=1)
Kong et al., 2018 (323)	20	5	52.9	21	5	53.1	FM	-	FIQ: 45.1	yes	yes	no
Kucyi et al., 2014 (324)	17	0	32.2	17	0	33.1	TMD	>3 months	Rating scale: 4.3	no	no	no
Kutch et al., 2015 (325)	27	100	43.4	28	100	42	CPr/CPPS	8.9 years	GUPI (total): 23.5	no	no	not mentioned
Li et al., 2016 (326)	42	19	21.21	62	23	21.29	Migraine without aura	>6 months	-	no	no	no
Li et al., 2017 (327)	46	22	21.24	72	21	21.30	Migraine without aura	>6 months	-	no	no	no
Liu et al., 2017 (328)	42	36	22.95	66	36	23.09	FD	35.44 months	NDI: 50.79	no	no	no
Martucci et al., 2015 (329)	45	0	39.4	45	0	37.9	UCPPS	9.42 years	MPQ sensory: 9.34	yes	yes	no
Michels et al., 2016 (330) medication-overuse headache	16	44	42.6	11	17	44.4	MOH	16.6 years	VAS: 4.2	yes	yes	not mentioned
myofascial pain	16	44	42.6	12	27	38.3	MYO	5.5 years	VAS: 5.5	yes		not mentioned
Niddam et al., 2016 (331) migraine without aura	26	35	31.2	26	35	32.3	Migraine without aura	13.5 years	Migraine pain intensity: 8.0	no	yes	not mentioned
migraine with aura		35		26	35	28.3	Migraine with aura	13.1 years	Migraine pain intensity: 8.2	no		not mentioned
Pujol et al., 2014 (332)	36	0	44	40	0	46.4	FM	7.2 years	FIQ-total: 66.2	yes	no	no
Qi et al., 2016 (333)	32	78	27.4	31	81	29.3	IBS	32.67 months	IBS-SSS: 242.48	no	no	no
Truini et al., 2016 (334)	15	13	28-67	20	5	26-65	FM	-	-	no	no	no
Wang et al., 2017 (184)	38	33	55.89	38	42	55.87	TN	7.05 years	VAS: 5.79	yes	no	no
Wei et al., 2016 a (335)	60	0	23.9	56	0	23.1	PDM	9.5 years	PRI (recalled): 35.0	yes	yes	no

Wei et al., 2016 b (336)	49	0	23.8	46	0	23.33	PDM	9.22 years	PRI (recalled): 9.22	yes	yes	no
Wei et al., 2017 (337)	65	0	24.2	61	0	23	PDM	8.95 years	MPQ: 35.0	yes	yes	no
Wei et al., 2019 (338)	23	22	35.67	28	18	36.68	Migraine	8.95 years	VAS: 6.50	no	no	no
Yu et al., 2017 (339)	48	23	35.12	48	23	35.47	Migraine	9.38 years	VAS: 7.23	yes	no	no
Zhang et al., 2017 (340)	31	29	40.2	30	29	41	Migraine	9.6 years	VAS: 7.2	no	no	no
Zhang et al., 2019 (341)	34	38	43.32	29	34	48.14	TN	6.02 years	VAS: 6.31	no	yes	no

Table S8. ANX = anxiety disorder, BMS = Burning mouth syndrome, CBP = Chronic back pain, CLBP = Chronic low back pain, CMP = Chronic musculoskeletal pain, CP = chronic pain, CPr = Chronic prostatitis, CPPS = Chronic pelvic pain syndrome, CRPS = Complex regional pain syndrome, DNP = Diabetic neuropathic pain, FD = Functional dyspepsia, FIQ = Fibromyalgia Impact Questionnaire, FM = Fibromyalgia, GUPI = Genitourinary Pain Index, HC = healthy controls, IBS = irritable bowel syndrome, IBS-SSS = IBS Symptom Severity Score, IC = interstitial cystitis, iFC = intrinsic functional connectivity, MDD = major depressive disorder, MOH = medication-overuse headache, (SF-)MPQ = (Short Form) McGill Pain Questionnaire Pain Rating Index, MYO = chonic myofascial pain, NDI = Nepean Dyspepsia Index, NRS = Numeric rating scale, OA = Osteoarthritis, PBS = Painful bladder syndrome, PDM = Primary dysmenorrhea, PHN = Postherpetic neuralgia, PRI = Pain rating index, RA = Rheumatoid arthritis, SCD = Sickle cell disease, TMD = Temporomandibular disorders, TN = Trigeminal neuralgia, UCPPS = Urologic chronic pelvic pain syndrome, VAS = Visual Analogue Scale.

Table S9: Comorbidity across investigated disorders in the included studies.

Disorder	Included studies	Studies with comorbid						Total number of studies with comorbidity	Studies that did not mention comorbidity
		MDD	ANX	CP	MDD+ANX	MDD+CP	ANX+CP		
**Gray matter volume meta-analysis**									
**Major depressive disorder**	63		7					7	10
**Anxiety disorders**	41	15						15	3
**Chronic pain**	65	5			1			6	15
**Intrinsic functional connectivity meta-analysis**									
**Major depressive disorder**	68		22					22	5
**Anxiety disorders**	41	26						26	4
**Chronic pain**	42				1			1	15

Table S10: Studies with no significant results.

Author, year	HC (n)	Patients (n)
**Gray matter volume meta-analysis**		
**Major depressive disorder**		
Hagan et al., 2015 (342)	36	109
Biedermann et al., 2015 (343)	35	46
Jia et al., 2010 (344)	52	52
Treadway et al., 2009 (345)	19	19
Yoshikawa et al., 2006 (346)	29	22
Colloby et al., 2011 (347)	30	28
Koolschijn et al., 2010 (348)	38	28
**Anxiety disorders**		
Szabó et al., 2015 (349)	60 (trauma-exposed)	75
	60 (non-exposed)	
Moon et al., 2015 (350)	15	15
Jatzko et al., 2006 (351)	15	15
Eckart et al., 2011 (352)	19 (traumatized)	20
	13 (non-traumatized)	
**Chronic pain**		
Yu et al., 2017 (353)	20	25
Chen et al., 2016 (354)	40	40
Hougaard et al., 2015 (355)	60	20
Tessitore et al., 2013 (356)	20	25
Dolman et al., 2014 (357)	14	28
Seminowicz et al., 2013 (358)	23	13
Russo et al., 2012 (359)	14	14
Farmer et al., 2011 (360)	16	19
Hsu et al., 2009 (361)	29	29 (with affective disorders)
	29	29 (without affective disorders)
Celle et al., 2017 (362)	39	25 (episodic migraine)
	39	37 (history of migraine)
Ung et al., 2014 (363)	47	47
**Intrinsic functional connectivity meta-analysis**		
**Major depressive disorder**		
Demenscu et al., 2016 (364)	25	25
**Anxiety disorders**		
Brunetti et al., 2017 (365)	20	20
Olivé et al., 2018 (366)	50	67
		41 (with dissociative subtype)
**Chronic pain**		
Schwedt et al., 2014 (367)	20	38

Table S10. HC = healthy controls.

Table S11: Number of studies, number of patients/controls, and mean age of patients/controls per seed network and disorder.

	LIM	FPN	DMN	SAL	PSM	DAN	VIS
**Total**	**55**<br>1712/1580<br>29.88/29.05	**30**<br>787/727<br>37.43/37.40	**76**<br>2456/2279<br>36.76/36.42	**47**<br>1511/1376<br>35.39/34.95	**18**<br>606/541<br>41.78/39.93	**4**<br>115/86<br>37.54/39.11	**5**<br>158/129<br>36.86/39.11
MDD	**20**<br>695/640<br>29.48/28.85	**15**<br>376/350<br>40.57/41.64	**47**<br>1622/1479<br>36.95/36.69	**13**<br>368/360<br>32.4/30.71	**5**<br>107/111<br>43.15/43.73	**1**<br>23/20<br>33.09/31.1	**2**<br>64/39<br>27.61/28.09
ANX	**29**<br>803/740<br>29.15/27.77	**6**<br>156/163<br>28.92/27.70	**14**<br>403/394<br>28.73/28.32	**10**<br>314/266<br>36.23/35.7	**3**<br>158/95<br>32.98/28.15	**1**<br>28/28<br>32.93/33.21	**0**<br>- / -<br>- / -
CP	**6**<br>214/200<br>35.01/35.99	**9**<br>255/214<br>36.75/36.21	**15**<br>431/406<br>43.21/42.68	**24**<br>829/750<br>36.81/36.95	**10**<br>341/335<br>44.62/41.57	**2**<br>64/38<br>40.56/46.06	**3**<br>94/90<br>46.1/46.47

Table S11. The first line of each cell represents the number of studies using seeds located in the respective seed network, the second line represents the number of patients/controls, and the third line the mean age of patients/controls. ANX = anxiety disorders, CP = chronic pain, DAN = dorsal attention network, DMN = default-mode network, FPN = frontoparietal network, LIM = limbic network, MDD = major depressive disorder, PSM = primary-sensorimotor network, SAL = salience network, VIS = visual network.

Table S12: Gray matter volume changes compared to healthy controls.

Effect anatomy/region	MNI peak coordinates			Voxels	Maximum P (density statistic)	p-Value p_{FWER}
	x	y	z			
**MDD**						
MDD > HC						
R lateral occipital cortex, superior division	46	-70	24	2252	0.105	0.007
MDD < HC						
L inferior frontal gyrus, pars opercularis	-44	16	8	13	0.151	0.007
L frontal operculum cortex	-44	12	6	577	0.135	<0.001
R frontal pole	32	44	26	563	0.131	0.001
L subcallosal cortex	-6	16	-14	3078	0.119	<0.001
R insula	42	-6	-18	1618	0.113	0.017
L central opercular cortex	-46	2	4	839	0.097	0.039
L anterior cingulate cortex	-2	18	12	18807	0.118	<0.001
**ANX**						
ANX > HC						
R lateral occipital cortex, superior division	48	-64	30	1538	0.235	0.031
ANX < HC						
L frontal orbital cortex	-38	22	-10	12	0.280	0.031
L insular cortex	-38	8	-12	2545	0.233	<0.001
L thalamus	-2	-8	-2	428	0.215	0.007
L paracingulate gyrus	-8	48	20	338	0.222	0.021
L pallidum	-16	-8	-4	5633	0.228	<0.001
L temporal pole	-36	22	-32	26	0.133	<0.001
L frontal operculum cortex	-38	28	4	108	0.138	<0.001
L paracingulate gyrus	-2	46	18	1614	0.205	0.005
L Thalamus	0	4	4	14413	0.155	<0.001
**CP**						
CP > HC						
L Amygdala	-22	-6	-16	459	0.273	0.002
R parahippocampal gyrus, posterior division	28	-32	-14	95	0.236	0.010
L Hippocampus	-24	-12	-18	1723	0.186	<0.001

R parahippocampal gyrus, posterior division	28	-36	-12	571	0.186	<0.001
L Hippocampus	-24	-18	-18	1920	0.129	<0.001
R lobus anterior cerebelli	20	-32	-22	6257	0.122	<0.001
L temporal fusiform cortex, anterior division	-32	-4	-42	269	0.075	<0.001
L temporal fusiform cortex, posterior division	-38	-12	-36	49	0.064	<0.001
L brainstem	-10	-36	-18	29	0.068	<0.001
L lingual gyrus	-22	-44	-14	31	0.065	<0.001
CP < HC						
L medial prefrontal cortex	0	58	0	59	0.204	0.017
L paracingulate gyrus	-2	32	34	32	0.186	0.031
R central opercular cortex	44	10	2	943	0.166	<0.001
L frontal orbital cortex	-38	28	-4	290	0.162	0.005
L frontal pole	-2	58	6	1318	0.166	<0.001
L central opercular cortex	-46	-14	14	245	0.150	0.021
L paracingulate gyrus	-2	32	32	1093	0.166	<0.001
R central opercular cortex	44	8	2	1236	0.135	<0.001
L anterior cingulate cortex	0	24	26	3072	0.124	<0.001
L premotor cortex	-44	-4	18	2436	0.147	<0.001
L anterior cingulate cortex	-10	16	28	15774	0.133	<0.001
L frontal pole	-12	70	6	112	0.078	<0.001
L medial prefrontal cortex	0	66	22	10	0.072	<0.001

Table S12. Aberrant gray matter volume in major depressive disorder (MDD), anxiety disorders (ANX), and chronic pain (CP), based on Multilevel Kernel Density Meta-Analysis (p<0.05 FWER-corrected, both height- and extent-based threshold). HC = healthy controls, MNI = Montreal Neurological Institute, P = weighted proportion of studies (=density statistic).

Table S13: Common gray matter volume changes.

Effect anatomy/region	MNI peak coordinates			Voxels	p-Value
	x	y	z		
Common volume increase: (MDD > HC) ∩ (ANX > HC) ∩ (CP > HC)					
-					
Common volume decrease: (MDD < HC) ∩ (ANX < HC) ∩ (CP < HC)					
Bilateral prefrontal cortex	8	50	-22	1464	<0.0015
L insula, superior temporal gyrus, and lateral prefrontal cortex	-56	-8	-14	1191	<0.0015
R lateral and medial supplementary motor area	10	10	40	87	<0.0015
R insula and inferior frontal gyrus	54	6	-4	51	<0.0015
R lateral and medial supplementary motor area	12	28	40	14	<0.0015

Table S13. Common gray matter volume changes in major depressive disorder (MDD), anxiety disorders (ANX), and chronic pain (CP), based on MKDA meta-analysis and subsequent conjunction (p<0.0015). HC = healthy controls, MNI = Montreal Neurological Institute.

Table S14: Pairwise contrasts of gray matter volume changes.

**Effect anatomy/region**	**MNI peak coordinates**			**Voxels**	**p-Value**
	**x**	**y**	**z**		
**MDD**					
MDD > ANX					
Volume increase: (MDD > controls) > (ANX > controls)					
-					
Volume decrease: (MDD < controls) > (ANX < controls)					
L premotor cortex	-26	10	44	208	<0.001
MDD > CP					
Volume increase: (MDD > controls) > (CP > controls)					
-					
Volume decrease: (MDD < controls) > (CP < controls)					
R parahippocampal gyrus	34	-16	-34	5432	<0.001
L superior temporal gyrus	-52	8	-10	1599	<0.001
**ANX**					
ANX > MDD					
Volume increase: (ANX > controls) > (MDD > controls)					
-					
Volume decrease: (ANX < controls) > (MDD < controls)					
L inferior frontal gyrus	-44	28	-12	10463	0.000446
L thalamus	-14	-22	2	1124	<0.001
R parahippocampal gyrus	24	-12	-34	220	<0.001
R temporal pole	32	20	-42	123	<0.001
R amygdala	36	4	-28	68	<0.001
ANX > CP					
Volume increase: (ANX > controls) > (CP > controls)					
-					
Volume decrease: (ANX < controls) > (CP < controls)					
L temporal pole	-46	18	-18	9489	0.00052
R parahippocampal gyrus	38	-14	-38	588	<0.001
R temporal pole	38	20	-40	337	<0.001
**CP**					
CP > MDD					
Volume increase: (CP > controls) > (MDD > controls)					

L insula	-32	-6	-20	5341	0.00173
R fusiform gyrus	24	-30	-18	1520	0.000687
R lobus cerebelli posterior	30	-50	-62	1474	<0.001
R parahippocampal gyrus	30	-8	-36	214	<0.001
R putamen	26	-4	4	25	<0.001
R thalamus	18	-18	2	20	<0.001
Volume decrease: (CP < controls) > (MDD < controls)					
R frontopolar prefrontal cortex	0	66	6	4311	0.0011
R amygdala	32	8	-22	1789	0.0041
L inferior frontal gyrus	-30	40	-12	1424	0.000185
R primary motor cortex	40	-4	36	92	<0.001
L supplementary motor area	-22	28	34	11	<0.001
CP > ANX					
Volume increase: (CP > controls) > (ANX > controls)					
L amygdala	-32	-4	-24	5349	0.00164
R parahippocampal gyrus	36	-30	-14	4245	0.000684
R amygdala	14	-4	-10	31	<0.001
R thalamus	12	0	0	23	<0.001
Volume decrease: (CP < controls) > (ANX < controls)					
R insula	30	10	-16	246	0.00057

Table S14. Pairwise contrasts of gray matter volume changes across major depressive disorder (MDD), anxiety disorders (ANX), and chronic pain (CP), based on MKDA meta-analysis and subsequent conjunction (p<0.005). HC = healthy controls, MNI = Montreal Neurological Institute.

Table S15: Specific gray matter volume changes.

Effect anatomy/region	MNI peak coordinates			Voxels	p-Value
	x	y	z		
**MDD**					
Specific volume increase: (MDD > ANX) ∩ (MDD > CP)					
-					
Specific volume decrease: (MDD > ANX) ∩ (MDD > CP)					
-					
**ANX**					
Specific volume increase: (ANX > MDD) ∩ (ANX > CP)					
-					
Specific volume decrease: (ANX > MDD) ∩ (ANX > CP)					
L inferior temporal gyrus	-48	6	-38	7405	<0.00005
Posterior cingulate cortex	0	-28	10	236	<0.00005
R parahippocampal gyrus	24	-12	-34	117	<0.00005
R temporal pole	36	22	-40	109	<0.00005
R amygdala	36	4	-28	45	<0.00005
R superior temporal gyrus	42	-4	-18	17	<0.00005
**CP**					
Specific volume increase: (CP > MDD) ∩ (CP > ANX)					
L parahippocampal gyrus	-30	-6	-54	4875	<0.00005
R lobus cerebelli posterior	30	-50	-62	1474	<0.00005
R lobus cerebelli anterior	52	-52	-34	1455	<0.00005
R gyrus rectus	10	10	-22	38	<0.00005
R thalamus	18	-18	2	20	<0.00005
Specific volume decrease: (CP > MDD) ∩ (CP > ANX)					
R insula	30	10	-16	210	<0.00005

Table S15. Specific gray matter volume changes in major depressive disorder (MDD), anxiety disorders (ANX), and chronic pain (CP), based on conjunction across pairwise contrasts ($p<0.00005$). MNI = Montreal Neurological Institute.

Table S16: Intrinsic functional connectivity changes compared to healthy controls.

Seed network	Effect network	Effect anatomy/region	MNI peak coordinates			Voxels	Maximum P (density statistic)	p-Value p_{FWER}
			x	y	z			
**MDD**								
MDD > HC								
PSM	DAN	R Lateral Occipital Cortex, superior division	34	-70	30	40	0.779	0.0432
DMN	FPN	L middle frontal gyrus	-44	26	32	60	0.234	0.031
DMN	-	L middle frontal gyrus	-40	28	30	699	0.200	<0.001
DMN	DMN	L precuneous cortex	-2	-66	44	395	0.190	0.029
DMN	-	L middle frontal gyrus	-36	32	28	993	0.132	0.009
MDD < HC								
LIM	DMN	R Insular Cortex	30	14	-12	26	0.311	0.002
LIM	SAL	R Insular Cortex	38	8	-10	811	0.246	0.016
FPN	FPN	R angular gyrus	42	-54	50	608	0.301	0.035
**ANX**								
ANX > HC								
LIM	SAL	R supramarginal gyrus, posterior division	60	-40	24	621	0.304	0.011
LIM	-	L tegmentum mesencephali	-12	-24	-14	8913	0.231	0.004
ANX < HC								
LIM	DMN	R cingulate gyrus, posterior division	6	-44	18	374	0.185	0.041
LIM	-	R orbitofrontal cortex	22	40	-10	5515	0.163	0.040
**CP**								
CP > HC								
SAL	PSM	L postcentral gyrus	-8	-40	60	2427	0.217	0.017
CP < HC								
PSM	SAL	R central opercular cortex	46	-2	4	1077	0.655	0.040
PSM	-	R fusiform gyrus	36	-58	-8	962	0.449	0.046
PSM	PSM	R central opercular cortex	54	-2	8	1138	0.452	<0.001
PSM	PSM	R primary auditory cortex	60	-4	0	1241	0.305	0.001
PSM	-	R insula	34	-12	8	163	0.275	0.001

SAL	DMN	L paracingulate gyrus	-2	46	-8	11	0.330	0.026
SAL	DMN	L paracingulate gyrus	-4	54	4	141	0.329	0.042
SAL	VIS	R cuneal cortex	12	-70	24	10	0.297	0.035
SAL	DMN	L paracingulate gyrus	-4	50	2	1461	0.286	<0.001
SAL	DMN	R precuneous cortex	8	-58	28	913	0.288	0.001
SAL	DMN	R paracingulate gyrus	2	44	26	1953	0.234	<0.001
SAL	FPN	L frontal pole	-22	58	8	28	0.172	<0.001
SAL	VIS	R cuneal cortex	20	-72	22	1008	0.176	<0.001
SAL	-	L medial prefrontal cortex	-14	38	12	11	0.159	<0.001
SAL	DMN	L cingulate gyrus, posterior division	-2	-46	32	688	0.186	<0.001
SAL	DMN	L precuneous cortex	-10	-60	20	7908	0.263	<0.001
SAL	-	R paracingulate gyrus	2	46	16	3176	0.129	0.049
SAL	DAN	R lateral occipital cortex, superior division	44	-64	22	1555	0.134	<0.001
LIM	-	L anterior prefrontal cortex	-12	52	-8	311	0.409	0.059
FPN	PSM	L planum temporale	-58	-36	12	55	0.447	0.042

Table S16. Intrinsic functional connectivity changes in major depressive disorder (MDD), anxiety disorders (ANX), and chronic pain (CP), based on Multilevel Kernel Density Meta-Analysis (p<0.05 FWER-corrected, both height- and extent-based threshold). Labelling of effect networks was derived from the location of the cluster's peak coordinate. - = cluster was located outside of the network mask. DAN = dorsal attention network, DMN = default-mode network, FPN = frontoparietal network, HC = healthy controls, LIM = limbic network, MNI = Montreal Neurological Institute, P = weighted proportion of studies (=density statistic), PSM = primary-sensorimotor network, SAL = salience network, VIS = visual network.

Table S17: Common intrinsic functional connectivity changes.

Effect anatomy/region	MNI peak coordinates			Voxels	p-Value
	x	y	z		
Common hyperconnectivity: (MDD > HC) ∩ (ANX > HC) ∩ (CP > HC)					
-					
Common hypoconnectivity: (MDD < HC) ∩ (ANX < HC) ∩ (CP < HC)					
-					

Table S17. Common intrinsic functional connectivity changes in major depressive disorder (MDD), anxiety disorders (ANX), and chronic pain (CP), based on MKDA meta-analysis and subsequent conjunction ($p<0.0015$). HC = healthy controls, MNI = Montreal Neurological Institute.

Table S18: Pairwise contrasts of intrinsic functional connectivity changes.

Seed network	Effect anatomy/region	MNI peak coordinates			Voxels	p-Value
		x	y	z		
**MDD**						
MDD > ANX						
Hyperconnectivity: (MDD > controls) > (ANX > controls)						
DMN	L frontal cortex (BA9)	-50	24	32	1753	0.000365
DMN	L parietal cortex (BA7)	-4	-66	52	395	0.00138
Hypoconnectivity: (MDD < controls) > (ANX < controls)						
LIM	R globus pallidus	18	4	-4	827	0.000471
MDD > CP						
Hyperconnectivity: (MDD > controls) > (CP > controls)						
PSM	R angular gyrus	36	-76	28	40	0.00186
DMN	L frontal cortex (BA9)	-50	24	32	1562	0.000359
Hypoconnectivity: (MDD < controls) > (CP < controls)						
LIM	R Insula	42	4	-16	788	0.000885
FPN	R angular gyrus	44	-50	44	609	0.001
**ANX**						
ANX > MDD						
Hyperconnectivity: (ANX > controls) > (MDD > controls)						
LIM	R lobus cerebelli posterior	18	-64	-40	1647	0.000145
LIM	R superior temporal gyrus	52	-40	8	621	0.000399
Hypoconnectivity: (ANX < controls) > (MDD < controls)						
LIM	R cingulate gyrus (isthmus/BA30)	12	-44	10	374	0.00159
ANX > CP						
Hyperconnectivity: (ANX > controls) > (CP > controls)						
LIM	R lobus cerebelli posterior	18	-64	-40	2022	0.0018
LIM	R superior temporal gyrus	52	-40	8	621	0.000269
Hypoconnectivity: (ANX < controls) > (CP < controls)						
-						
**CP**						
CP > MDD						

Hyperconnectivity: (CP > controls) > (MDD > controls)						
SAL	R primary motor cortex	8	-26	48	1502	0.00039
Hypoconnectivity: (CP < controls) > (MDD < controls)						
PSM	R insula	44	-4	-8	3622	0.0023
SAL	R primary visual cortex	12	-72	20	6844	0.0012
SAL	L dorsal anterior cingulate area (BA32)	-2	52	-2	5725	0.0017
SAL	L lobus anterior cerebelli	-2	-48	-4	53	<0.001
LIM	L frontopolar prefrontal cortex	-6	54	-16	116	0.0032
FPN	L superior temporal gyrus (BA22)	-52	-40	10	55	0.0015
CP > ANX						
Hyperconnectivity: (CP > controls) > (ANX > controls)						
-						
Hypoconnectivity: (CP < controls) > (ANX < controls)						
PSM	R insula	44	-4	-8	2509	0.00226
PSM	R fusiform gyrus	38	-64	-20	962	0.00222
SAL	R primary visual cortex	12	-72	20	7150	0.0014
SAL	L dorsal anterior cingulate area (BA32)	-6	50	-4	2497	0.0019

Table S18. Pairwise contrasts of intrinsic functional connectivity changes across major depressive disorder (MDD), anxiety disorders (ANX), and chronic pain (CP), based on MKDA meta-analysis and subsequent conjunction (p<0.005). DMN = default-mode network, FPN = frontoparietal network, HC = healthy controls, LIM = limbic network, MNI = Montreal Neurological Institute, PSM = primary-sensorimotor network, SAL = salience network.

Table S19: Specific intrinsic functional connectivity changes.

Seed network	Effect anatomy/region	MNI peak coordinates			Voxels	p-Value
		x	y	z		
**MDD**						
Specific hyperconnectivity: (MDD > ANX) ∩ (MDD > CP)						
DMN	L anterior middle frontal gyrus	-40	40	2	1562	<0.00005
Specific hypoconnectivity: (MDD > ANX) ∩ (MDD > CP)						
LIM	R parahippocampal gyrus	30	-2	-30	781	<0.00005
**ANX**						
Specific hyperconnectivity: (ANX > MDD) ∩ (ANX > CP)						
LIM	R lobus cerebelli posterior	18	-64	-40	1541	<0.00005
LIM	R superior temporal gyrus	52	-40	8	621	<0.00005
Specific hypoconnectivity: (ANX > MDD) ∩ (ANX > CP)						
-						
**CP**						
Specific hyperconnectivity: (CP > MDD) ∩ (CP > ANX)						
-						
Specific hypoconnectivity: (CP > MDD) ∩ (CP > ANX)						
PSM	R middle temporal cortex (BA21)	60	-20	-12	2509	<0.00005
SAL	R primary visual cortex	18	-66	8	4886	<0.00005
SAL	L orbitofrontal cortex	-2	44	-22	2386	<0.00005
SAL	R secondary visual cortex	22	-46	-2	170	<0.00005

Table S19. Specific intrinsic functional connectivity changes in major depressive disorder (MDD), anxiety disorders (ANX), and chronic pain (CP), based on conjunction across pairwise contrasts (p<0.00005). DMN = default-mode network, LIM = limbic network, MNI = Montreal Neurological Institute, PSM = primary-sensorimotor network, SAL = salience network.

Table S20: Regional overlap between specific GMV-changes and specific iFC-changes.

Seed network	Effect anatomy/region	MNI peak coordinates			Voxels	p-Value
		x	y	z		
**MDD**						
-						
**ANX**						
-						
**CP**						
Specific GMV-decrease and specific hypoconnectivity						
PSM	Right ventrolateral prefrontal cortex	48	20	10	18	$<3x10^{-9}$

Table S20. Results are based on inter-modality conjunction ($p<3x10^{-9}$). ANX = anxiety disorders, CP = chronic pain, GMV = gray matter volume, MDD = major depressive disorder, MNI = Montreal Neurological Institute, PSM = primary-sensorimotor network.

Table S21: Noise correction methods (additional to global signal regression) implemented in iFC studies.

Author, year	Noise correction method(s)
Major depressive disorder	
Alalade et al., 2011 (193)	high-pass and temporal low-pass filtering
Alexopoulos et al., 2012 (194)	temporal band-pass filtering
Andreescu et al., 2013 (195)	not reported
Avery et al., 2014 (196)	not reported
Bai et al., 2018 (197)	temporal band-pass filtering, regression of WM, CSF, 24 head-motion parameters (Friston)
Berman et al., 2011 (198)	high-pass and low-pass filtering; detrending
Bessette et al., 2018 (199)	temporal band-pass filtering, regression of WM and CSF, time series detrending
Cao et al., 2012 (200)	removal of linear trend, temporal band-pass filtering, reduce low-frequency drift
Chen et al., 2018 (201)	temporal band-pass filtering, regression of WM, CSF, 24 head-motion parameters (Friston)
Connolly et al., 2013 (202)	temporal band-pass filtering, regression of WM and CSF
Connolly et al., 2017 (203)	temporal band-pass filtering, regression of WM and CSF, linear regression
Crowther et al.,2015 (204)	temporal band-pass filtering
Cullen et al., 2014 (205)	regression of WM, CSF and six motion parameters
Davey et al., 2012 (206)	high-pass filtering to remove low-frequency drifts, linear regression
DelDonno et al., 2017 (207)	temporal band-pass filtering, regression of WM and CSF
Deng et al., 2016 (208)	temporal band-pass filtering to reduce low-frequency drifts, detrending
de Kwaasteniet et al., 2015 (209)	temporal band-pass filtering, regression of WM, CSF and head movement
Ellard et al., 2018 (210)	not reported
Evans et al., 2018 (211)	temporal band-pass filtering, motion censoring, physiological noise correction
Furman et al., 2011 (212)	temporal band-pass filtering, regression of WM, CSF and six motion parameters
Gabbay et al., 2013 (213)	temporal band-pass filtering, linear and quadratic detrending
Gandelman et al., 2018 (214)	temporal band-pass filtering, regression of WM, CSF, realignment parameters
Gong et al., 2017 (215)	temporal band-pass filtering
Guo et al., 2013 a, b (216)<br>treatment-resistant depression	temporal band-pass filtering to reduce low-frequency drift, regression of WM, CSF and six motion parameters
treatment-sensitive depression	
Guo et al., 2013 c, d (217)	temporal band-pass filtering, linear detrending, regression of WM, CSF ROI, six motion parameters
Guo et al., 2015 a, b (218)	temporal band-pass filtering, linear detrending, regression of WM, ventricular ROI, six motion parameters
Jacobs et al., 2016 (219)	temporal band-pass filtering, regression of WM and CSF
Kenny et al., 2010 (220)	temporal high-pass filtering
Kerestes et al., 2015 (221)	linear regression of six motion parameters, high-pass filtering to remove low-frequency drifts
Kim et al., 2016 (222)	temporal band-pass filtering, linear detrending to reduce low-frequency drifts
Li et al., 2015 (223)	temporal band-pass filtering, regression of WM, CSF, six motion vectors
Liu et al., 2018 (224)	Linear detrending, temporal band-pass filtering

Lois et al., 2016 (225)	temporal band-pass filtering, regression of WM, CSF, six parameters
Lui et al., 2011 (226)<br>nonrefractory depression	temporal band-pass filtering, linear detrending, regression of WM and CSF
refractory depression	
Ma et al., 2012 (227)<br>treatment-resistant depression	temporal band-pass filtering to reduce low-frequency drifts
treatment-sensitive depression	
Pannekoek et al., 2014 (228)	temporal high-pass filtering
Peng et al., 2015 (229)	temporal band-pass filtering
Peng et al., 2018 (230)	temporal band-pass filtering, regression of WM, CSF and head-motion
Penner et al., 2016 (231)	temporal band-pass filtering
Peters et al., 2016 (232)	not reported
Philippi et al., 2018 (233)	regression of WM, CSF, six motion parameters
Poeppl et al. 2016 (234)	temporal band-pass filtering
Rao et al., 2016 (235)	not reported
Renner et al., 2017 (236)	not reported
Sawaya et al., 2015 (237)	temporal band-pass filtering, high-pass filtering
Scheinost et al., 2018 (238)	linear and quadratic drifts, regression of WM, CSF, six motion parameters
Schilbach et al., 2016 (239)	temporal band-pass filtering, regression of six motion parameters
Sheline et al., 2010 (240)	temporal band-pass filtering, regression of six parameters
Shu et al., 2014 (241)	temporal band-pass filtering, linear detrending, regression of WM, CSF, six motion parameters
Späti et al., 2015 (242)	temporal band-pass filtering, regression of 24 parameters
Straub et al., 2015 (243)	not reported
Tadayonnejad et al., 2016 (244)	temporal band-pass filtering, regression of WM and CSF
Tahmasian et al., 2013 (245)	temporal band-pass filtering
van Tol et al., 2014 (246)	not reported
Wang et al., 2018 (247)	temporal band-pass filtering, linear detrending, regression of 24-parameter (Friston)
Workman et al., 2016 (248)	temporal band-pass filtering, regression of WM, CSF, 24 motion parameters
Workman et al., 2017 (249)<br>recurring episode	temporal band-pass filtering, regression of WM, CSF, 24 motion parameters
Resilient	
Wu H et al., 2016 (250)	temporal band-pass filtering, regression of WM, CSF, six motion parameters
Wu X et al., 2016 (251)	temporal band-pass filtering to reduce low-frequency drift, linear regression of WM, CSF and six motion parameters
Yang et al., 2016 a (85)	temporal band-pass filtering, regression of WM, CSF, motion-relevant parameters
Yang et al., 2016 b (252)	temporal band-pass filtering, linear detrending, regression of WM and CSF
Yang et al., 2017 (253)	temporal band-pass filtering to reduce low-frequency drift, regression of WM, CSF, six motion parameters
Ye et al., 2012 (254)	temporal band-pass filtering, linear detrending
Ye et al., 2017 (255)<br>young-adult	temporal band-pass filtering
old-adult	
Yin et al., 2015 a (256)	temporal band-pass filtering, linear detrending
Yin et al., 2015 b (257)	temporal band-pass filtering, linear detrending
Yuan et al., 2014 (258)	temporal band-pass filtering, regression of WM, CSF, six motion parameters

Zhang et al., 2015 (259)<br>deficiency syndrome	temporal band-pass filtering
excess syndrome	
Anxiety disorders	
Aghajani et al., 2016 (260)	temporal band-pass filtering, regression of WM and CSF
Andreescu et al., 2014 (261)	regression of motion and CSF
Arnold Anteraper et al., 2014 (262)	temporal band-pass filtering, regression of WM and CSF
Birn et al., 2014 (263)	not reported
Bluhm et al., 2009 (264)	temporal band-pass filtering
Brown et al., 2014 (265)	temporal band-pass filtering, regression of WM and CSF
Chen et al., 2018 (266)<br>trauma-exposed control	temporal band-pass filtering to remove low-frequency drifts, regression of WM, CSF and head motion parameters
healthy control	
Cui et al., 2017 (267)	temporal band-pass filtering, linear detrending, regression of WM, CSF, six motion parameters
Dodhia et al., 2014 (268)	regression of WM, CSF and movement parameters
Dorfman et al., 2016 (269)	temporal band-pass filtering, regression of WM and 12 motion parameters, cubic detrending
Geiger et al., 2016 (270)	temporal band-pass filtering
Hahn et al., 2011 (271)	temporal band-pass filtering, linear regression of WM and CSF
Hamm et al., 2014 (272)	removal of non-neuronal sources of noise
Harricharan et al., 2016 (273)<br>non-dissociative subtype	temporal band-pass filtering
dissociative subtype	
Harricharan et al., 2017 (274)<br>non-dissociative subtype	temporal band-pass filtering
dissociative subtype	
Holmes et al., 2018 (275)	regression of WM, CSF, 24-parameters motion model
Jung et al., 2018 (276)	removing physiological noise
Li et al., 2016 (277)	temporal band-pass filtering, linear detrending
Liao et al., 2011 (278)	temporal band-pass filtering, regression of WM and CSF
Liu et al., 2015 (279)	temporal band-pass filtering, linear regression of WM, CSF, six motion parameters
Makovac et al., 2015 (280)	not reported
Manning et al., 2015 (281)	temporal band-pass filtering, linear regression of WM and CSF
Misaki et al., 2017 (282)	regression of WM, CSF, 12 motion parameters
Nicholson et al., 2015 (283)<br>non-dissociative subtype	temporal band-pass filtering
dissociative subtype	
Pace-Schott et al., 2017 (284)	temporal band-pass filtering, linear regression of WM, CSF, six parameters
Pannekoek et al., 2013 a (285)	temporal high-pass filtering
Pannekoek et al., 2013 b (286)	temporal high-pass filtering
Patriat et al., 2016 (287)	temporal band-pass filtering, motion censoring
Prater et al., 2013 (288)	temporal band-pass filtering, regression of six motion parameters
Rabellino et al., 2018 (289)<br>non-dissociative subtype	temporal band-pass filtering, regression of six motion parameters
dissociative subtype	
Rabinak et al., 2011 (290)	temporal band-pass filtering, regression of WM, CSF, six movement parameters
Roy et al., 2013 (291)	not reported
Sripada et al., 2012 a, b (292,293)	temporal band-pass filtering

Thome et al., 2017 (294)	temporal band-pass filtering
Toazza et al., 2016 (295)	temporal band-pass filtering, regression of WM, CSF, six motion parameters
Wang et al., 2016 (296)	decreasing spatial noise
Yuan et al., 2016 (297)	temporal band-pass filtering, linear and quadratic detrending, regression of WM, CSF, six motion parameters
Yuan et al., 2017 a (298)	temporal band-pass filtering
Yuan et al., 2017 b (299)	temporal band-pass filtering, linear detrending, regression of WM, CSF, 24 parameters mode (Friston)
Zhang et al., 2016 (300)	temporal band-pass filtering, linear detrending, regression of WM, CSF, six movement parameters
Zhu et al., 2017 (301)	temporal band-pass filtering, linear regression of WM, CSF, six parameters
Chronic pain	
Ayoub et al., 2019 (302)	temporal band-pass filtering, regression of WM, CSF, six dimensions
Baliki et al., 2011 (303)	temporal high-pass filtering, linear regression
Bhatt et al., 2019 (304)	temporal band-pass filtering, regression of WM, CSF, six parameters
Bolwerk et al., 2013 (305)	not reported
Case et al., 2017 (306)	temporal band-pass filtering, motion correction
Cauda et al., 2009 (307)	temporal band-pass filtering, regression of WM, CSF, six motion parameters
Ceko et al., 2013 (137)<br>older subjects	temporal band-pass filtering, regression of WM, CSF, six motion parameters
younger subjects	
Ceko et al., 2015 (308)	temporal band-pass filtering, regression of WM, CSF, six motion parameters
Chen et al., 2016 (309)<br>episodic mirgaine	temporal band-pass filtering, linear detrending
chronic migraine	
Cottam et al., 2018 (310)	regression of WM, CSF, six motion parameters
Fallon et al., 2016 (311)	temporal band-pass filtering
Flodin et al., 2014 (312)	temporal band/high-pass filtering, regression of WM and CSF
Flodin et al., 2016 (313)	temporal band-pass filtering, regression of WM and CSF
Han et al., 2013 (314)	temporal band-pass filtering
He et al., 2018 (315)	temporal band-pass filtering, linear detrending, scrubbing regression
Hong et al., 2014 (316)<br>Male	temporal band-pass filtering, regression of WM, CSF, six parameters
Female	
Ichesco et al., 2012 (317)	temporal band-pass filtering, removing linear drift, regression of WM, CSF and realignment parameters
Ichesco et al., 2014 (318)	temporal band-pass filtering, removing linear drift, regression of WM, CSF and realignment parameters
Jiang et al., 2016 (319)	temporal band-pass filtering, linear detrending, regression of WM, CSF, six motion parameters
Khan et al., 2014 (320)	temporal band-pass filtering, regression of WM, CSF, six motion parameters
Kilpatrik et al., 2014 (321)	temporal band-pass filtering, regression of WM, CSF, six motion parameters
Kim et al., 2017 (322)	temporal band-pass filtering, regression of WM, CSF, six motion parameters
Kong et al., 2018 (323)	temporal band-pass filtering

Kucyi et al., 2014 (324)	temporal band-pass filtering, regression of WM, CSF, six motion parameters
Kutch et al., 2015 (325)	temporal high-pass filtering, regression of WM, CSF, six motion parameters
Li et al., 2016 (326)	temporal band-pass filtering
Li et al., 2017 (327)	temporal band-pass filtering, linear detrending
Liu et al., 2017 (328)	temporal band-pass filtering, detrending
Martucci et al., 2015 (329)	temporal high-pass filtering, regression of WM and CSF
Michels et al., 2016 (330)<br>medication-overuse headache	removing all non-neuronal noise
myofascial pain	
Niddam et al., 2016 (331)<br>migraine without aura	temporal band-pass filtering, linear detrending, regression of WM, CSF, six motion parameters
migraine with aura	
Pujol et al., 2014 (332)	temporal high-pass filtering, drift removal, regression of WM and CSF
Qi et al., 2016 (333)	temporal band-pass filtering, regression of WM, CSF, six motion parameters
Truini et al., 2016 (334)	not reported
Wang et al., 2017 (184)	temporal band-pass filtering
Wei et al., 2016 a (335)	temporal band-pass filtering, regression of WM, CSF, six movement parameters, linear detrending
Wei et al., 2016 b (336)	temporal band-pass filtering, regression of WM, CSF, six movement parameters, linear detrending
Wei et al., 2017 (337)	temporal band-pass filtering, regression of WM, CSF, six movement parameters, linear detrending
Wei et al., 2019 (338)	temporal band-pass filtering, detrending
Yu et al., 2017 (339)	temporal band-pass filtering, regression of WM and CSF
Zhang et al., 2017 (340)	temporal band-pass filtering
Zhang et al., 2019 (341)	temporal band-pass filtering

Table S21: CSF = cerebrospinal fluid, WM = white matter.

Supplementary References

1. Stroup DF, Berlin JA, Morton SC, Olkin I, Williamson GD, Rennie D, et al. (2000): Meta-analysis of observational studies in epidemiology: a proposal for reporting. Meta-analysis Of Observational Studies in Epidemiology (MOOSE) group. *JAMA*. 283:2008-2012.
2. Brandl F, Avram M, Weise B, Shang J, Simoes B, Bertram T, et al. (2019): Specific Substantial Dysconnectivity in Schizophrenia: A Transdiagnostic Multimodal Meta-analysis of Resting-State Functional and Structural Magnetic Resonance Imaging Studies. *Biol Psychiatry*. 85:573-583.
3. Kaiser RH, Andrews-Hanna JR, Wager TD, Pizzagalli DA (2015): Large-Scale Network Dysfunction in Major Depressive Disorder: A Meta-analysis of Resting-State Functional Connectivity. *JAMA Psychiatry*. 72:603-611.
4. Buckner RL, Krienen FM, Castellanos A, Diaz JC, Yeo BT (2011): The organization of the human cerebellum estimated by intrinsic functional connectivity. *J Neurophysiol*. 106:2322-2345.
5. Choi EY, Yeo BT, Buckner RL (2012): The organization of the human striatum estimated by intrinsic functional connectivity. *J Neurophysiol*. 108:2242-2263.
6. Yeo BT, Krienen FM, Sepulcre J, Sabuncu MR, Lashkari D, Hollinshead M, et al. (2011): The organization of the human cerebral cortex estimated by intrinsic functional connectivity. *J Neurophysiol*. 106:1125-1165.
7. Xu J, Van Dam NT, Feng C, Luo Y, Ai H, Gu R, et al. (2019): Anxious brain networks: A coordinate-based activation likelihood estimation meta-analysis of resting-state functional connectivity studies in anxiety. *Neurosci Biobehav Rev*. 96:21-30.
8. Wager TD, Lindquist M, Kaplan L (2007): Meta-analysis of functional neuroimaging data: current and future directions. *Soc Cogn Affect Neurosci*. 2:150-158.
9. Radua J, Romeo M, Mataix-Cols D, Fusar-Poli P (2013): A general approach for combining voxel-based meta-analyses conducted in different neuroimaging modalities. *Curr Med Chem*. 20:462-466.
10. Brandl F, Le Houcq Corbi Z, Mulej Bratec S, Sorg C (2019): Cognitive reward control recruits medial and lateral frontal cortices, which are also involved in cognitive emotion regulation: A coordinate-based meta-analysis of fMRI studies. *Neuroimage*. 200:659-673.
11. Etkin A, Wager TD (2007): Functional neuroimaging of anxiety: a meta-analysis of emotional processing in PTSD, social anxiety disorder, and specific phobia. *Am J Psychiatry*. 164:1476-1488.
12. Albajes-Eizagirre A, Solanes A, Radua J (2019): Meta-analysis of non-statistically significant unreported effects. *Stat Methods Med Res*. 28:3741-3754.
13. Albajes-Eizagirre A, Solanes A, Vieta E, Radua J (2019): Voxel-based meta-analysis via permutation of subject images (PSI): Theory and implementation for SDM. *Neuroimage*. 186:174-184.
14. Bora E, Fornito A, Pantelis C, Yücel M (2012): Gray matter abnormalities in Major Depressive Disorder: a meta-analysis of voxel based morphometry studies. *J Affect Disord*. 138:9-18.
15. Arnone D, Job D, Selvaraj S, Abe O, Amico F, Cheng Y, et al. (2016): Computational meta-analysis of statistical parametric maps in major depression. *Hum Brain Mapp*. 37:1393-1404.
16. Wise T, Radua J, Via E, Cardoner N, Abe O, Adams TM, et al. (2017): Common and distinct patterns of grey-matter volume alteration in major depression and bipolar disorder: evidence from voxel-based meta-analysis. *Mol Psychiatry*. 22:1455-1463.
17. Radua J, van den Heuvel OA, Surguladze S, Mataix-Cols D (2010): Meta-analytical comparison of voxel-based morphometry studies in obsessive-compulsive disorder vs other anxiety disorders. *Arch Gen Psychiatry*. 67:701-711.
18. Kühn S, Gallinat J (2013): Gray matter correlates of posttraumatic stress disorder: a quantitative meta-analysis. *Biol Psychiatry*. 73:70-74.

19. Shang J, Fu Y, Ren Z, Zhang T, Du M, Gong Q, et al. (2014): The common traits of the ACC and PFC in anxiety disorders in the DSM-5: meta-analysis of voxel-based morphometry studies. *PLoS One*. 9:e93432.
20. Smallwood RF, Laird AR, Ramage AE, Parkinson AL, Lewis J, Clauw DJ, et al. (2013): Structural brain anomalies and chronic pain: a quantitative meta-analysis of gray matter volume. *J Pain*. 14:663-675.
21. Cauda F, Palermo S, Costa T, Torta R, Duca S, Vercelli U, et al. (2014): Gray matter alterations in chronic pain: A network-oriented meta-analytic approach. *Neuroimage Clin*. 4:676-686.
22. Tatu K, Costa T, Nani A, Diano M, Quarta DG, Duca S, et al. (2018): How do morphological alterations caused by chronic pain distribute across the brain? A meta-analytic co-alteration study. *Neuroimage Clin*. 18:15-30.
23. Ploner M, Sorg C, Gross J (2017): Brain Rhythms of Pain. *Trends Cogn Sci*. 21:100-110.
24. Baliki MN, Apkarian AV (2015): Nociception, Pain, Negative Moods, and Behavior Selection. *Neuron*. 87:474-491.
25. Kuner R, Flor H (2017): Structural plasticity and reorganisation in chronic pain. *Nat Rev Neurosci*. 18:113.
26. Gu Z, Gu L, Eils R, Schlesner M, Brors B (2014): circlize implements and enhances circular visualization in R. *Bioinformatics*. 30:2811-2812.
27. Ahn SJ, Kyeong S, Suh SH, Kim JJ, Chung TS, Seok JH (2016): What is the impact of child abuse on gray matter abnormalities in individuals with major depressive disorder: a case control study. *BMC Psychiatry*. 16:397.
28. Amico F, Meisenzahl E, Koutsouleris N, Reiser M, Möller HJ, Frodl T (2011): Structural MRI correlates for vulnerability and resilience to major depressive disorder. *J Psychiatry Neurosci*. 36:15-22.
29. Arnone D, McKie S, Elliott R, Juhasz G, Thomas EJ, Downey D, et al. (2013): State-dependent changes in hippocampal grey matter in depression. *Mol Psychiatry*. 18:1265-1272.
30. Cai Y, Liu J, Zhang L, Liao M, Zhang Y, Wang L, et al. (2015): Grey matter volume abnormalities in patients with bipolar I depressive disorder and unipolar depressive disorder: a voxel-based morphometry study. *Neurosci Bull*. 31:4-12.
31. Chen Z, Peng W, Sun H, Kuang W, Li W, Jia Z, et al. (2016): High-field magnetic resonance imaging of structural alterations in first-episode, drug-naive patients with major depressive disorder. *Transl Psychiatry*. 6:e942.
32. Cheng YQ, Xu J, Chai P, Li HJ, Luo CR, Yang T, et al. (2010): Brain volume alteration and the correlations with the clinical characteristics in drug-naïve first-episode MDD patients: a voxel-based morphometry study. *Neurosci Lett*. 480:30-34.
33. Depping MS, Wolf ND, Vasic N, Sambataro F, Thomann PA, Christian Wolf R (2015): Specificity of abnormal brain volume in major depressive disorder: a comparison with borderline personality disorder. *J Affect Disord*. 174:650-657.
34. Egger K, Schocke M, Weiss E, Auffinger S, Esterhammer R, Goebel G, et al. (2008): Pattern of brain atrophy in elderly patients with depression revealed by voxel-based morphometry. *Psychiatry Res*. 164:237-244.
35. Fang J, Mao N, Jiang X, Li X, Wang B, Wang Q (2015): Functional and Anatomical Brain Abnormalities and Effects of Antidepressant in Major Depressive Disorder: Combined Application of Voxel-Based Morphometry and Amplitude of Frequency Fluctuation in Resting State. *J Comput Assist Tomogr*. 39:766-773.
36. Frodl T, Koutsouleris N, Bottlender R, Born C, Jäger M, Mörgenthaler M, et al. (2008): Reduced gray matter brain volumes are associated with variants of the serotonin transporter gene in major depression. *Mol Psychiatry*. 13:1093-1101.
37. Grieve SM, Korgaonkar MS, Koslow SH, Gordon E, Williams LM (2013): Widespread reductions in gray matter volume in depression. *Neuroimage Clin*. 3:332-339.

38. Guo W, Liu F, Yu M, Zhang J, Zhang Z, Liu J, et al. (2014): Functional and anatomical brain deficits in drug-naive major depressive disorder. *Prog Neuropsychopharmacol Biol Psychiatry*. 54:1-6.
39. Harada K, Matsuo K, Nakashima M, Hobara T, Higuchi N, Higuchi F, et al. (2016): Disrupted orbitomedial prefrontal limbic network in individuals with later-life depression. *J Affect Disord*. 204:112-119.
40. Hwang JP, Lee TW, Tsai SJ, Chen TJ, Yang CH, Lirng JF, et al. (2010): Cortical and subcortical abnormalities in late-onset depression with history of suicide attempts investigated with MRI and voxel-based morphometry. *J Geriatr Psychiatry Neurol*. 23:171-184.
41. Igata N, Kakeda S, Watanabe K, Ide S, Kishi T, Abe O, et al. (2017): Voxel-based morphometric brain comparison between healthy subjects and major depressive disorder patients in Japanese with the s/s genotype of 5-HTTLPR. *Sci Rep*. 7:3931.
42. Kim MJ, Hamilton JP, Gotlib IH (2008): Reduced caudate gray matter volume in women with major depressive disorder. *Psychiatry Res*. 164:114-122.
43. Klauser P, Fornito A, Lorenzetti V, Davey CG, Dwyer DB, Allen NB, et al. (2015): Cortico-limbic network abnormalities in individuals with current and past major depressive disorder. *J Affect Disord*. 173:45-52.
44. Kong L, Wu F, Tang Y, Ren L, Kong D, Liu Y, et al. (2014): Frontal-subcortical volumetric deficits in single episode, medication-naïve depressed patients and the effects of 8 weeks fluoxetine treatment: a VBM-DARTEL study. *PLoS One*. 9:e79055.
45. Lai CH, Hsu YY, Wu YT (2010): First episode drug-naïve major depressive disorder with panic disorder: gray matter deficits in limbic and default network structures. *Eur Neuropsychopharmacol*. 20:676-682.
46. Lai CH, Wu YT (2014): Frontal-insula gray matter deficits in first-episode medication-naïve patients with major depressive disorder. *J Affect Disord*. 160:74-79.
47. Lai CH, Wu YT (2015): The gray matter alterations in major depressive disorder and panic disorder: Putative differences in the pathogenesis. *J Affect Disord*. 186:1-6.
48. Lan MJ, Chhetry BT, Liston C, Mann JJ, Dubin M (2016): Transcranial Magnetic Stimulation of Left Dorsolateral Prefrontal Cortex Induces Brain Morphological Changes in Regions Associated with a Treatment Resistant Major Depressive Episode: An Exploratory Analysis. *Brain Stimul*. 9:577-583.
49. Lee HY, Tae WS, Yoon HK, Lee BT, Paik JW, Son KR, et al. (2011): Demonstration of decreased gray matter concentration in the midbrain encompassing the dorsal raphe nucleus and the limbic subcortical regions in major depressive disorder: an optimized voxel-based morphometry study. *J Affect Disord*. 133:128-136.
50. Leung KK, Lee TM, Wong MM, Li LS, Yip PS, Khong PL (2009): Neural correlates of attention biases of people with major depressive disorder: a voxel-based morphometric study. *Psychol Med*. 39:1097-1106.
51. Machino A, Kunisato Y, Matsumoto T, Yoshimura S, Ueda K, Yamawaki Y, et al. (2014): Possible involvement of rumination in gray matter abnormalities in persistent symptoms of major depression: an exploratory magnetic resonance imaging voxel-based morphometry study. *J Affect Disord*. 168:229-235.
52. Matsubara T, Matsuo K, Harada K, Nakano M, Nakashima M, Watanuki T, et al. (2016): Distinct and Shared Endophenotypes of Neural Substrates in Bipolar and Major Depressive Disorders. *PLoS One*. 11:e0168493.
53. Matsuo K, Harada K, Fujita Y, Okamoto Y, Ota M, Narita H, et al. (2019): Distinctive Neuroanatomical Substrates for Depression in Bipolar Disorder versus Major Depressive Disorder. *Cereb Cortex*. 29:202-214.
54. Modinos G, Allen P, Frascarelli M, Tognin S, Valmaggia L, Xenaki L, et al. (2014): Are we really mapping psychosis risk? Neuroanatomical signature of affective disorders in subjects at ultra high risk. *Psychol Med*. 44:3491-3501.

55. Nakano M, Matsuo K, Nakashima M, Matsubara T, Harada K, Egashira K, et al. (2014): Gray matter volume and rapid decision-making in major depressive disorder. *Prog Neuropsychopharmacol Biol Psychiatry*. 48:51-56.
56. Opel N, Zwanzger P, Redlich R, Grotegerd D, Dohm K, Arolt V, et al. (2016): Differing brain structural correlates of familial and environmental risk for major depressive disorder revealed by a combined VBM/pattern recognition approach. *Psychol Med*. 46:277-290.
57. Ozalay O, Aksoy B, Tunay S, Simsek F, Chandhoke S, Kitis O, et al. (2016): Cortical thickness and VBM in young women at risk for familial depression and their depressed mothers with positive family history. *Psychiatry Res Neuroimaging*. 252:1-9.
58. Peng J, Liu J, Nie B, Li Y, Shan B, Wang G, et al. (2011): Cerebral and cerebellar gray matter reduction in first-episode patients with major depressive disorder: a voxel-based morphometry study. *Eur J Radiol*. 80:395-399.
59. Peng H, Wu K, Li J, Qi H, Guo S, Chi M, et al. (2014): Increased suicide attempts in young depressed patients with abnormal temporal-parietal-limbic gray matter volume. *J Affect Disord*. 165:69-73.
60. Qi H, Ning Y, Li J, Guo S, Chi M, Gao M, et al. (2014): Gray matter volume abnormalities in depressive patients with and without anxiety disorders. *Medicine (Baltimore)*. 93:e345.
61. Qiu H, Li X, Zhao W, Du L, Huang P, Fu Y, et al. (2016): Electroconvulsive Therapy-Induced Brain Structural and Functional Changes in Major Depressive Disorders: A Longitudinal Study. *Med Sci Monit*. 22:4577-4586.
62. Redlich R, Almeida JJ, Grotegerd D, Opel N, Kugel H, Heindel W, et al. (2014): Brain morphometric biomarkers distinguishing unipolar and bipolar depression. A voxel-based morphometry-pattern classification approach. *JAMA Psychiatry*. 71:1222-1230.
63. Ries ML, Wichmann A, Bendlin BB, Johnson SC (2009): Posterior Cingulate and Lateral Parietal Gray Matter Volume in Older Adults with Depressive Symptoms. *Brain Imaging Behav*. 3:233-239.
64. Salvadore G, Nugent AC, Lemaitre H, Luckenbaugh DA, Tinsley R, Cannon DM, et al. (2011): Prefrontal cortical abnormalities in currently depressed versus currently remitted patients with major depressive disorder. *Neuroimage*. 54:2643-2651.
65. Scheuerecker J, Meisenzahl EM, Koutsouleris N, Roesner M, Schöpf V, Linn J, et al. (2010): Orbitofrontal volume reductions during emotion recognition in patients with major depression. *J Psychiatry Neurosci*. 35:311-320.
66. Serra-Blasco M, Portella MJ, Gómez-Ansón B, de Diego-Adeliño J, Vives-Gilabert Y, Puigdemont D, et al. (2013): Effects of illness duration and treatment resistance on grey matter abnormalities in major depression. *Br J Psychiatry*. 202:434-440.
67. Shad MU, Muddasani S, Rao U (2012): Gray matter differences between healthy and depressed adolescents: a voxel-based morphometry study. *J Child Adolesc Psychopharmacol*. 22:190-197.
68. Shah PJ, Ebmeier KP, Glabus MF, Goodwin GM (1998): Cortical grey matter reductions associated with treatment-resistant chronic unipolar depression. Controlled magnetic resonance imaging study. *Br J Psychiatry*. 172:527-532.
69. Shen Z, Cheng Y, Yang S, Dai N, Ye J, Liu X, et al. (2016): Changes of grey matter volume in first-episode drug-naive adult major depressive disorder patients with different age-onset. *Neuroimage Clin*. 12:492-498.
70. Soriano-Mas C, Hernández-Ribas R, Pujol J, Urretavizcaya M, Deus J, Harrison BJ, et al. (2011): Cross-sectional and longitudinal assessment of structural brain alterations in melancholic depression. *Biol Psychiatry*. 69:318-325.
71. Sprengelmeyer R, Steele JD, Mwangi B, Kumar P, Christmas D, Milders M, et al. (2011): The insular cortex and the neuroanatomy of major depression. *J Affect Disord*. 133:120-127.
72. Stratmann M, Konrad C, Kugel H, Krug A, Schöning S, Ohrmann P, et al. (2014): Insular and hippocampal gray matter volume reductions in patients with major depressive disorder. *PLoS One*. 9:e102692.

73. Ueda I, Kakeda S, Watanabe K, Yoshimura R, Kishi T, Abe O, et al. (2016): Relationship between G1287A of the NET Gene Polymorphisms and Brain Volume in Major Depressive Disorder: A Voxel-Based MRI Study. *PLoS One*. 11:e0150712.
74. van Tol MJ, van der Wee NJ, van den Heuvel OA, Nielen MM, Demenescu LR, Aleman A, et al. (2010): Regional brain volume in depression and anxiety disorders. *Arch Gen Psychiatry*. 67:1002-1011.
75. Vasic N, Walter H, Höse A, Wolf RC (2008): Gray matter reduction associated with psychopathology and cognitive dysfunction in unipolar depression: a voxel-based morphometry study. *J Affect Disord*. 109:107-116.
76. Vasic N, Wolf ND, Grön G, Sosic-Vasic Z, Connemann BJ, Sambataro F, et al. (2015): Baseline brain perfusion and brain structure in patients with major depression: a multimodal magnetic resonance imaging study. *J Psychiatry Neurosci*. 40:412-421.
77. Wagner G, Koch K, Schachtzabel C, Reichenbach JR, Sauer H, Schlösser Md RG (2008): Enhanced rostral anterior cingulate cortex activation during cognitive control is related to orbitofrontal volume reduction in unipolar depression. *J Psychiatry Neurosci*. 33:199-208.
78. Wagner G, Koch K, Schachtzabel C, Schultz CC, Sauer H, Schlösser RG (2011): Structural brain alterations in patients with major depressive disorder and high risk for suicide: evidence for a distinct neurobiological entity? *Neuroimage*. 54:1607-1614.
79. Wang L, Dai W, Su Y, Wang G, Tan Y, Jin Z, et al. (2012): Amplitude of low-frequency oscillations in first-episode, treatment-naive patients with major depressive disorder: a resting-state functional MRI study. *PLoS One*. 7:e48658.
80. Wang L, Wang T, Liu S, Liang Z, Meng Y, Xiong X, et al. (2014): Cerebral anatomical changes in female asthma patients with and without depression compared to healthy controls and patients with depression. *J Asthma*. 51:927-933.
81. Wang YL, Yang SZ, Sun WL, Shi YZ, Duan HF (2016): Altered functional interaction hub between affective network and cognitive control network in patients with major depressive disorder. *Behav Brain Res*. 298:301-309.
82. Watanabe K, Kakeda S, Yoshimura R, Abe O, Ide S, Hayashi K, et al. (2015): Relationship between the catechol-O-methyl transferase Val108/158Met genotype and brain volume in treatment-naive major depressive disorder: Voxel-based morphometry analysis. *Psychiatry Res*. 233:481-487.
83. Wehry AM, McNamara RK, Adler CM, Eliassen JC, Croarkin P, Cerullo MA, et al. (2015): Neurostructural impact of co-occurring anxiety in pediatric patients with major depressive disorder: a voxel-based morphometry study. *J Affect Disord*. 171:54-59.
84. Yang X, Ma X, Huang B, Sun G, Zhao L, Lin D, et al. (2015): Gray matter volume abnormalities were associated with sustained attention in unmedicated major depression. *Compr Psychiatry*. 63:71-79.
85. Yang J, Yin Y, Svob C, Long J, He X, Zhang Y, et al. (2017): Amygdala Atrophy and Its Functional Disconnection with the Cortico-Striatal-Pallidal-Thalamic Circuit in Major Depressive Disorder in Females. *PLoS One*. 12:e0168239.
86. Yuan Y, Zhu W, Zhang Z, Bai F, Yu H, Shi Y, et al. (2008): Regional gray matter changes are associated with cognitive deficits in remitted geriatric depression: an optimized voxel-based morphometry study. *Biol Psychiatry*. 64:541-544.
87. Zhang X, Yao S, Zhu X, Wang X, Zhu X, Zhong M (2012): Gray matter volume abnormalities in individuals with cognitive vulnerability to depression: a voxel-based morphometry study. *J Affect Disord*. 136:443-452.
88. Zhao Y, Chen L, Zhang W, Xiao Y, Shah C, Zhu H, et al. (2017): Gray Matter Abnormalities in Non-comorbid Medication-naive Patients with Major Depressive Disorder or Social Anxiety Disorder. *EBioMedicine*. 21:228-235.
89. Zou K, Deng W, Li T, Zhang B, Jiang L, Huang C, et al. (2010): Changes of brain morphometry in first-episode, drug-naïve, non-late-life adult patients with major depression: an optimized voxel-based morphometry study. *Biol Psychiatry*. 67:186-188.

90. Ahmed F, Spottiswoode BS, Carey PD, Stein DJ, Seedat S (2012): Relationship between neurocognition and regional brain volumes in traumatized adolescents with and without posttraumatic stress disorder. *Neuropsychobiology*. 66:174-184.
91. Asami T, Yamasue H, Hayano F, Nakamura M, Uehara K, Otsuka T, et al. (2009): Sexually dimorphic gray matter volume reduction in patients with panic disorder. *Psychiatry Res*. 173:128-134.
92. Bossini L, Santarnecchi E, Casolaro I, Koukouna D, Caterini C, Cecchini F, et al. (2017): Morphovolumetric changes after EMDR treatment in drug-naïve PTSD patients. *Riv Psichiatr*. 52:24-31.
93. Carrion VG, Weems CF, Watson C, Eliez S, Menon V, Reiss AL (2009): Converging evidence for abnormalities of the prefrontal cortex and evaluation of midsagittal structures in pediatric posttraumatic stress disorder: an MRI study. *Psychiatry Res*. 172:226-234.
94. Chen S, Xia W, Li L, Liu J, He Z, Zhang Z, et al. (2006): Gray matter density reduction in the insula in fire survivors with posttraumatic stress disorder: a voxel-based morphometric study. *Psychiatry Res*. 146:65-72.
95. Chen Y, Fu K, Feng C, Tang L, Zhang J, Huan Y, et al. (2012): Different regional gray matter loss in recent onset PTSD and non PTSD after a single prolonged trauma exposure. *PLoS One*. 7:e48298.
96. Cheng B, Huang X, Li S, Hu X, Luo Y, Wang X, et al. (2015): Gray Matter Alterations in Post-Traumatic Stress Disorder, Obsessive-Compulsive Disorder, and Social Anxiety Disorder. *Front Behav Neurosci*. 9:219.
97. Corbo V, Clément MH, Armony JL, Pruessner JC, Brunet A (2005): Size versus shape differences: contrasting voxel-based and volumetric analyses of the anterior cingulate cortex in individuals with acute posttraumatic stress disorder. *Biol Psychiatry*. 58:119-124.
98. Frick A, Engman J, Alaie I, Björkstrand J, Faria V, Gingnell M, et al. (2014): Enlargement of visual processing regions in social anxiety disorder is related to symptom severity. *Neurosci Lett*. 583:114-119.
99. Gold AL, Brotman MA, Adleman NE, Lever SN, Steuber ER, Fromm SJ, et al. (2016): Comparing Brain Morphometry Across Multiple Childhood Psychiatric Disorders. *J Am Acad Child Adolesc Psychiatry*. 55:1027-1037.e1023.
100. Hakamata Y, Matsuoka Y, Inagaki M, Nagamine M, Hara E, Imoto S, et al. (2007): Structure of orbitofrontal cortex and its longitudinal course in cancer-related post-traumatic stress disorder. *Neurosci Res*. 59:383-389.
101. Herringa R, Phillips M, Almeida J, Insana S, Germain A (2012): Post-traumatic stress symptoms correlate with smaller subgenual cingulate, caudate, and insula volumes in unmedicated combat veterans. *Psychiatry Res*. 203:139-145.
102. Hilbert K, Pine DS, Muehlhan M, Lueken U, Steudte-Schmiedgen S, Beesdo-Baum K (2015): Gray and white matter volume abnormalities in generalized anxiety disorder by categorical and dimensional characterization. *Psychiatry Res*. 234:314-320.
103. Irle E, Barke A, Lange C, Ruhleder M (2014): Parietal abnormalities are related to avoidance in social anxiety disorder: a study using voxel-based morphometry and manual volumetry. *Psychiatry Res*. 224:175-183.
104. Kasai K, Yamasue H, Gilbertson MW, Shenton ME, Rauch SL, Pitman RK (2008): Evidence for acquired pregenual anterior cingulate gray matter loss from a twin study of combat-related posttraumatic stress disorder. *Biol Psychiatry*. 63:550-556.
105. Lai CH, Wu YT (2012): Fronto-temporo-insula gray matter alterations of first-episode, drug-naïve and very late-onset panic disorder patients. *J Affect Disord*. 140:285-291.
106. Liao M, Yang F, Zhang Y, He Z, Song M, Jiang T, et al. (2013): Childhood maltreatment is associated with larger left thalamic gray matter volume in adolescents with generalized anxiety disorder. *PLoS One*. 8:e71898.

107. Makovac E, Meeten F, Watson DR, Garfinkel SN, Critchley HD, Ottaviani C (2016): Neurostructural abnormalities associated with axes of emotion dysregulation in generalized anxiety. *Neuroimage Clin*. 10:172-181.
108. Meng Y, Lui S, Qiu C, Qiu L, Lama S, Huang X, et al. (2013): Neuroanatomical deficits in drug-naïve adult patients with generalized social anxiety disorder: a voxel-based morphometry study. *Psychiatry Res*. 214:9-15.
109. Moon CM, Kim GW, Jeong GW (2014): Whole-brain gray matter volume abnormalities in patients with generalized anxiety disorder: voxel-based morphometry. *Neuroreport*. 25:184-189.
110. Moon CM, Yang JC, Jeong GW (2015): Explicit verbal memory impairments associated with brain functional deficits and morphological alterations in patients with generalized anxiety disorder. *J Affect Disord*. 186:328-336.
111. Moon CM, Jeong GW (2017): Abnormalities in gray and white matter volumes associated with explicit memory dysfunction in patients with generalized anxiety disorder. *Acta Radiol*. 58:353-361.
112. Na KS, Ham BJ, Lee MS, Kim L, Kim YK, Lee HJ, et al. (2013): Decreased gray matter volume of the medial orbitofrontal cortex in panic disorder with agoraphobia: a preliminary study. *Prog Neuropsychopharmacol Biol Psychiatry*. 45:195-200.
113. Nardo D, Högberg G, Looi JC, Larsson S, Hällström T, Pagani M (2010): Gray matter density in limbic and paralimbic cortices is associated with trauma load and EMDR outcome in PTSD patients. *J Psychiatr Res*. 44:477-485.
114. Nardo D, Högberg G, Lanius RA, Jacobsson H, Jonsson C, Hällström T, et al. (2013): Gray matter volume alterations related to trait dissociation in PTSD and traumatized controls. *Acta Psychiatr Scand*. 128:222-233.
115. O'Doherty DCM, Tickell A, Ryder W, Chan C, Hermens DF, Bennett MR, et al. (2017): Frontal and subcortical grey matter reductions in PTSD. *Psychiatry Res Neuroimaging*. 266:1-9.
116. Rocha-Rego V, Pereira MG, Oliveira L, Mendlowicz MV, Fiszman A, Marques-Portella C, et al. (2012): Decreased premotor cortex volume in victims of urban violence with posttraumatic stress disorder. *PLoS One*. 7:e42560.
117. Schienle A, Ebner F, Schäfer A (2011): Localized gray matter volume abnormalities in generalized anxiety disorder. *Eur Arch Psychiatry Clin Neurosci*. 261:303-307.
118. Strawn JR, Wehry AM, Chu WJ, Adler CM, Eliassen JC, Cerullo MA, et al. (2013): Neuroanatomic abnormalities in adolescents with generalized anxiety disorder: a voxel-based morphometry study. *Depress Anxiety*. 30:842-848.
119. Strawn JR, Hamm L, Fitzgerald DA, Fitzgerald KD, Monk CS, Phan KL (2015): Neurostructural abnormalities in pediatric anxiety disorders. *J Anxiety Disord*. 32:81-88.
120. Sui SG, Wu MX, King ME, Zhang Y, Ling L, Xu JM, et al. (2010): Abnormal grey matter in victims of rape with PTSD in Mainland China: a voxel-based morphometry study. *Acta Neuropsychiatr*. 22:118-126.
121. Talati A, Pantazatos SP, Schneier FR, Weissman MM, Hirsch J (2013): Gray matter abnormalities in social anxiety disorder: primary, replication, and specificity studies. *Biol Psychiatry*. 73:75-84.
122. Tavanti M, Battaglini M, Borgogni F, Bossini L, Calossi S, Marino D, et al. (2012): Evidence of diffuse damage in frontal and occipital cortex in the brain of patients with post-traumatic stress disorder. *Neurol Sci*. 33:59-68.
123. Thomaes K, Dorrepaal E, Draijer N, de Ruiter MB, van Balkom AJ, Smit JH, et al. (2010): Reduced anterior cingulate and orbitofrontal volumes in child abuse-related complex PTSD. *J Clin Psychiatry*. 71:1636-1644.
124. Tükel R, Aydın K, Yüksel Ç, Ertekin E, Koyuncu A, Taş C (2015): Gray matter abnormalities in patients with social anxiety disorder: A voxel-based morphometry study. *Psychiatry Res*. 234:106-112.

125. Yamasue H, Kasai K, Iwanami A, Ohtani T, Yamada H, Abe O, et al. (2003): Voxel-based analysis of MRI reveals anterior cingulate gray-matter volume reduction in posttraumatic stress disorder due to terrorism. *Proc Natl Acad Sci U S A*. 100:9039-9043.
126. Yoo HK, Kim MJ, Kim SJ, Sung YH, Sim ME, Lee YS, et al. (2005): Putaminal gray matter volume decrease in panic disorder: an optimized voxel-based morphometry study. *Eur J Neurosci*. 22:2089-2094.
127. Zhang J, Tan Q, Yin H, Zhang X, Huan Y, Tang L, et al. (2011): Decreased gray matter volume in the left hippocampus and bilateral calcarine cortex in coal mine flood disaster survivors with recent onset PTSD. *Psychiatry Res*. 192:84-90.
128. Absinta M, Rocca MA, Colombo B, Falini A, Comi G, Filippi M (2012): Selective decreased grey matter volume of the pain-matrix network in cluster headache. *Cephalalgia*. 32:109-115.
129. Apkarian AV, Sosa Y, Sonty S, Levy RM, Harden RN, Parrish TB, et al. (2004): Chronic back pain is associated with decreased prefrontal and thalamic gray matter density. *J Neurosci*. 24:10410-10415.
130. Arkink EB, Schmitz N, Schoonman GG, van Vliet JA, Haan J, van Buchem MA, et al. (2017): The anterior hypothalamus in cluster headache. *Cephalalgia*. 37:1039-1050.
131. As-Sanie S, Harris RE, Napadow V, Kim J, Neshewat G, Kairys A, et al. (2012): Changes in regional gray matter volume in women with chronic pelvic pain: a voxel-based morphometry study. *Pain*. 153:1006-1014.
132. Baliki MN, Schnitzer TJ, Bauer WR, Apkarian AV (2011): Brain morphological signatures for chronic pain. *PLoS One*. 6:e26010.
133. Barad MJ, Ueno T, Younger J, Chatterjee N, Mackey S (2014): Complex regional pain syndrome is associated with structural abnormalities in pain-related regions of the human brain. *J Pain*. 15:197-203.
134. Bishop JH, Shpaner M, Kubicki A, Clements S, Watts R, Naylor MR (2018): Structural network differences in chronic muskuloskeletal pain: Beyond fractional anisotropy. *Neuroimage*. 182:441-455.
135. Burgmer M, Gaubitz M, Konrad C, Wrenger M, Hilgart S, Heuft G, et al. (2009): Decreased gray matter volumes in the cingulo-frontal cortex and the amygdala in patients with fibromyalgia. *Psychosom Med*. 71:566-573.
136. Buckalew N, Haut MW, Morrow L, Weiner D (2008): Chronic pain is associated with brain volume loss in older adults: preliminary evidence. *Pain Med*. 9:240-248.
137. Ceko M, Bushnell MC, Fitzcharles MA, Schweinhardt P (2013): Fibromyalgia interacts with age to change the brain. *Neuroimage Clin*. 3:249-260.
138. Davis KD, Pope G, Chen J, Kwan CL, Crawley AP, Diamant NE (2008): Cortical thinning in IBS: implications for homeostatic, attention, and pain processing. *Neurology*. 70:153-154.
139. Diaz-Piedra C, Guzman MA, Buela-Casal G, Catena A (2016): The impact of fibromyalgia symptoms on brain morphometry. *Brain Imaging Behav*. 10:1184-1197.
140. Fallon N, Alghamdi J, Chiu Y, Sluming V, Nurmikko T, Stancak A (2013): Structural alterations in brainstem of fibromyalgia syndrome patients correlate with sensitivity to mechanical pressure. *Neuroimage Clin*. 3:163-170.
141. Fritz HC, McAuley JH, Wittfeld K, Hegenscheid K, Schmidt CO, Langner S, et al. (2016): Chronic Back Pain Is Associated With Decreased Prefrontal and Anterior Insular Gray Matter: Results From a Population-Based Cohort Study. *J Pain*. 17:111-118.
142. Geha PY, Baliki MN, Harden RN, Bauer WR, Parrish TB, Apkarian AV (2008): The brain in chronic CRPS pain: abnormal gray-white matter interactions in emotional and autonomic regions. *Neuron*. 60:570-581.
143. Gerstner G, Ichesco E, Quintero A, Schmidt-Wilcke T (2011): Changes in regional gray and white matter volume in patients with myofascial-type temporomandibular disorders: a voxel-based morphometry study. *J Orofac Pain*. 25:99-106.

144. Gustin SM, Peck CC, Wilcox SL, Nash PG, Murray GM, Henderson LA (2011): Different pain, different brain: thalamic anatomy in neuropathic and non-neuropathic chronic pain syndromes. *J Neurosci*. 31:5956-5964.
145. Gwilym SE, Filippini N, Douaud G, Carr AJ, Tracey I (2010): Thalamic atrophy associated with painful osteoarthritis of the hip is reversible after arthroplasty: a longitudinal voxel-based morphometric study. *Arthritis Rheum*. 62:2930-2940.
146. Holle D, Naegel S, Krebs S, Gaul C, Gizewski E, Diener HC, et al. (2011): Hypothalamic gray matter volume loss in hypnic headache. *Ann Neurol*. 69:533-539.
147. Ivo R, Nicklas A, Dargel J, Sobottke R, Delank KS, Eysel P, et al. (2013): Brain structural and psychometric alterations in chronic low back pain. *Eur Spine J*. 22:1958-1964.
148. Keltner JR, Connolly CG, Vaida F, Jenkinson M, Fennema-Notestine C, Archibald S, et al. (2017): HIV Distal Neuropathic Pain Is Associated with Smaller Ventral Posterior Cingulate Cortex. *Pain Med*. 18:428-440.
149. Kim JH, Suh SI, Seol HY, Oh K, Seo WK, Yu SW, et al. (2008): Regional grey matter changes in patients with migraine: a voxel-based morphometry study. *Cephalalgia*. 28:598-604.
150. Kuchinad A, Schweinhardt P, Seminowicz DA, Wood PB, Chizh BA, Bushnell MC (2007): Accelerated brain gray matter loss in fibromyalgia patients: premature aging of the brain? *J Neurosci*. 27:4004-4007.
151. Lewis GN, Parker RS, Sharma S, Rice DA, McNair PJ (2018): Structural Brain Alterations Before and After Total Knee Arthroplasty: A Longitudinal Assessment. *Pain Med*. 19:2166-2176.
152. Li T, Zhang S, Kurata J (2018): Suppressed descending pain modulatory and enhanced sensorimotor networks in patients with chronic low back pain. *J Anesth*. 32:831-843.
153. Liao X, Mao C, Wang Y, Zhang Q, Cao D, Seminowicz DA, et al. (2018): Brain gray matter alterations in Chinese patients with chronic knee osteoarthritis pain based on voxel-based morphometry. *Medicine (Baltimore)*. 97:e0145.
154. Liu J, Lan L, Mu J, Zhao L, Yuan K, Zhang Y, et al. (2015): Genetic contribution of catechol-O-methyltransferase in hippocampal structural and functional changes of female migraine sufferers. *Hum Brain Mapp*. 36:1782-1795.
155. Liu J, Mu J, Liu Q, Dun W, Zhang M, Tian J (2017): Brain structural properties predict psychologically mediated hypoalgesia in an 8-week sham acupuncture treatment for migraine. *Hum Brain Mapp*. 38:4386-4397.
156. Luchtmann M, Steinecke Y, Baecke S, Lützkendorf R, Bernarding J, Kohl J, et al. (2014): Structural brain alterations in patients with lumbar disc herniation: a preliminary study. *PLoS One*. 9:e90816.
157. Marciszewski KK, Meylakh N, Di Pietro F, Macefield VG, Macey PM, Henderson LA (2018): Altered brainstem anatomy in migraine. *Cephalalgia*. 38:476-486.
158. Martikainen IK, Peciña M, Love TM, Nuechterlein EB, Cummiford CM, Green CR, et al. (2013): Alterations in endogenous opioid functional measures in chronic back pain. *J Neurosci*. 33:14729-14737.
159. Mehnert J, May A (2019): Functional and structural alterations in the migraine cerebellum. *J Cereb Blood Flow Metab*. 39:730-739.
160. Mole TB, MacIver K, Sluming V, Ridgway GR, Nurmikko TJ (2014): Specific brain morphometric changes in spinal cord injury with and without neuropathic pain. *Neuroimage Clin*. 5:28-35.
161. Naegel S, Holle D, Desmarattes N, Theysohn N, Diener HC, Katsarava Z, et al. (2014): Cortical plasticity in episodic and chronic cluster headache. *Neuroimage Clin*. 6:415-423.
162. Neeb L, Bastian K, Villringer K, Israel H, Reuter U, Fiebach JB (2017): Structural Gray Matter Alterations in Chronic Migraine: Implications for a Progressive Disease? *Headache*. 57:400-416.
163. Obermann M, Rodriguez-Raecke R, Naegel S, Holle D, Mueller D, Yoon MS, et al. (2013): Gray matter volume reduction reflects chronic pain in trigeminal neuralgia. *Neuroimage*. 74:352-358.

164. Obermann M, Wurthmann S, Steinberg BS, Theysohn N, Diener HC, Naegel S (2014): Central vestibular system modulation in vestibular migraine. *Cephalalgia*. 34:1053-1061.
165. Pleger B, Draganski B, Schwenkreis P, Lenz M, Nicolas V, Maier C, et al. (2014): Complex regional pain syndrome type I affects brain structure in prefrontal and motor cortex. *PLoS One*. 9:e85372.
166. Pomares FB, Funck T, Feier NA, Roy S, Daigle-Martel A, Ceko M, et al. (2017): Histological Underpinnings of Grey Matter Changes in Fibromyalgia Investigated Using Multimodal Brain Imaging. *J Neurosci*. 37:1090-1101.
167. Riederer F, Marti M, Luechinger R, Lanzenberger R, von Meyenburg J, Gantenbein AR, et al. (2012): Grey matter changes associated with medication-overuse headache: correlations with disease related disability and anxiety. *World J Biol Psychiatry*. 13:517-525.
168. Rocca MA, Ceccarelli A, Falini A, Colombo B, Tortorella P, Bernasconi L, et al. (2006): Brain gray matter changes in migraine patients with T2-visible lesions: a 3-T MRI study. *Stroke*. 37:1765-1770.
169. Rocca MA, Messina R, Colombo B, Falini A, Comi G, Filippi M (2014): Structural brain MRI abnormalities in pediatric patients with migraine. *J Neurol*. 261:350-357.
170. Rodriguez-Raecke R, Niemeier A, Ihle K, Ruether W, May A (2009): Brain gray matter decrease in chronic pain is the consequence and not the cause of pain. *J Neurosci*. 29:13746-13750.
171. Ruscheweyh R, Deppe M, Lohmann H, Stehling C, Flöel A, Ringelstein BE, et al. (2011): Pain is associated with regional grey matter reduction in the general population. *Pain*. 152:904-911.
172. Russell MD, Barrick TR, Howe FA, Sofat N (2018): Reduced anterior cingulate grey matter volume in painful hand osteoarthritis. *Rheumatol Int*. 38:1429-1435.
173. Schmidt-Wilcke T, Leinisch E, Gänssbauer S, Draganski B, Bogdahn U, Altmeppen J, et al. (2006): Affective components and intensity of pain correlate with structural differences in gray matter in chronic back pain patients. *Pain*. 125:89-97.
174. Schmidt-Wilcke T, Luerding R, Weigand T, Jürgens T, Schuierer G, Leinisch E, et al. (2007): Striatal grey matter increase in patients suffering from fibromyalgia--a voxel-based morphometry study. *Pain*. 132 Suppl 1:S109-s116.
175. Schmidt-Wilcke T, Gänssbauer S, Neuner T, Bogdahn U, May A (2008): Subtle grey matter changes between migraine patients and healthy controls. *Cephalalgia*. 28:1-4.
176. Schmidt-Wilcke T, Hierlmeier S, Leinisch E (2010): Altered regional brain morphology in patients with chronic facial pain. *Headache*. 50:1278-1285.
177. Schmitz N, Admiraal-Behloul F, Arkink EB, Kruit MC, Schoonman GG, Ferrari MD, et al. (2008): Attack frequency and disease duration as indicators for brain damage in migraine. *Headache*. 48:1044-1055.
178. Seminowicz DA, Labus JS, Bueller JA, Tillisch K, Naliboff BD, Bushnell MC, et al. (2010): Regional gray matter density changes in brains of patients with irritable bowel syndrome. *Gastroenterology*. 139:48-57.e42.
179. Shokouhi M, Clarke C, Morley-Forster P, Moulin DE, Davis KD, St Lawrence K (2018): Structural and Functional Brain Changes at Early and Late Stages of Complex Regional Pain Syndrome. *J Pain*. 19:146-157.
180. Tu CH, Niddam DM, Chao HT, Chen LF, Chen YS, Wu YT, et al. (2010): Brain morphological changes associated with cyclic menstrual pain. *Pain*. 150:462-468.
181. Valet M, Gündel H, Sprenger T, Sorg C, Mühlau M, Zimmer C, et al. (2009): Patients with pain disorder show gray-matter loss in pain-processing structures: a voxel-based morphometric study. *Psychosom Med*. 71:49-56.
182. Valfrè W, Rainero I, Bergui M, Pinessi L (2008): Voxel-based morphometry reveals gray matter abnormalities in migraine. *Headache*. 48:109-117.
183. Vartiainen N, Kallio-Laine K, Hlushchuk Y, Kirveskari E, Seppänen M, Autti H, et al. (2009): Changes in brain function and morphology in patients with recurring herpes simplex virus infections and chronic pain. *Pain*. 144:200-208.

184. Wang Y, Cao DY, Remeniuk B, Krimmel S, Seminowicz DA, Zhang M (2017): Altered brain structure and function associated with sensory and affective components of classic trigeminal neuralgia. *Pain*. 158:1561-1570.
185. Wilcox SL, Gustin SM, Macey PM, Peck CC, Murray GM, Henderson LA (2015): Anatomical changes within the medullary dorsal horn in chronic temporomandibular disorder pain. *Neuroimage*. 117:258-266.
186. Wilcox SL, Gustin SM, Macey PM, Peck CC, Murray GM, Henderson LA (2015): Anatomical changes at the level of the primary synapse in neuropathic pain: evidence from the spinal trigeminal nucleus. *J Neurosci*. 35:2508-2515.
187. Wood PB, Glabus MF, Simpson R, Patterson JC, 2nd (2009): Changes in gray matter density in fibromyalgia: correlation with dopamine metabolism. *J Pain*. 10:609-618.
188. Yang FC, Chou KH, Fuh JL, Huang CC, Lirng JF, Lin YY, et al. (2013): Altered gray matter volume in the frontal pain modulation network in patients with cluster headache. *Pain*. 154:801-807.
189. Yoon EJ, Kim YK, Shin HI, Lee Y, Kim SE (2013): Cortical and white matter alterations in patients with neuropathic pain after spinal cord injury. *Brain Res*. 1540:64-73.
190. Younger JW, Shen YF, Goddard G, Mackey SC (2010): Chronic myofascial temporomandibular pain is associated with neural abnormalities in the trigeminal and limbic systems. *Pain*. 149:222-228.
191. Zhang J, Wu YL, Su J, Yao Q, Wang M, Li GF, et al. (2017): Assessment of gray and white matter structural alterations in migraineurs without aura. *J Headache Pain*. 18:74.
192. Zhang Y, Mao Z, Pan L, Ling Z, Liu X, Zhang J, et al. (2018): Dysregulation of Pain- and Emotion-Related Networks in Trigeminal Neuralgia. *Front Hum Neurosci*. 12:107.
193. Alalade E, Denny K, Potter G, Steffens D, Wang L (2011): Altered cerebellar-cerebral functional connectivity in geriatric depression. *PLoS One*. 6:e20035.
194. Alexopoulos GS, Hoptman MJ, Kanellopoulos D, Murphy CF, Lim KO, Gunning FM (2012): Functional connectivity in the cognitive control network and the default mode network in late-life depression. *J Affect Disord*. 139:56-65.
195. Andreescu C, Tudorascu DL, Butters MA, Tamburo E, Patel M, Price J, et al. (2013): Resting state functional connectivity and treatment response in late-life depression. *Psychiatry Res*. 214:313-321.
196. Avery JA, Drevets WC, Moseman SE, Bodurka J, Barcalow JC, Simmons WK (2014): Major depressive disorder is associated with abnormal interoceptive activity and functional connectivity in the insula. *Biol Psychiatry*. 76:258-266.
197. Bai T, Zu M, Chen Y, Xie W, Cai C, Wei Q, et al. (2018): Decreased Connection Between Reward Systems and Paralimbic Cortex in Depressive Patients. *Front Neurosci*. 12:462.
198. Berman MG, Peltier S, Nee DE, Kross E, Deldin PJ, Jonides J (2011): Depression, rumination and the default network. *Soc Cogn Affect Neurosci*. 6:548-555.
199. Bessette KL, Jenkins LM, Skerrett KA, Gowins JR, DelDonno SR, Zubieta JK, et al. (2018): Reliability, Convergent Validity and Time Invariance of Default Mode Network Deviations in Early Adult Major Depressive Disorder. *Front Psychiatry*. 9:244.
200. Cao X, Liu Z, Xu C, Li J, Gao Q, Sun N, et al. (2012): Disrupted resting-state functional connectivity of the hippocampus in medication-naïve patients with major depressive disorder. *J Affect Disord*. 141:194-203.
201. Chen L, Wang Y, Niu C, Zhong S, Hu H, Chen P, et al. (2018): Common and distinct abnormal frontal-limbic system structural and functional patterns in patients with major depression and bipolar disorder. *Neuroimage Clin*. 20:42-50.
202. Connolly CG, Wu J, Ho TC, Hoeft F, Wolkowitz O, Eisendrath S, et al. (2013): Resting-state functional connectivity of subgenual anterior cingulate cortex in depressed adolescents. *Biol Psychiatry*. 74:898-907.
203. Connolly CG, Ho TC, Blom EH, LeWinn KZ, Sacchet MD, Tymofiyeva O, et al. (2017): Resting-state functional connectivity of the amygdala and longitudinal changes in depression severity in adolescent depression. *J Affect Disord*. 207:86-94.

204. Crowther A, Smoski MJ, Minkel J, Moore T, Gibbs D, Petty C, et al. (2015): Resting-state connectivity predictors of response to psychotherapy in major depressive disorder. *Neuropsychopharmacology*. 40:1659-1673.
205. Cullen KR, Westlund MK, Klimes-Dougan B, Mueller BA, Houri A, Eberly LE, et al. (2014): Abnormal amygdala resting-state functional connectivity in adolescent depression. *JAMA Psychiatry*. 71:1138-1147.
206. Davey CG, Harrison BJ, Yücel M, Allen NB (2012): Regionally specific alterations in functional connectivity of the anterior cingulate cortex in major depressive disorder. *Psychol Med*. 42:2071-2081.
207. DelDonno SR, Jenkins LM, Crane NA, Nusslock R, Ryan KA, Shankman SA, et al. (2017): Affective traits and history of depression are related to ventral striatum connectivity. *J Affect Disord*. 221:72-80.
208. Deng D, Liao H, Duan G, Liu Y, He Q, Liu H, et al. (2016): Modulation of the Default Mode Network in First-Episode, Drug-Naïve Major Depressive Disorder via Acupuncture at Baihui (GV20) Acupoint. *Front Hum Neurosci*. 10:230.
209. de Kwaasteniet BP, Rive MM, Ruhé HG, Schene AH, Veltman DJ, Fellinger L, et al. (2015): Decreased Resting-State Connectivity between Neurocognitive Networks in Treatment Resistant Depression. *Front Psychiatry*. 6:28.
210. Ellard KK, Zimmerman JP, Kaur N, Van Dijk KRA, Roffman JL, Nierenberg AA, et al. (2018): Functional Connectivity Between Anterior Insula and Key Nodes of Frontoparietal Executive Control and Salience Networks Distinguish Bipolar Depression From Unipolar Depression and Healthy Control Subjects. *Biol Psychiatry Cogn Neurosci Neuroimaging*. 3:473-484.
211. Evans JW, Szczepanik J, Brutsché N, Park LT, Nugent AC, Zarate CA, Jr. (2018): Default Mode Connectivity in Major Depressive Disorder Measured Up to 10 Days After Ketamine Administration. *Biol Psychiatry*. 84:582-590.
212. Furman DJ, Hamilton JP, Gotlib IH (2011): Frontostriatal functional connectivity in major depressive disorder. *Biol Mood Anxiety Disord*. 1:11.
213. Gabbay V, Ely BA, Li Q, Bangaru SD, Panzer AM, Alonso CM, et al. (2013): Striatum-based circuitry of adolescent depression and anhedonia. *J Am Acad Child Adolesc Psychiatry*. 52:628-641.e613.
214. Gandelman JA, Albert K, Boyd BD, Park JW, Riddle M, Woodward ND, et al. (2019): Intrinsic Functional Network Connectivity Is Associated With Clinical Symptoms and Cognition in Late-Life Depression. *Biol Psychiatry Cogn Neurosci Neuroimaging*. 4:160-170.
215. Gong L, Yin Y, He C, Ye Q, Bai F, Yuan Y, et al. (2017): Disrupted reward circuits is associated with cognitive deficits and depression severity in major depressive disorder. *J Psychiatr Res*. 84:9-17.
216. Guo W, Liu F, Xue Z, Gao K, Liu Z, Xiao C, et al. (2013): Abnormal resting-state cerebellar-cerebral functional connectivity in treatment-resistant depression and treatment sensitive depression. *Prog Neuropsychopharmacol Biol Psychiatry*. 44:51-57.
217. Guo W, Liu F, Dai Y, Jiang M, Zhang J, Yu L, et al. (2013): Decreased interhemispheric resting-state functional connectivity in first-episode, drug-naive major depressive disorder. *Prog Neuropsychopharmacol Biol Psychiatry*. 41:24-29.
218. Guo W, Liu F, Liu J, Yu M, Zhang Z, Liu G, et al. (2015): Increased cerebellar-default-mode-network connectivity in drug-naive major depressive disorder at rest. *Medicine (Baltimore)*. 94:e560.
219. Jacobs RH, Barba A, Gowins JR, Klumpp H, Jenkins LM, Mickey BJ, et al. (2016): Decoupling of the amygdala to other salience network regions in adolescent-onset recurrent major depressive disorder. *Psychol Med*. 46:1055-1067.
220. Kenny ER, O'Brien JT, Cousins DA, Richardson J, Thomas AJ, Firbank MJ, et al. (2010): Functional connectivity in late-life depression using resting-state functional magnetic resonance imaging. *Am J Geriatr Psychiatry*. 18:643-651.

221. Kerestes R, Harrison BJ, Dandash O, Stephanou K, Whittle S, Pujol J, et al. (2015): Specific functional connectivity alterations of the dorsal striatum in young people with depression. *Neuroimage Clin*. 7:266-272.

222. Kim SM, Park SY, Kim YI, Son YD, Chung US, Min KJ, et al. (2016): Affective network and default mode network in depressive adolescents with disruptive behaviors. *Neuropsychiatr Dis Treat*. 12:49-56.

223. Li W, Ward BD, Xie C, Jones JL, Antuono PG, Li SJ, et al. (2015): Amygdala network dysfunction in late-life depression phenotypes: Relationships with symptom dimensions. *J Psychiatr Res*. 70:121-129.

224. Liu R, Yue Y, Hou Z, Yuan Y, Wang Q (2018): Risk factors associated with cognitions for late-onset depression based on anterior and posterior default mode sub-networks. *J Affect Disord*. 235:544-550.

225. Lois G, Wessa M (2016): Differential association of default mode network connectivity and rumination in healthy individuals and remitted MDD patients. *Soc Cogn Affect Neurosci*. 11:1792-1801.

226. Lui S, Wu Q, Qiu L, Yang X, Kuang W, Chan RC, et al. (2011): Resting-state functional connectivity in treatment-resistant depression. *Am J Psychiatry*. 168:642-648.

227. Ma C, Ding J, Li J, Guo W, Long Z, Liu F, et al. (2012): Resting-state functional connectivity bias of middle temporal gyrus and caudate with altered gray matter volume in major depression. *PLoS One*. 7:e45263.

228. Pannekoek JN, van der Werff SJ, Meens PH, van den Bulk BG, Jolles DD, Veer IM, et al. (2014): Aberrant resting-state functional connectivity in limbic and salience networks in treatment--naïve clinically depressed adolescents. *J Child Psychol Psychiatry*. 55:1317-1327.

229. Peng D, Liddle EB, Iwabuchi SJ, Zhang C, Wu Z, Liu J, et al. (2015): Dissociated large-scale functional connectivity networks of the precuneus in medication-naïve first-episode depression. *Psychiatry Res*. 232:250-256.

230. Peng X, Lin P, Wu X, Gong R, Yang R, Wang J (2018): Insular subdivisions functional connectivity dysfunction within major depressive disorder. *J Affect Disord*. 227:280-288.

231. Penner J, Ford KA, Taylor R, Schaefer B, Théberge J, Neufeld RW, et al. (2016): Medial Prefrontal and Anterior Insular Connectivity in Early Schizophrenia and Major Depressive Disorder: A Resting Functional MRI Evaluation of Large-Scale Brain Network Models. *Front Hum Neurosci*. 10:132.

232. Peters AT, Burkhouse K, Feldhaus CC, Langenecker SA, Jacobs RH (2016): Aberrant resting-state functional connectivity in limbic and cognitive control networks relates to depressive rumination and mindfulness: A pilot study among adolescents with a history of depression. *J Affect Disord*. 200:178-181.

233. Philippi CL, Cornejo MD, Frost CP, Walsh EC, Hoks RM, Birn R, et al. (2018): Neural and behavioral correlates of negative self-focused thought associated with depression. *Hum Brain Mapp*. 39:2246-2257.

234. Poeppl TB, Müller VI, Hoffstaedter F, Bzdok D, Laird AR, Fox PT, et al. (2016): Imbalance in subregional connectivity of the right temporoparietal junction in major depression. *Hum Brain Mapp*. 37:2931-2942.

235. Rao JA, Jenkins LM, Hymen E, Feigon M, Weisenbach SL, Zubieta JK, et al. (2016): Differential Resting State Connectivity Patterns and Impaired Semantically Cued List Learning Test Performance in Early Course Remitted Major Depressive Disorder. *J Int Neuropsychol Soc*. 22:225-239.

236. Renner F, Siep N, Arntz A, van de Ven V, Peeters F, Quaedflieg C, et al. (2017): Negative mood-induction modulates default mode network resting-state functional connectivity in chronic depression. *J Affect Disord*. 208:590-596.

237. Sawaya H, Johnson K, Schmidt M, Arana A, Chahine G, Atoui M, et al. (2015): Resting-state functional connectivity of antero-medial prefrontal cortex sub-regions in major depression and relationship to emotional intelligence. *Int J Neuropsychopharmacol*. 18.

238. Scheinost D, Holmes SE, DellaGioia N, Schleifer C, Matuskey D, Abdallah CG, et al. (2018): Multimodal Investigation of Network Level Effects Using Intrinsic Functional Connectivity, Anatomical Covariance, and Structure-to-Function Correlations in Unmedicated Major Depressive Disorder. *Neuropsychopharmacology*. 43:1119-1127.
239. Schilbach L, Hoffstaedter F, Müller V, Cieslik EC, Goya-Maldonado R, Trost S, et al. (2016): Transdiagnostic commonalities and differences in resting state functional connectivity of the default mode network in schizophrenia and major depression. *Neuroimage Clin*. 10:326-335.
240. Sheline YI, Price JL, Yan Z, Mintun MA (2010): Resting-state functional MRI in depression unmasks increased connectivity between networks via the dorsal nexus. *Proc Natl Acad Sci U S A*. 107:11020-11025.
241. Shu H, Yuan Y, Xie C, Bai F, You J, Li L, et al. (2014): Imbalanced hippocampal functional networks associated with remitted geriatric depression and apolipoprotein E ε4 allele in nondemented elderly: a preliminary study. *J Affect Disord*. 164:5-13.
242. Späti J, Hänggi J, Doerig N, Ernst J, Sambataro F, Brakowski J, et al. (2015): Prefrontal thinning affects functional connectivity and regional homogeneity of the anterior cingulate cortex in depression. *Neuropsychopharmacology*. 40:1640-1648.
243. Straub J, Metzger CD, Plener PL, Koelch MG, Groen G, Abler B (2017): Successful group psychotherapy of depression in adolescents alters fronto-limbic resting-state connectivity. *J Affect Disord*. 209:135-139.
244. Tadayonnejad R, Ajilore O, Mickey BJ, Crane NA, Hsu DT, Kumar A, et al. (2016): Pharmacological modulation of pulvinar resting-state regional oscillations and network dynamics in major depression. *Psychiatry Res Neuroimaging*. 252:10-18.
245. Tahmasian M, Knight DC, Manoliu A, Schwerthöffer D, Scherr M, Meng C, et al. (2013): Aberrant intrinsic connectivity of hippocampus and amygdala overlap in the fronto-insular and dorsomedial-prefrontal cortex in major depressive disorder. *Front Hum Neurosci*. 7:639.
246. van Tol MJ, Li M, Metzger CD, Hailla N, Horn DI, Li W, et al. (2014): Local cortical thinning links to resting-state disconnectivity in major depressive disorder. *Psychol Med*. 44:2053-2065.
247. Wang Y, Chen G, Zhong S, Jia Y, Xia L, Lai S, et al. (2018): Association between resting-state brain functional connectivity and cortisol levels in unmedicated major depressive disorder. *J Psychiatr Res*. 105:55-62.
248. Workman CI, Lythe KE, McKie S, Moll J, Gethin JA, Deakin JF, et al. (2016): Subgenual Cingulate-Amygdala Functional Disconnection and Vulnerability to Melancholic Depression. *Neuropsychopharmacology*. 41:2082-2090.
249. Workman CI, Lythe KE, McKie S, Moll J, Gethin JA, Deakin JF, et al. (2017): A novel resting-state functional magnetic resonance imaging signature of resilience to recurrent depression. *Psychol Med*. 47:597-607.
250. Wu H, Sun H, Xu J, Wu Y, Wang C, Xiao J, et al. (2016): Changed Hub and Corresponding Functional Connectivity of Subgenual Anterior Cingulate Cortex in Major Depressive Disorder. *Front Neuroanat*. 10:120.
251. Wu X, Lin P, Yang J, Song H, Yang R, Yang J (2016): Dysfunction of the cingulo-opercular network in first-episode medication-naive patients with major depressive disorder. *J Affect Disord*. 200:275-283.
252. Yang Y, Zhong N, Imamura K, Lu S, Li M, Zhou H, et al. (2016): Task and Resting-State fMRI Reveal Altered Salience Responses to Positive Stimuli in Patients with Major Depressive Disorder. *PLoS One*. 11:e0155092.
253. Yang XH, Tian K, Wang DF, Wang Y, Cheung EFC, Xie GR, et al. (2017): Anhedonia correlates with abnormal functional connectivity of the superior temporal gyrus and the caudate nucleus in patients with first-episode drug-naive major depressive disorder. *J Affect Disord*. 218:284-290.

254. Ye T, Peng J, Nie B, Gao J, Liu J, Li Y, et al. (2012): Altered functional connectivity of the dorsolateral prefrontal cortex in first-episode patients with major depressive disorder. *Eur J Radiol*. 81:4035-4040.
255. Ye J, Shen Z, Xu X, Yang S, Chen W, Liu X, et al. (2017): Abnormal functional connectivity of the amygdala in first-episode and untreated adult major depressive disorder patients with different ages of onset. *Neuroreport*. 28:214-221.
256. Yin Y, Hou Z, Wang X, Sui Y, Yuan Y (2015): Association between altered resting-state cortico-cerebellar functional connectivity networks and mood/cognition dysfunction in late-onset depression. *J Neural Transm (Vienna)*. 122:887-896.
257. Yin Y, Hou Z, Wang X, Sui Y, Yuan Y (2015): The BDNF Val66Met polymorphism, resting-state hippocampal functional connectivity and cognitive deficits in acute late-onset depression. *J Affect Disord*. 183:22-30.
258. Yuan H, Young KD, Phillips R, Zotev V, Misaki M, Bodurka J (2014): Resting-state functional connectivity modulation and sustained changes after real-time functional magnetic resonance imaging neurofeedback training in depression. *Brain Connect*. 4:690-701.
259. Zhang YF, Han Y, Wang YZ, Zhang YF, Jia HX, Jin EH, et al. (2015): Characterization of resting-state fMRI-derived functional connectivity in patients with deficiency versus excess patterns of major depression. *Complement Ther Med*. 23:7-13.
260. Aghajani M, Veer IM, van Hoof MJ, Rombouts SA, van der Wee NJ, Vermeiren RR (2016): Abnormal functional architecture of amygdala-centered networks in adolescent posttraumatic stress disorder. *Hum Brain Mapp*. 37:1120-1135.
261. Andreescu C, Sheu LK, Tudorascu D, Walker S, Aizenstein H (2014): The ages of anxiety--differences across the lifespan in the default mode network functional connectivity in generalized anxiety disorder. *Int J Geriatr Psychiatry*. 29:704-712.
262. Arnold Anteraper S, Triantafyllou C, Sawyer AT, Hofmann SG, Gabrieli JD, Whitfield-Gabrieli S (2014): Hyper-connectivity of subcortical resting-state networks in social anxiety disorder. *Brain Connect*. 4:81-90.
263. Birn RM, Shackman AJ, Oler JA, Williams LE, McFarlin DR, Rogers GM, et al. (2014): Evolutionarily conserved prefrontal-amygdalar dysfunction in early-life anxiety. *Mol Psychiatry*. 19:915-922.
264. Bluhm RL, Williamson PC, Osuch EA, Frewen PA, Stevens TK, Boksman K, et al. (2009): Alterations in default network connectivity in posttraumatic stress disorder related to early-life trauma. *J Psychiatry Neurosci*. 34:187-194.
265. Brown VM, LaBar KS, Haswell CC, Gold AL, McCarthy G, Morey RA (2014): Altered resting-state functional connectivity of basolateral and centromedial amygdala complexes in posttraumatic stress disorder. *Neuropsychopharmacology*. 39:351-359.
266. Chen HJ, Zhang L, Ke J, Qi R, Xu Q, Zhong Y, et al. (2019): Altered resting-state dorsal anterior cingulate cortex functional connectivity in patients with post-traumatic stress disorder. *Aust N Z J Psychiatry*. 53:68-79.
267. Cui Q, Vanman EJ, Long Z, Pang Y, Chen Y, Wang Y, et al. (2017): Social anxiety disorder exhibit impaired networks involved in self and theory of mind processing. *Soc Cogn Affect Neurosci*. 12:1284-1295.
268. Dodhia S, Hosanagar A, Fitzgerald DA, Labuschagne I, Wood AG, Nathan PJ, et al. (2014): Modulation of resting-state amygdala-frontal functional connectivity by oxytocin in generalized social anxiety disorder. *Neuropsychopharmacology*. 39:2061-2069.
269. Dorfman J, Benson B, Farber M, Pine D, Ernst M (2016): Altered striatal intrinsic functional connectivity in pediatric anxiety. *Neuropsychologia*. 85:159-168.
270. Geiger MJ, Domschke K, Ipser J, Hattingh C, Baldwin DS, Lochner C, et al. (2016): Altered executive control network resting-state connectivity in social anxiety disorder. *World J Biol Psychiatry*. 17:47-57.

271. Hahn A, Stein P, Windischberger C, Weissenbacher A, Spindelegger C, Moser E, et al. (2011): Reduced resting-state functional connectivity between amygdala and orbitofrontal cortex in social anxiety disorder. *Neuroimage*. 56:881-889.
272. Hamm LL, Jacobs RH, Johnson MW, Fitzgerald DA, Fitzgerald KD, Langenecker SA, et al. (2014): Aberrant amygdala functional connectivity at rest in pediatric anxiety disorders. *Biol Mood Anxiety Disord*. 4:15.
273. Harricharan S, Rabellino D, Frewen PA, Densmore M, Théberge J, McKinnon MC, et al. (2016): fMRI functional connectivity of the periaqueductal gray in PTSD and its dissociative subtype. *Brain Behav*. 6:e00579.
274. Harricharan S, Nicholson AA, Densmore M, Théberge J, McKinnon MC, Neufeld RWJ, et al. (2017): Sensory overload and imbalance: Resting-state vestibular connectivity in PTSD and its dissociative subtype. *Neuropsychologia*. 106:169-178.
275. Holmes SE, Scheinost D, DellaGioia N, Davis MT, Matuskey D, Pietrzak RH, et al. (2018): Cerebellar and prefrontal cortical alterations in PTSD: structural and functional evidence. *Chronic Stress (Thousand Oaks)*. 2.
276. Jung YH, Shin JE, Lee YI, Jang JH, Jo HJ, Choi SH (2018): Altered Amygdala Resting-State Functional Connectivity and Hemispheric Asymmetry in Patients With Social Anxiety Disorder. *Front Psychiatry*. 9:164.
277. Li W, Cui H, Zhu Z, Kong L, Guo Q, Zhu Y, et al. (2016): Aberrant Functional Connectivity between the Amygdala and the Temporal Pole in Drug-Free Generalized Anxiety Disorder. *Front Hum Neurosci*. 10:549.
278. Liao W, Xu Q, Mantini D, Ding J, Machado-de-Sousa JP, Hallak JE, et al. (2011): Altered gray matter morphometry and resting-state functional and structural connectivity in social anxiety disorder. *Brain Res*. 1388:167-177.
279. Liu WJ, Yin DZ, Cheng WH, Fan MX, You MN, Men WW, et al. (2015): Abnormal functional connectivity of the amygdala-based network in resting-state FMRI in adolescents with generalized anxiety disorder. *Med Sci Monit*. 21:459-467.
280. Makovac E, Meeten F, Watson DR, Herman A, Garfinkel SN, H DC, et al. (2016): Alterations in Amygdala-Prefrontal Functional Connectivity Account for Excessive Worry and Autonomic Dysregulation in Generalized Anxiety Disorder. *Biol Psychiatry*. 80:786-795.
281. Manning J, Reynolds G, Saygin ZM, Hofmann SG, Pollack M, Gabrieli JD, et al. (2015): Altered resting-state functional connectivity of the frontal-striatal reward system in social anxiety disorder. *PLoS One*. 10:e0125286.
282. Misaki M, Phillips R, Zotev V, Wong CK, Wurfel BE, Krueger F, et al. (2018): Connectome-wide investigation of altered resting-state functional connectivity in war veterans with and without posttraumatic stress disorder. *Neuroimage Clin*. 17:285-296.
283. Nicholson AA, Densmore M, Frewen PA, Théberge J, Neufeld RW, McKinnon MC, et al. (2015): The Dissociative Subtype of Posttraumatic Stress Disorder: Unique Resting-State Functional Connectivity of Basolateral and Centromedial Amygdala Complexes. *Neuropsychopharmacology*. 40:2317-2326.
284. Pace-Schott EF, Zimmerman JP, Bottary RM, Lee EG, Milad MR, Camprodon JA (2017): Resting state functional connectivity in primary insomnia, generalized anxiety disorder and controls. *Psychiatry Res Neuroimaging*. 265:26-34.
285. Pannekoek JN, Veer IM, van Tol MJ, van der Werff SJ, Demenescu LR, Aleman A, et al. (2013): Resting-state functional connectivity abnormalities in limbic and salience networks in social anxiety disorder without comorbidity. *Eur Neuropsychopharmacol*. 23:186-195.
286. Pannekoek JN, Veer IM, van Tol MJ, van der Werff SJ, Demenescu LR, Aleman A, et al. (2013): Aberrant limbic and salience network resting-state functional connectivity in panic disorder without comorbidity. *J Affect Disord*. 145:29-35.
287. Patriat R, Birn RM, Keding TJ, Herringa RJ (2016): Default-Mode Network Abnormalities in Pediatric Posttraumatic Stress Disorder. *J Am Acad Child Adolesc Psychiatry*. 55:319-327.

288. Prater KE, Hosanagar A, Klumpp H, Angstadt M, Phan KL (2013): Aberrant amygdala-frontal cortex connectivity during perception of fearful faces and at rest in generalized social anxiety disorder. *Depress Anxiety*. 30:234-241.
289. Rabellino D, Densmore M, Théberge J, McKinnon MC, Lanius RA (2018): The cerebellum after trauma: Resting-state functional connectivity of the cerebellum in posttraumatic stress disorder and its dissociative subtype. *Hum Brain Mapp*. 39:3354-3374.
290. Rabinak CA, Angstadt M, Welsh RC, Kenndy AE, Lyubkin M, Martis B, et al. (2011): Altered amygdala resting-state functional connectivity in post-traumatic stress disorder. *Front Psychiatry*. 2:62.
291. Roy AK, Fudge JL, Kelly C, Perry JS, Daniele T, Carlisi C, et al. (2013): Intrinsic functional connectivity of amygdala-based networks in adolescent generalized anxiety disorder. *J Am Acad Child Adolesc Psychiatry*. 52:290-299.e292.
292. Sripada RK, King AP, Garfinkel SN, Wang X, Sripada CS, Welsh RC, et al. (2012): Altered resting-state amygdala functional connectivity in men with posttraumatic stress disorder. *J Psychiatry Neurosci*. 37:241-249.
293. Sripada RK, King AP, Welsh RC, Garfinkel SN, Wang X, Sripada CS, et al. (2012): Neural dysregulation in posttraumatic stress disorder: evidence for disrupted equilibrium between salience and default mode brain networks. *Psychosom Med*. 74:904-911.
294. Thome J, Densmore M, Frewen PA, McKinnon MC, Théberge J, Nicholson AA, et al. (2017): Desynchronization of autonomic response and central autonomic network connectivity in posttraumatic stress disorder. *Hum Brain Mapp*. 38:27-40.
295. Toazza R, Franco AR, Buchweitz A, Molle RD, Rodrigues DM, Reis RS, et al. (2016): Amygdala-based intrinsic functional connectivity and anxiety disorders in adolescents and young adults. *Psychiatry Res Neuroimaging*. 257:11-16.
296. Wang W, Hou J, Qian S, Liu K, Li B, Li M, et al. (2016): Aberrant regional neural fluctuations and functional connectivity in generalized anxiety disorder revealed by resting-state functional magnetic resonance imaging. *Neurosci Lett*. 624:78-84.
297. Yuan M, Zhu H, Qiu C, Meng Y, Zhang Y, Shang J, et al. (2016): Group cognitive behavioral therapy modulates the resting-state functional connectivity of amygdala-related network in patients with generalized social anxiety disorder. *BMC Psychiatry*. 16:198.
298. Yuan C, Zhu H, Ren Z, Yuan M, Gao M, Zhang Y, et al. (2018): Precuneus-related regional and network functional deficits in social anxiety disorder: A resting-state functional MRI study. *Compr Psychiatry*. 82:22-29.
299. MinlanYuan, Meng Y, Zhang Y, Nie X, Ren Z, Zhu H, et al. (2017): Cerebellar Neural Circuits Involving Executive Control Network Predict Response to Group Cognitive Behavior Therapy in Social Anxiety Disorder. *Cerebellum*. 16:673-682.
300. Zhang X, Zhang J, Wang L, Li R, Zhang W (2016): Altered resting-state functional connectivity of the amygdala in Chinese earthquake survivors. *Prog Neuropsychopharmacol Biol Psychiatry*. 65:208-214.
301. Zhu X, Helpman L, Papini S, Schneier F, Markowitz JC, Van Meter PE, et al. (2017): Altered resting state functional connectivity of fear and reward circuitry in comorbid PTSD and major depression. *Depress Anxiety*. 34:641-650.
302. Ayoub LJ, Barnett A, Leboucher A, Golosky M, McAndrews MP, Seminowicz DA, et al. (2019): The medial temporal lobe in nociception: a meta-analytic and functional connectivity study. *Pain*. 160:1245-1260.
303. Baliki MN, Baria AT, Apkarian AV (2011): The cortical rhythms of chronic back pain. *J Neurosci*. 31:13981-13990.
304. Bhatt RR, Gupta A, Labus JS, Zeltzer LK, Tsao JC, Shulman RJ, et al. (2019): Altered Brain Structure and Functional Connectivity and Its Relation to Pain Perception in Girls With Irritable Bowel Syndrome. *Psychosom Med*. 81:146-154.
305. Bolwerk A, Seifert F, Maihöfner C (2013): Altered resting-state functional connectivity in complex regional pain syndrome. *J Pain*. 14:1107-1115.e1108.

306. Case M, Zhang H, Mundahl J, Datta Y, Nelson S, Gupta K, et al. (2017): Characterization of functional brain activity and connectivity using EEG and fMRI in patients with sickle cell disease. *Neuroimage Clin*. 14:1-17.
307. Cauda F, Sacco K, D'Agata F, Duca S, Cocito D, Geminiani G, et al. (2009): Low-frequency BOLD fluctuations demonstrate altered thalamocortical connectivity in diabetic neuropathic pain. *BMC Neurosci*. 10:138.
308. Čeko M, Shir Y, Ouellet JA, Ware MA, Stone LS, Seminowicz DA (2015): Partial recovery of abnormal insula and dorsolateral prefrontal connectivity to cognitive networks in chronic low back pain after treatment. *Hum Brain Mapp*. 36:2075-2092.
309. Chen Z, Chen X, Liu M, Liu S, Shu S, Ma L, et al. (2016): Altered functional connectivity of the marginal division in migraine: a resting-state fMRI study. *J Headache Pain*. 17:89.
310. Cottam WJ, Iwabuchi SJ, Drabek MM, Reckziegel D, Auer DP (2018): Altered connectivity of the right anterior insula drives the pain connectome changes in chronic knee osteoarthritis. *Pain*. 159:929-938.
311. Fallon N, Chiu Y, Nurmikko T, Stancak A (2016): Functional Connectivity with the Default Mode Network Is Altered in Fibromyalgia Patients. *PLoS One*. 11:e0159198.
312. Flodin P, Martinsen S, Löfgren M, Bileviciute-Ljungar I, Kosek E, Fransson P (2014): Fibromyalgia is associated with decreased connectivity between pain- and sensorimotor brain areas. *Brain Connect*. 4:587-594.
313. Flodin P, Martinsen S, Altawil R, Waldheim E, Lampa J, Kosek E, et al. (2016): Intrinsic Brain Connectivity in Chronic Pain: A Resting-State fMRI Study in Patients with Rheumatoid Arthritis. *Front Hum Neurosci*. 10:107.
314. Duke Han S, Buchman AS, Arfanakis K, Fleischman DA, Bennett DA (2013): Functional connectivity networks associated with chronic musculoskeletal pain in old age. *Int J Geriatr Psychiatry*. 28:858-867.
315. He S, Li F, Gu T, Ma H, Li X, Zou S, et al. (2018): Reduced corticostriatal functional connectivity in temporomandibular disorders. *Hum Brain Mapp*. 39:2563-2572.
316. Hong JY, Kilpatrick LA, Labus JS, Gupta A, Katibian D, Ashe-McNalley C, et al. (2014): Sex and disease-related alterations of anterior insula functional connectivity in chronic abdominal pain. *J Neurosci*. 34:14252-14259.
317. Ichesco E, Quintero A, Clauw DJ, Peltier S, Sundgren PM, Gerstner GE, et al. (2012): Altered functional connectivity between the insula and the cingulate cortex in patients with temporomandibular disorder: a pilot study. *Headache*. 52:441-454.
318. Ichesco E, Schmidt-Wilcke T, Bhavsar R, Clauw DJ, Peltier SJ, Kim J, et al. (2014): Altered resting state connectivity of the insular cortex in individuals with fibromyalgia. *J Pain*. 15:815-826.e811.
319. Jiang J, Gu L, Bao D, Hong S, He W, Tan Y, et al. (2016): Altered homotopic connectivity in postherpetic neuralgia: a resting state fMRI study. *J Pain Res*. 9:877-886.
320. Khan SA, Keaser ML, Meiller TF, Seminowicz DA (2014): Altered structure and function in the hippocampus and medial prefrontal cortex in patients with burning mouth syndrome. *Pain*. 155:1472-1480.
321. Kilpatrick LA, Kutch JJ, Tillisch K, Naliboff BD, Labus JS, Jiang Z, et al. (2014): Alterations in resting state oscillations and connectivity in sensory and motor networks in women with interstitial cystitis/painful bladder syndrome. *J Urol*. 192:947-955.
322. Kim JH, Choi SH, Jang JH, Lee DH, Lee KJ, Lee WJ, et al. (2017): Impaired insula functional connectivity associated with persistent pain perception in patients with complex regional pain syndrome. *PLoS One*. 12:e0180479.
323. Kong J, Wolcott E, Wang Z, Jorgenson K, Harvey WF, Tao J, et al. (2019): Altered resting state functional connectivity of the cognitive control network in fibromyalgia and the modulation effect of mind-body intervention. *Brain Imaging Behav*. 13:482-492.

324. Kucyi A, Moayedi M, Weissman-Fogel I, Goldberg MB, Freeman BV, Tenenbaum HC, et al. (2014): Enhanced medial prefrontal-default mode network functional connectivity in chronic pain and its association with pain rumination. *J Neurosci*. 34:3969-3975.
325. Kutch JJ, Yani MS, Asavasopon S, Kirages DJ, Rana M, Cosand L, et al. (2015): Altered resting state neuromotor connectivity in men with chronic prostatitis/chronic pelvic pain syndrome: A MAPP: Research Network Neuroimaging Study. *Neuroimage Clin*. 8:493-502.
326. Li Z, Liu M, Lan L, Zeng F, Makris N, Liang Y, et al. (2016): Altered periaqueductal gray resting state functional connectivity in migraine and the modulation effect of treatment. *Sci Rep*. 6:20298.
327. Li Z, Lan L, Zeng F, Makris N, Hwang J, Guo T, et al. (2017): The altered right frontoparietal network functional connectivity in migraine and the modulation effect of treatment. *Cephalalgia*. 37:161-176.
328. Liu P, Wang G, Liu Y, Zeng F, Lin D, Yang X, et al. (2017): Disrupted intrinsic connectivity of the periaqueductal gray in patients with functional dyspepsia: A resting-state fMRI study. *Neurogastroenterol Motil*. 29.
329. Martucci KT, Shirer WR, Bagarinao E, Johnson KA, Farmer MA, Labus JS, et al. (2015): The posterior medial cortex in urologic chronic pelvic pain syndrome: detachment from default mode network-a resting-state study from the MAPP Research Network. *Pain*. 156:1755-1764.
330. Michels L, Christidi F, Steiger VR, Sándor PS, Gantenbein AR, Landmann G, et al. (2017): Pain modulation is affected differently in medication-overuse headache and chronic myofascial pain - A multimodal MRI study. *Cephalalgia*. 37:764-779.
331. Niddam DM, Lai KL, Fuh JL, Chuang CY, Chen WT, Wang SJ (2016): Reduced functional connectivity between salience and visual networks in migraine with aura. *Cephalalgia*. 36:53-66.
332. Pujol J, Macià D, Garcia-Fontanals A, Blanco-Hinojo L, López-Solà M, Garcia-Blanco S, et al. (2014): The contribution of sensory system functional connectivity reduction to clinical pain in fibromyalgia. *Pain*. 155:1492-1503.
333. Qi R, Liu C, Ke J, Xu Q, Ye Y, Jia L, et al. (2016): Abnormal Amygdala Resting-State Functional Connectivity in Irritable Bowel Syndrome. *AJNR Am J Neuroradiol*. 37:1139-1145.
334. Truini A, Tinelli E, Gerardi MC, Calistri V, Iannuccelli C, La Cesa S, et al. (2016): Abnormal resting state functional connectivity of the periaqueductal grey in patients with fibromyalgia. *Clin Exp Rheumatol*. 34:S129-133.
335. Wei SY, Chao HT, Tu CH, Lin MW, Li WC, Low I, et al. (2016): The BDNF Val66Met polymorphism is associated with the functional connectivity dynamics of pain modulatory systems in primary dysmenorrhea. *Sci Rep*. 6:23639.
336. Wei SY, Chao HT, Tu CH, Li WC, Low I, Chuang CY, et al. (2016): Changes in functional connectivity of pain modulatory systems in women with primary dysmenorrhea. *Pain*. 157:92-102.
337. Wei SY, Chen LF, Lin MW, Li WC, Low I, Yang CJ, et al. (2017): The OPRM1 A118G polymorphism modulates the descending pain modulatory system for individual pain experience in young women with primary dysmenorrhea. *Sci Rep*. 7:39906.
338. Wei HL, Chen J, Chen YC, Yu YS, Zhou GP, Qu LJ, et al. (2020): Impaired functional connectivity of limbic system in migraine without aura. *Brain Imaging Behav*. 14:1805-1814.
339. Yu ZB, Lv YB, Song LH, Liu DH, Huang XL, Hu XY, et al. (2017): Functional Connectivity Differences in the Insular Sub-regions in Migraine without Aura: A Resting-State Functional Magnetic Resonance Imaging Study. *Front Behav Neurosci*. 11:124.
340. Zhang J, Su J, Wang M, Zhao Y, Zhang QT, Yao Q, et al. (2017): The sensorimotor network dysfunction in migraineurs without aura: a resting-state fMRI study. *J Neurol*. 264:654-663.
341. Zhang Y, Mao Z, Pan L, Ling Z, Liu X, Zhang J, et al. (2019): Frequency-specific alterations in cortical rhythms and functional connectivity in trigeminal neuralgia. *Brain Imaging Behav*. 13:1497-1509.

342. Hagan CC, Graham JM, Tait R, Widmer B, van Nieuwenhuizen AO, Ooi C, et al. (2015): Adolescents with current major depressive disorder show dissimilar patterns of age-related differences in ACC and thalamus. *Neuroimage Clin*. 7:391-399.
343. Biedermann SV, Weber-Fahr W, Demirakca T, Tunc-Skarka N, Hoerst M, Henn F, et al. (2015): 31P RINEPT MRSI and VBM reveal alterations in brain aging associated with major depression. *Magn Reson Med*. 73:1390-1400.
344. Jia Z, Huang X, Wu Q, Zhang T, Lui S, Zhang J, et al. (2010): High-field magnetic resonance imaging of suicidality in patients with major depressive disorder. *Am J Psychiatry*. 167:1381-1390.
345. Treadway MT, Grant MM, Ding Z, Hollon SD, Gore JC, Shelton RC (2009): Early adverse events, HPA activity and rostral anterior cingulate volume in MDD. *PLoS One*. 4:e4887.
346. Yoshikawa E, Matsuoka Y, Yamasue H, Inagaki M, Nakano T, Akechi T, et al. (2006): Prefrontal cortex and amygdala volume in first minor or major depressive episode after cancer diagnosis. *Biol Psychiatry*. 59:707-712.
347. Colloby SJ, Firbank MJ, Vasudev A, Parry SW, Thomas AJ, O'Brien JT (2011): Cortical thickness and VBM-DARTEL in late-life depression. *J Affect Disord*. 133:158-164.
348. Koolschijn PC, van Haren NE, Schnack HG, Janssen J, Hulshoff Pol HE, Kahn RS (2010): Cortical thickness and voxel-based morphometry in depressed elderly. *Eur Neuropsychopharmacol*. 20:398-404.
349. Szabó C, Kelemen O, Levy-Gigi E, Kéri S (2015): Acute response to psychological trauma and subsequent recovery: no changes in brain structure. *Psychiatry Res*. 231:269-272.
350. Moon CM, Kang HK, Jeong GW (2015): Metabolic change in the right dorsolateral prefrontal cortex and its correlation with symptom severity in patients with generalized anxiety disorder: Proton magnetic resonance spectroscopy at 3 Tesla. *Psychiatry Clin Neurosci*. 69:422-430.
351. Jatzko A, Rothenhöfer S, Schmitt A, Gaser C, Demirakca T, Weber-Fahr W, et al. (2006): Hippocampal volume in chronic posttraumatic stress disorder (PTSD): MRI study using two different evaluation methods. *J Affect Disord*. 94:121-126.
352. Eckart C, Stoppel C, Kaufmann J, Tempelmann C, Hinrichs H, Elbert T, et al. (2011): Structural alterations in lateral prefrontal, parietal and posterior midline regions of men with chronic posttraumatic stress disorder. *J Psychiatry Neurosci*. 36:176-186.
353. Yu CX, Ji TT, Song H, Li B, Han Q, Li L, et al. (2017): Abnormality of spontaneous brain activities in patients with chronic neck and shoulder pain: A resting-state fMRI study. *J Int Med Res*. 45:182-192.
354. Chen B, He Y, Xia L, Guo LL, Zheng JL (2016): Cortical plasticity between the pain and pain-free phases in patients with episodic tension-type headache. *J Headache Pain*. 17:105.
355. Hougaard A (2015): Investigations of functional and structural changes in migraine with aura by magnetic resonance imaging. *Dan Med J*. 62:B5129.
356. Tessitore A, Russo A, Giordano A, Conte F, Corbo D, De Stefano M, et al. (2013): Disrupted default mode network connectivity in migraine without aura. *J Headache Pain*. 14:89.
357. Dolman AJ, Loggia ML, Edwards RR, Gollub RL, Kong J, Napadow V, et al. (2014): Phenotype matters: the absence of a positive association between cortical thinning and chronic low back pain when controlling for salient clinical variables. *Clin J Pain*. 30:839-845.
358. Seminowicz DA, Shpaner M, Keaser ML, Krauthamer GM, Mantegna J, Dumas JA, et al. (2013): Cognitive-behavioral therapy increases prefrontal cortex gray matter in patients with chronic pain. *J Pain*. 14:1573-1584.
359. Russo A, Tessitore A, Giordano A, Corbo D, Marcuccio L, De Stefano M, et al. (2012): Executive resting-state network connectivity in migraine without aura. *Cephalalgia*. 32:1041-1048.
360. Farmer MA, Chanda ML, Parks EL, Baliki MN, Apkarian AV, Schaeffer AJ (2011): Brain functional and anatomical changes in chronic prostatitis/chronic pelvic pain syndrome. *J Urol*. 186:117-124.

361. Hsu MC, Harris RE, Sundgren PC, Welsh RC, Fernandes CR, Clauw DJ, et al. (2009): No consistent difference in gray matter volume between individuals with fibromyalgia and age-matched healthy subjects when controlling for affective disorder. *Pain*. 143:262-267.
362. Celle S, Créac'h C, Boutet C, Roche F, Chouchou F, Barthélémy JC, et al. (2018): Elderly Patients with Ongoing Migraine Show Reduced Gray Matter Volume in Second Somatosensory Cortex. *J Oral Facial Pain Headache*. 32:67–74.
363. Ung H, Brown JE, Johnson KA, Younger J, Hush J, Mackey S (2014): Multivariate classification of structural MRI data detects chronic low back pain. *Cereb Cortex*. 24:1037-1044.
364. Demenescu LR, Colic L, Li M, Safron A, Biswal B, Metzger CD, et al. (2017): A spectroscopic approach toward depression diagnosis: local metabolism meets functional connectivity. *Eur Arch Psychiatry Clin Neurosci*. 267:95-105.
365. Brunetti M, Marzetti L, Sepede G, Zappasodi F, Pizzella V, Sarchione F, et al. (2017): Resilience and cross-network connectivity: A neural model for post-trauma survival. *Prog Neuropsychopharmacol Biol Psychiatry*. 77:110-119.
366. Olivé I, Densmore M, Harricharan S, Théberge J, McKinnon MC, Lanius R (2018): Superior colliculus resting state networks in post-traumatic stress disorder and its dissociative subtype. *Hum Brain Mapp*. 39:563-574.
367. Schwedt TJ, Larson-Prior L, Coalson RS, Nolan T, Mar S, Ances BM, et al. (2014): Allodynia and descending pain modulation in migraine: a resting state functional connectivity analysis. *Pain Med*. 15:154-165.
